# α,ω-Diacyl-Substituted Analogues of Natural and Unnatural Polyamines: Identification of Potent Bactericides That Selectively Target Bacterial Membranes

**DOI:** 10.3390/ijms24065882

**Published:** 2023-03-20

**Authors:** Dan Chen, Melissa M. Cadelis, Florent Rouvier, Thomas Troia, Liam R. Edmeades, Kyle Fraser, Evangelene S. Gill, Marie-Lise Bourguet-Kondracki, Jean Michel Brunel, Brent R. Copp

**Affiliations:** 1 School of Chemical Sciences, The University of Auckland, Private Bag 92019, Auckland 1142, New Zealand; 2 UMR MD1 “Membranes et Cibles Therapeutiques”, U1261 INSERM, Faculté de Pharmacie, Aix-Marseille Universite, 27 bd Jean Moulin, 13385 Marseille, France; 3 Laboratoire Molécules de Communication et Adaptation des Micro-Organismes, UMR 7245 CNRS, Muséum National d’Histoire Naturelle, 57 Rue Cuvier (C.P. 54), 75005 Paris, France

**Keywords:** antimicrobial activities, polyamine conjugates, thiourea, antibiotic enhancement

## Abstract

In this study, α-ω-disubstituted polyamines exhibit a range of potentially useful biological activities, including antimicrobial and antibiotic potentiation properties. We have prepared an expanded set of diarylbis(thioureido)polyamines that vary in central polyamine core length, identifying analogues with potent methicillin-resistant *Staphylococcus aureus* (MRSA), *Escherichia coli*, *Acinetobacter baumannii* and *Candida albicans* growth inhibition properties, in addition to the ability to enhance action of doxycycline towards Gram-negative bacterium *Pseudomonas aeruginosa*. The observation of associated cytotoxicity/hemolytic properties prompted synthesis of an alternative series of diacylpolyamines that explored aromatic head groups of varying lipophilicity. Examples bearing terminal groups each containing two phenyl rings (**15a**–**f**, **16a**–**f**) were found to have optimal intrinsic antimicrobial properties, with MRSA being the most susceptible organism. A lack of observed cytotoxicity or hemolytic properties for all but the longest polyamine chain variants identified these as non-toxic Gram-positive antimicrobials worthy of further study. Analogues bearing either one or three aromatic-ring-containing head groups were either generally devoid of antimicrobial properties (one ring) or cytotoxic/hemolytic (three rings), defining a rather narrow range of head group lipophilicity that affords selectivity for Gram-positive bacterial membranes versus mammalian. Analogue **15d** is bactericidal and targets the Gram-positive bacterial membrane.

## 1. Introduction

**Figure 1:**
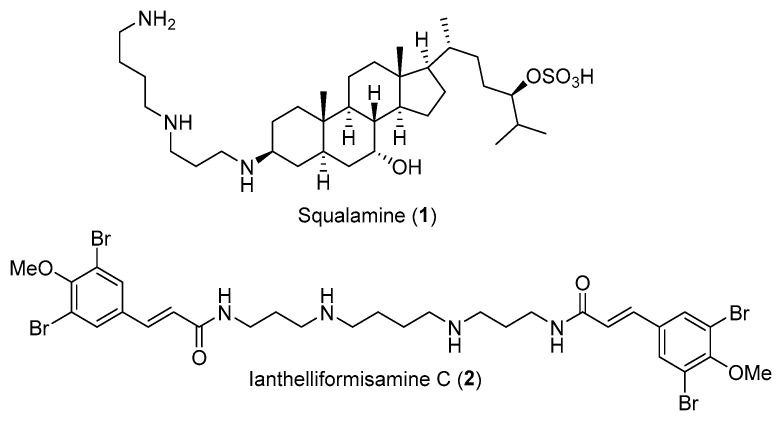
Structures of polyamine natural products **1** and **2**.

Biogenic polyamines (PA) can disrupt bacterial membranes, inhibit formation of biofilms and act as adjuvants to increase activity of antibiotics towards resistant bacteria [1,2]. While simple polyamines such as spermidine and spermine (PA-3-4-3) have weak mM potency, more elaborately functionalized polyamine analogues, including natural products such as squalamine (**1**) [3,4,5,6] and ianthelliformisamine C (**2**) [7,8,9] (Figure 1), are significantly more active, exhibiting enhanced ability to disrupt and/or depolarize bacterial membranes and the enhance action of different classes of antibiotics towards Gram-negative bacteria. Examples of polyamine terminal substituents that imbue intrinsic antimicrobial activities and/or antibiotic adjuvant properties include cinnamic acids [8,9], thioureas [10], indole derivatives [11,12,13] and lipids, including alkanes/alkenes [14,15,16,17], sterols [18,19,20], diterpenes [21] and triterpenes [18,22]. Presence of secondary alkylamines in the polyamine chain, protonated at physiological pH and an essential requirement for activity [10,11], combined with lipophilic end-groups, represents an amphipathic motif satisfying the minimal pharmacophore of synthetic mimics of antimicrobial peptides [23,24,25,26]. While satisfying this pharmacophore model, it is still not clear as to what chemometric attributes the terminal substituent(s) require as far as intrinsic antimicrobial, antibiotic adjuvant or cytotoxic/hemolytic properties.

**Figure 2:**
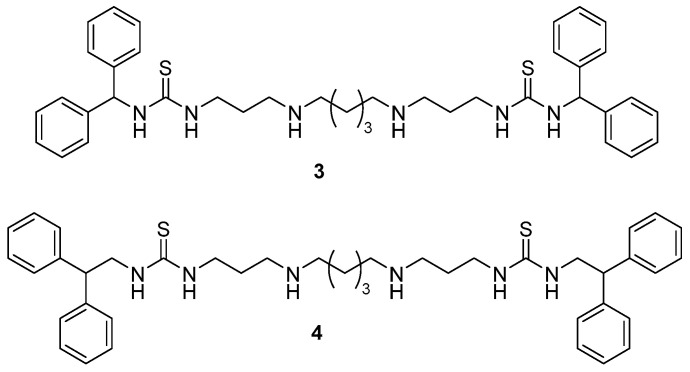
Structures of bis(thioureido) polyamines **3** and **4**.

Of note was the recent report of urea- and thiourea- functionalized polyamines, including **3** and **4** (Figure 2), which exhibited broad-spectrum antimicrobial properties towards both Gram-positive and Gram-negative bacteria, with a mechanism attributed to depolarization of the cytoplasmic membrane and permeabilization of the bacterial outer membrane [10]. Structure-relationship analysis identified essentiality of the mid-chain secondary amines for activity, that diaryl aromatic head groups were more active than mono-aryl, thioureas were equipotent to the corresponding ureas and, from a limited dataset of polyamine midchain lengths, there was little variation in activity between PA-3-4-3 and PA-3-5-3 variants. Closer investigation of the biological properties of **4** identified potent activity towards methicillin-resistant *Staphylococcus aureus* (MRSA), only weak hemolytic and cytotoxic properties, the ability to depolarize bacterial membrane potential and increase bacterial membrane permeability and to act synergistically with kanamycin towards *S. aureus*.

In continuation of our ongoing interest in discovery of polyamine derivatives that exhibit antibacterial and antibiotic adjuvant properties [11,12,13,21,27], we have prepared a set of five additional analogues of thiourea **3** to explore the effect of polyamine chain length on activity and a further set of twenty-four analogues that explore variation in the thiourea linking group and changes to end-group lipophilicity. All analogues were evaluated for antimicrobial activities against a set of Gram-positive and Gram-negative bacteria and for the ability to enhance the antibiotic activity of doxycycline towards Gram-negative bacteria *Pseudomonas aeruginosa*.

### Chemistry

**Figure 3:**
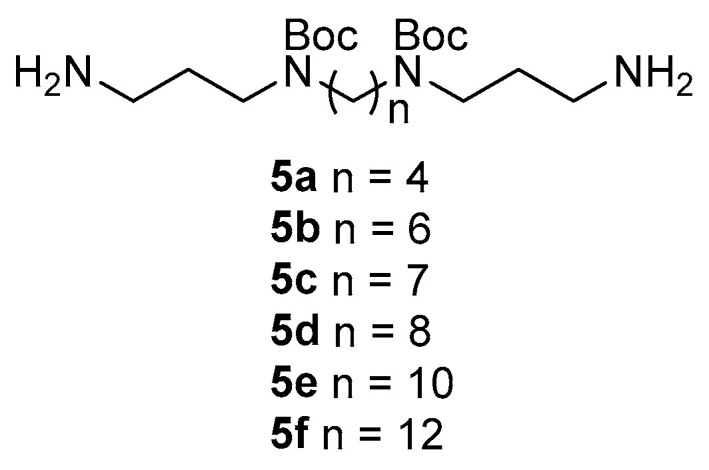
Boc-protected polyamines **5a**–**f**.

The initial requirement was synthesis of Boc-protected polyamine scaffolds **5a**–**f** (Figure 3), methodology of which has been previously reported [28,29,30,31]. Six polyamines of varying chain lengths, ranging from spermine (polyamine PA-3-4-3) to the longer and more lipophilic PA-3-12-3 chain, were synthesized to examine the influence of a variety of parameters (chain length, lipophilicity, steric bulk and spatial positioning of the positive charges) on bioactivity.

**Scheme 1:**
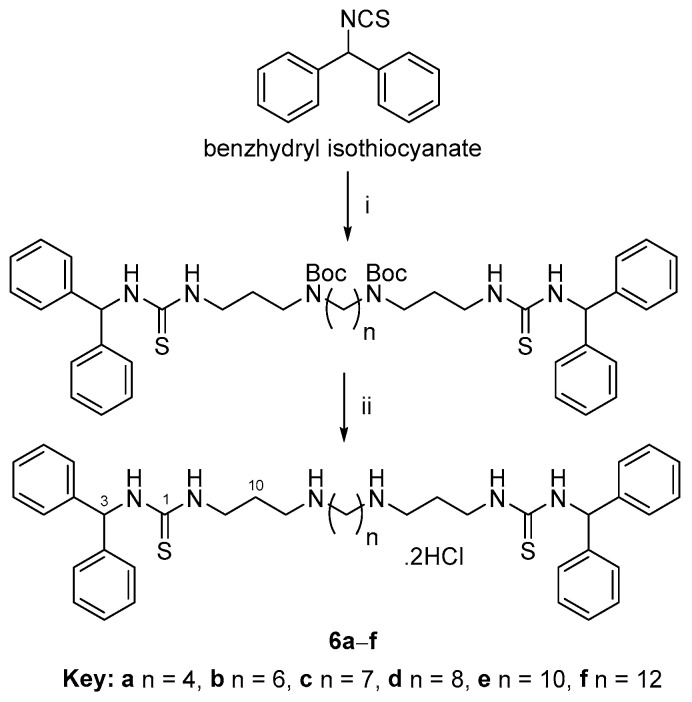
General synthesis of thiourea polyamine analogues **6a**–**f**. *Reagents and conditions*: (i) benzhydryl isothiocyanate (2.2 equiv.), Boc-protected polyamine (**5a**–**f**) (1 equiv.), CH_2_Cl_2_, 0 °C, 18 h (yields 55–87%); (ii) 1 M HCl in EtOAc, r.t., 12 h (yields 84–94%).

The first set of analogues, thioureas **6a**–**f**, were synthesized using an established pathway, as shown in Scheme 1 [10]. Reaction of commercially available benzhydryl isothiocyanate with polyamines **5a**–**f** gave Boc-protected intermediates that were then deprotected using 1 M HCl in EtOAc to afford desired polyamine compounds **6a**–**f** as their di-HCl salts (Figures S1–S6). Derivative **6a** has been reported previously [10].

**Figure 4:**
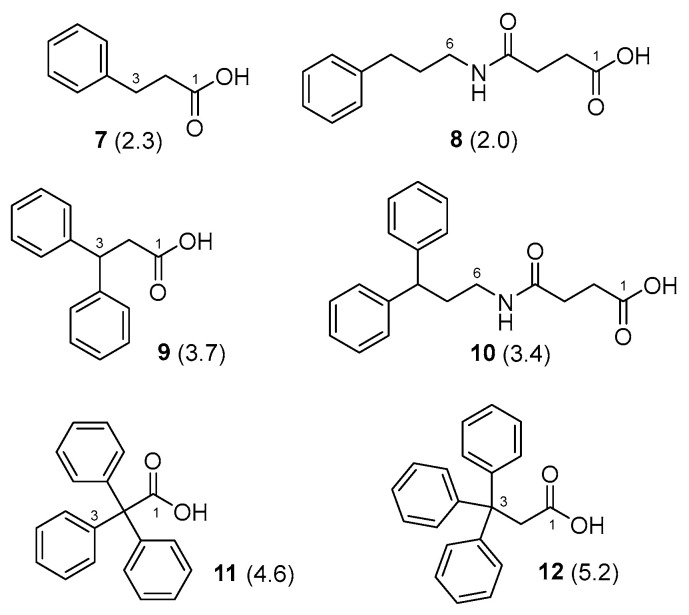
Structures of aromatic head groups **7**–**12** with cLogP values of the corresponding methyl ester in parentheses.

To explore the effect of variation in thiourea linking group and changes to end-group lipophilicity on biological activity of **6a**–**f**, an expanded set of amide-linked polyamine derivatives were prepared that made use of aromatic head groups 3-phenylpropanoic acid (**7**), 4-oxo-4-((3-phenylpropyl)amino)butanoic acid (**8**) (Figure S7), 3,3-diphenylpropanoic acid (**9**), 4-((3,3-diphenylpropyl)amino)-4-oxobutanoic acid (**10**) (Figure S8), 2,2,2-triphenylacetic acid (**11**) and 3,3,3-triphenylpropanoic acid (**12**) (Figure 4). Of these six acids, **8** and **10** were not commercially available: they were prepared by reaction of 3-phenylpropylamine and 3,3-diphenylpropylamine with succinic anhydride in yields of 41% and 97%, respectively.

Reaction of carboxylic acids **7**–**12** with Boc-protected polyamines **5a**–**f** utilized coupling reagents EDC·HCl or EDC·HCl/HOBt or HBTU in anhydrous DMF/CH_2_Cl_2_ with the products then deprotected (2,2,2-trifluoroacetic acid (TFA) in CH_2_Cl_2_) to afford the target compounds as their di-TFA salts (Scheme 2, Figures S9–S44).

**Figure 5:**
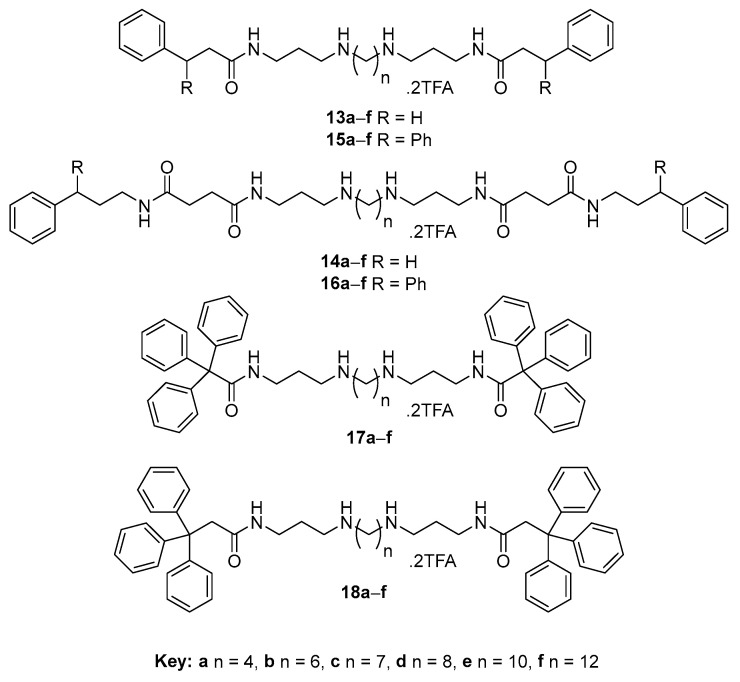
Diacylpolyamines **13a**–**f** to **18a**–**f**.

The structures of the synthesized diacylpolyamine library are shown in Figure 5.

## 2. Results and Discussion

The intrinsic antimicrobial activity of the series was evaluated against a range of Gram-positive (*S. aureus* and MRSA) and Gram-negative (*Escherichia coli*, *Pseudomonas aeruginosa*, *Klebsiella pneumoniae* and *Acinetobacter baumannii*) bacteria and two fungal strains (*Candida albicans* and *Cryptococcus neoformans*) (Table 1). Cytotoxicity towards HEK293 (human kidney epithelial cell line, IC_50_) and hemolytic activity against human red blood cells (HC_10_) were also determined (Table 2). The thiourea analogues **6a**–**f** uniformly exhibited strong growth inhibition of bacteria MRSA (MIC ≤ 0.34 μM) and *E. coli* (MIC < 2.8 μM) and fungus *C. albicans* (MIC ≤ 0.3 μM). The *S. aureus* and *E. coli* inhibition results for **6a** were consistent with previously reported data for the same compound, although, in our case, less pronounced activity was observed towards *P. aeruginosa* [10]. There was no apparent effect of polyamine chain length on antimicrobial activity. Variable levels of cytotoxicity and hemolytic properties were observed for **6a**–**f**, with the PA-3-10-3 analogue (**6e**) identified as having the most favorable (least toxic) profile with IC_50_ > 39.5 μM and HC_10_ > 39.5 μM. The observation of cytotoxicity/hemolytic properties prompted our investigation of a further set of analogues that explored replacement of the thiourea linking group present in **6a**–**f** with more simplified amide-based linkers. Further, 3-phenylpropionic acid (**13a**–**f**) and 3-phenylpropylamine-succinic acid (**14a**–**f**) -based linkers were almost universally devoid of antimicrobial properties except for the longer chain PA-3-10-3 and PA-3-12-3 variants, which exhibited weak (**13e**, **13f**, **14e**) to potent (**14f**) activity towards MRSA. When compounds in these sets were evaluated for detrimental cellular effects, none exhibited cytotoxicity or hemolytic properties, identifying PA-3-12-3 analogue **14f** as a non-toxic, non-hemolytic, strongly active anti-MRSA molecule. Increasing the lipophilicity by inclusion of an additional phenyl ring in the capping acid provided two sets of analogues (**15a**–**f**, **16a**–**f**) that exhibited enhanced anti-MRSA activity (all examples MIC ≤ 0.29 μM) and with some examples exhibiting activity towards the Gram-negative bacteria *E. coli* (**15a**–**f**, MIC ≤ 0.27 to 4.6 μM) and fungus *C. neoformans* (**15c**, **15f**, **16a**, **16e**, MIC ≤ 0.28 μM). Of these two compound sets, only the longer chain variants **15f**, **16e** and **16f,** exhibited cytotoxicity and/or hemolytic properties, identifying, in particular, the majority of the diphenylpropyl analogue set (**15a**–**e**) as being of further interest as antimicrobial agents.

The observation of increased cytotoxicity/hemolytic activity for the longer chain variants identified that toxicity, likely arising from enhanced penetration/disruption of mammalian membranes, was not solely dependent upon the lipophilicity of just the aromatic head group but was a function of the whole molecule. Calculated logP values (cLogP) were generated for free base structures using DataWarrior [32] and are included in Table 2. For the two sets of analogues bearing diphenyl groups at each end of the polyamine, the ‘second hydrophobicity threshold’ [33] appears to be in the order of cLogP 9–10. The concept of a second hydrophobicity threshold was proposed to explain the observed ability of cationic antimicrobial peptides (CAPs) to insert into and ultimately disrupt bacterial versus mammalian membranes, with higher hydrophobicity/lipophilicity associated with hemolytic activity in erythrocytes. A nuance to the hydrophobicity threshold model of mammalian cell toxicity in the current context was observed for the final two sets of analogues (**17a**–**f**, **18a**–**f**) bearing three phenyl ring-containing substituents at each end of the polyamine chain. Although both sets of analogues exhibited broad spectrum activity towards a range of microbes in the screening panel, including MRSA, *E. coli*, *A. baumannii*, *C. albicans* and *C. neoformans*, interest in these compounds was abrogated by their moderate to strong cytotoxic/hemolytic properties. The calculated LogP values for these last two sets of analogues (Table 2) covered the range of 7.8–12.4, with the lower values of 7.8–9.2 (**17a**–**c**, **18a**) being of similar magnitude to those calculated for non-cytotoxic/non-hemolytic diphenyl variants **15c**–**e** and **16c**–**d**, suggesting that, in the cases of **17a**–**f** and **18a**–**f**, the presence of the triphenyl aromatic head group was itself enough to cause mammalian toxicity.

**Figure 6:**
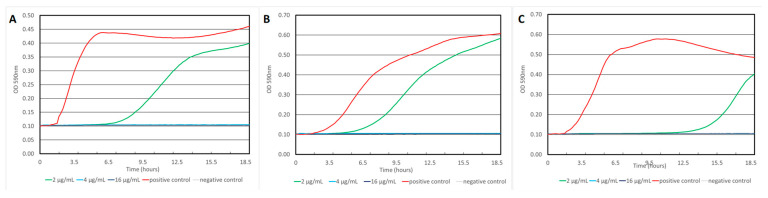
Bacterial growth inhibition exhibited by **15d** against (**A**) *S. aureus* ATCC 25923, (**B**) MRSA (CF-Marseille); (**C**) *Bacillus cereus* ATCC 11778 with different concentrations. Positive control was bacteria only and negative control was media only.

Analogue **15d** was chosen for closer examination of antibacterial activity and preliminary mechanism of action evaluation as it exhibited potent antibacterial properties with no detectable cytotoxicity or hemolytic activities. The kinetics of antibacterial activity of **15d** towards Gram-positive bacteria were undertaken by measuring real-time growth inhibition curves against *S. aureus* ATCC 25923, MRSA (CF-Marseille) [34] and *Bacillus cereus* ATCC 11778. The test compound completely inhibited all three strains at 4.4 μM (4 µg/mL) and 17 μM (16 µg/mL) concentration, whereas, at the lowest tested concentration, 2.2 μM (2 µg/mL), bacterial growth was detected after 6 h for *S. aureus*, 4 h for MRSA and 12 h for *B. cereus* (Figure 6). Classical microdilution methodology determined an MIC value of 4.4 μM (4 μg/mL) for **15d** towards these three microorganisms, with the values matching those observed at 18 h in the real-time growth inhibition curve plots. The same values were observed for the minimum bactericidal concentration (MBC) for **15d** against the three organisms, identifying this analogue as being bactericidal. 

**Figure 7:**
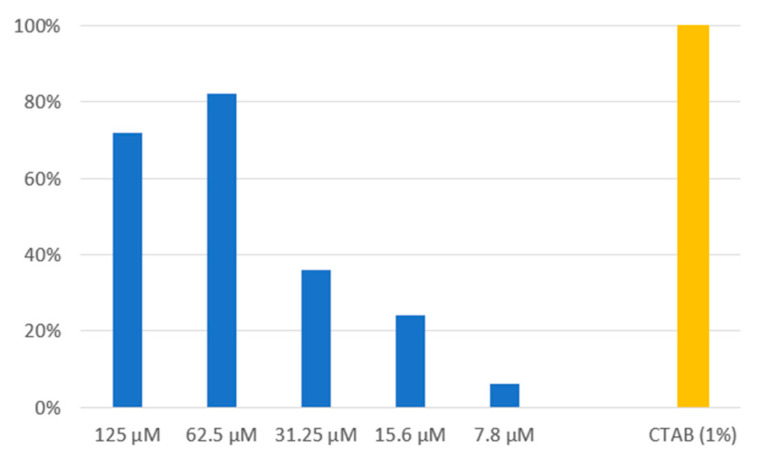
Dose-dependent ATP release from *S. aureus* ATCC 25923 exhibited by **15d**. CTAB (1%) is the positive control.

The mechanism of action of antibacterial activity observed for **15d** was attributed to the ability to disrupt the bacterial cell membrane. Brief (1 s) exposure of *S. aureus* ATCC 25923 cells to the test compound led to rapid dose-dependent leakage of intracellular ATP, as determined by a bioluminescence assay (Figure 7) [8]. The higher compound doses (62.5 to 125 μM) provided leakage comparable in magnitude to that observed for the positive control, a 1% solution of cationic detergent cetyltrimethylammonium bromide (CTAB).

The original report describing (bis)arylthioureido analogues **3** and **4** identified the latter as being synergistic with kanamycin, causing 8-fold reduction in MIC against *S. aureus* and *P. aeruginosa* [10]. No synergism was observed in combination with ampicillin or norfloxacin. We have evaluated the set of analogues for the ability to enhance the antibiotic activity of doxycycline against *P. aeruginosa* ATCC 27853 (Table 3). In this assay, a fixed concentration of doxycycline of 2 μg/mL (4.5 μM), which is twenty-fold lower than the intrinsic MIC [40 μg/mL (90 μM)] against this organism, is used, with each of the test compounds evaluated at a range of concentrations varying from 3.125 to 50–100 μM, with the upper concentration dependent upon compounds’ intrinsic MIC towards *P. aeruginosa*. All but the longest chain variant of the thiourea analogues exhibited antibiotic enhancement, with **6a**–**c** and **6e** being particularly strong enhancers. Of the remaining thirty-six compounds (**13a**–**f** to **18a**–**f**), only modest potency of antibiotic enhancement was observed, with **16a** (8-fold increase to 25 μM) and **15a** (4-fold increase to 12.5 μM) being the most active. Although disappointing, these results highlight that further research is required to fine-tune the attributes of the aromatic head groups and their linker unit of α,ω-disubstituted polyamines to develop non-toxic drug candidates that can enhance action of antibiotics towards drug-resistant bacteria. The current results have identified the diaryl head group series **15a**–**e** and **16a**–**d** as good starting points for further optimization as antimicrobial agents, details of which will be reported in due course.

## 3. Materials and Methods

### 3.1. Chemistry: General Methods

Infrared spectra were run as dry films on an ATR crystal and acquired with a Perkin-Elmer 100 Fourier transform infrared spectrometer equipped with a Universal ATR Sampling Accessory (Waltham, MA, USA). HRMS data were acquired on a Bruker micrOTOF QII spectrometer (Bruker Daltonics, Bremen, Germany). Melting points were obtained on an electrothermal melting point apparatus and are uncorrected. NMR spectra were recorded on a Bruker AVANCE AVIII 400 MHz spectrometer (Bruker, Karlsruhe, Germany) operating at 400.13 MHz for ^1^H nuclei and 100.62 MHz for ^13^C nuclei. Proto-deutero solvent signals were used as internal references (DMSO-*d*_6_: δ_H_ 2.50, δ_C_ 39.52; CD_3_OD: δ_H_ 3.31, δ_C_ 49.00). For ^1^H NMR, the data are quoted as position (δ), relative integral, multiplicity (s = singlet, d = doublet, t = triplet, q = quartet, m = multiplet, br = broad), coupling constant (*J*, Hz) and assignment to the atom. The ^13^C NMR data are quoted as position (δ) and assignment to the atom. All atom assignments established by interpretation of 2D NMR data. Flash column chromatography was carried out using either Davisil silica gel (40–60 μm) (Grace Scientific, MD, USA) or Merck LiChroprep RP-8 (40–63 µm) (Merck Millipore, Darmstadt, Germany). Thin layer chromatography was conducted on Merck DC-plastikfolien Kieselgel 60 F254 or Kieselgel 60 RP-18 F254S plates. All solvents used were of analytical grade or better and/or purified according to standard procedures. Chemical reagents used were purchased from standard chemical suppliers and used as purchased. All samples were determined to >95% purity. Protected polyamines di-*tert*-butyl butane-1,4-diylbis((3-aminopropyl)carbamate) (**5a**) di-*tert*-butyl hexane-1,6-diylbis((3-aminopropyl)carbamate) (**5b**), di-*tert*-butyl heptane-1,7-diylbis((3-aminopropyl)carbamate) (**5c**), di-*tert*-butyl octane-1,8-diylbis((3-aminopropyl)carbamate) (**5d**), di-*tert*-butyl decane-1,10-diylbis((3-aminopropyl)carbamate) (**5e**) and di-*tert*-butyl dodecane-1,12-diylbis((3-aminopropyl)carbamate) (**5f**) were synthesized by literature procedures [28,29,30,31].

### 3.2. Synthesis of Compounds

#### 3.2.1. General Procedure A: Reaction of Benzhydryl Isothiocyanate with Boc-Protected Polyamine

To a stirred solution of Boc-protected polyamine (**5a**–**f**) (1 equiv.) in anhydrous CH_2_Cl_2_ (5 mL) at 0 °C was added benzhydryl isothiocyanate (2.2 eq.) in CH_2_Cl_2_ (5 mL) dropwise. The reaction mixture was stirred for 18 h before the solvent was removed under reduced pressure and the crude product purified by silica gel column chromatography (hexane:EtOAc, 60:40 to 25:75).

#### 3.2.2. General Procedure B: Boc Deprotection Using 1% HCl/EtOAc

A solution of Boc-protected thiourea polyamine (20–50 mg) was dissolved in 1% HCl in EtOAc (5–12.5 mL) and stirred at rt under N_2_ for 12 h. Additional EtOAc (2 × 20 mL) was added and the mixture stirred for 15 min before the liquid was decanted and the solid product dried under reduced pressure.

#### 3.2.3. General Procedure C: Amide Bond Formation

To a stirred solution of EDC·HCl (2.6 eq.) or EDC·HCl and HOBt (2.6 eq.) or HBTU (2.5 eq.), carboxylic acid or amido acid starting material (2.2 eq.) and DIPEA (6 eq.) or DMAP (2 eq.) incubated for 30 min in anhydrous DMF/CH_2_Cl_2_ (2 mL) was added Boc protected polyamine (**5a**–**f**) (1 eq.) at 0 °C. The reaction mixture was stirred under N_2_ atmosphere for 18 h, then added CH_2_Cl_2_ (50 mL) and washed with salt. NaHCO_3_ (1 × 100 mL) and water (5 × 100 mL) then dried with anhydrous MgSO_4_. The organic layer was then dried under reduced pressure before purification by silica gel column chromatography (CH_2_Cl_2_:MeOH, 97:3→85:15).

#### 3.2.4. General Procedure D: Boc Deprotection Using TFA/Dichloromethane

A solution of *tert*-butyl-carboxylate derivative in anhydrous CH_2_Cl_2_ (2 mL) and TFA (0.2 mL) was stirred at room temperature under N_2_ for 2h, then dried under reduced pressure. The crude product was purified using C_8_ reversed-phase column chromatography (MeOH (+0.05% TFA):H_2_O (+0.05% TFA), 0:100→3:1).

##### *N*^1^,*N*^4^-Bis(3-(3-benzhydrylthioureido)propyl)butane-1,4-diaminium chloride (**6a**)

Following general procedure A, reaction of di-*tert*-butyl butane 1,4-diylbis((3-aminopropyl)carbamate) (**5a**) (100 mg, 0.25 mmol) and benzhydryl isothiocyanate (118 mg, 0.52 mmol) afforded di-*tert*-butyl butane-1,4-diylbis((3-(3-benzhydrylthioureido)propyl)carbamate) as a white solid (116 mg, 55%). Following general procedure B, a sub-sample of this material (20 mg, 0.023 mmol) was reacted with 1M HCl in EtOAc (5 mL) to afford the dihydrochloride salt **6a** as a white solid (14 mg, 84%). R*_f_* = 0.46 (RP-18, MeOH:10% aq. HCl, 5:1); m.p. 168–169 °C; IR (ATR) *v*_max_ 3399, 3228, 3070, 2924, 2851, 2778, 2427, 1585, 1560, 1452, 740 cm^−1^; ^1^H NMR (DMSO-*d*_6_, 400 MHz) δ 9.05–8.86 (6H, m, NH-2, NH_2_-12), 8.35–8.22 (2H, m, NH-8), 7.38–7.26 (16H, m, H-5, H-6), 7.26–7.18 (4H, m, H-7), 6.73–6.66 (2H, m, H-3), 3.58–3.47 (4H, m, H_2_-9), 2.98–2.80 (8H, m, H_2_-11, H_2_-13), 1.94–1.81 (4H, m, H_2_-10), 1.73–1.62 (4H, m, H_2_-14); ^13^C NMR (DMSO-*d*_6_, 100 MHz) δ 182.6 (C-1), 142.7 (C-4), 128.4, 127.2 (C-5, C-6), 126.9 (C-7), 60.6 (C-3), 45.9 (C-13), 44.5 (C-11), 40.7 (C-9), 25.7 (C-10), 22.6 (C-14); (+)-HRESIMS [M+H]^+^ *m*/*z* 653.3441 (calcd for C_38_H_49_N_6_S_2_, 653.3455).

##### *N*^1^,*N*^6^-Bis(3-(3-benzhydrylthioureido)propyl)hexane-1,6-diaminium chloride (**6b**)

Following general procedure A, reaction of di-*tert*-butyl hexane-1,6-diylbis((3-aminopropyl)carbamate) (**5b**) (50 mg, 0.12 mmol) and benzhydryl isothiocyanate (55 mg, 0.24 mmol) afforded di-*tert*-butyl hexane-1,6-diylbis((3-(3-benzhydrylthioureido)propyl)carbamate) as a white solid (69 mg, 67%). Following general procedure B, a sub-sample of this material (20 mg, 0.023 mmol) was reacted with 1M HCl in EtOAc (5 mL) to afford the dihydrochloride salt **6b** as a white solid (16 mg, 92%). R*_f_* = 0.43 (RP-18, MeOH:10% aq. HCl, 5:1); m.p. 122–123.5 °C; IR (ATR) *v*_max_ 3377, 3248, 3059, 3027, 2937, 2852, 2785, 1586, 1543, 1451, 1344, 744 cm^−1^; ^1^H NMR (DMSO-*d*_6_, 400 MHz) δ 8.99–8.86 (2H, m, NH-2), 8.86–8.76 (4H, m, NH_2_-12), 8.29–8.20 (2H, m, NH-8), 7.36–7.26 (16H, m, H-5, H-6), 7.26–7.19 (4H, m, H-7), 6.76–6.67 (2H, m, H-3), 3.51 (4H, dt, *J* = 6.3, 5.8 Hz, H_2_-9), 2.95–2.86 (4H, m, H_2_-11), 2.86–2.78 (4H, m, H_2_-13), 1.86 (4H, tt, *J* = 7.3, 7.0 Hz, H_2_-10), 1.66–1.53 (4H, m, H_2_-14), 1.35–1.26 (4H, m, H_2_-15); ^13^C NMR (DMSO-*d*_6_, 100 MHz) δ 182.5 (C-1), 142.7 (C-4), 128.4, 127.2 (C-5, C-6), 126.9 (C-7), 60.6 (C-3), 46.5 (C-13), 44.5 (C-11), 40.7 (C-9), 25.7 (C-10), 25.4, 25.1 (C-14, C-15); (+)-HRESIMS [M+H]^+^ *m*/*z* 681.3744 (calcd for C_40_H_53_N_6_S_2_, 681.3768).

##### *N*^1^,*N*^7^-Bis(3-(3-benzhydrylthioureido)propyl)heptane-1,7-diaminium chloride (**6c**)

Following general procedure A, reaction of di-*tert*-butyl heptane-1,7-diylbis((3-aminopropyl)carbamate) (**5c**) (50 mg, 0.11 mmol) and benzhydryl isothiocyanate (53 mg, 0.24 mmol) afforded di-*tert*-butyl heptane-1,7-diylbis((3-(3-benzhydrylthioureido)propyl)carbamate) as a white solid (87 mg, 87%). Following general procedure B, a sub-sample of this material (20 mg, 0.022 mmol) was reacted with 1M HCl in EtOAc (5 mL) to afford the dihydrochloride salt **6c** as a white solid (15 mg, 89%). R*_f_* = 0.37 (RP-18, MeOH:10% aq. HCl, 5:1); m.p. 115–116 °C; IR (ATR) *v*_max_ 3360, 3247, 3058, 2933, 2853, 2782, 2445, 1586, 1542, 1450, 743 cm^−1^; ^1^H NMR (DMSO-*d*_6_, 400 MHz) δ 8.87–8.76 (2H, m, NH-2), 8.76–8.66 (4H, m, NH_2_-12), 8.12 (2H, t, *J* = 5.5 Hz, NH-8), 7.36–7.26 (16H, m, H-5, H-6), 7.26–7.21 (4H, m, H-7), 6.76–6.66 (2H, m, H-3), 3.51 (4H, dt, *J* = 6.3, 5.5 Hz, H_2_-9), 2.95–2.86 (4H, m, H_2_-11), 2.86–2.79 (4H, m, H_2_-13), 1.85 (4H, tt, *J* = 7.4, 7.2 Hz, H_2_-10), 1.64–1.54 (4H, m, H_2_-14), 1.34–1.23 (6H, m, H_2_-15, H_2_-16); ^13^C NMR (DMSO-*d*_6_, 100 MHz) δ 182.4 (C-1), 142.6 (C-4), 128.4, 127.2 (C-5, C-6), 126.9 (C-7), 60.6 (C-3), 46.6 (C-13), 44.5 (C-11), 40.7 (C-9), 27.9 (C-15 or C-16), 25.7, 25.7, 25.3 (C-10, C-14, C-15 or C-16); (+)-HRESIMS [M+H]^+^ *m*/*z* 695.3898 (calcd for C_41_H_55_N_6_S_2_, 695.3924)

##### *N*^1^,*N*^8^-Bis(3-(3-benzhydrylthioureido)propyl)octane-1,8-diaminium chloride (**6d**)

Following general procedure A, reaction of di-*tert*-butyl octane-1,8-diylbis((3-aminopropyl)carbamate) (**5d**) (50 mg, 0.11 mmol) and benzhydryl isothiocyanate (51 mg, 0.23 mmol) afforded di-*tert*-butyl octane-1,8-diylbis((3-(3-benzhydrylthioureido)propyl)carbamate) as a white solid (68 mg, 69%). Following general procedure B, a sub-sample of this material (50 mg, 0.055 mmol) was reacted with 1M HCl in EtOAc (12.5 mL) to afford the dihydrochloride salt **6d** as a white solid (38 mg, 88%). R*_f_* = 0.34 (RP-18, MeOH:10% aq. HCl, 5:1); m.p. 113–115 °C; IR (ATR) *v*_max_ 3240, 3063, 2932, 2854, 2777, 1543, 1493, 1449, 1343, 742 cm^−1^; ^1^H NMR (DMSO-*d*_6_, 400 MHz) δ 8.85–8.73 (2H, m, NH-2), 8.73–8.61 (4H, m, NH_2_-12), 8.09 (2H, t, *J* = 5.5 Hz, NH-8), 7.40–7.26 (16H, m, H-5, H-6), 7.26–7.21 (4H, m, H-7), 6.75–6.66 (2H, m, H-3), 3.51 (4H, dt, *J* = 6.3, 5.9 Hz, H_2_-9), 2.94–2.86 (4H, m, H_2_-11), 2.86–2.78 (4H, m, H_2_-13), 1.85 (4H, tt, *J* = 7.4, 6.9 Hz, H_2_-10), 1.64–1.53 (4H, m, H_2_-14), 1.34–1.22 (8H, m, H_2_-15, H_2_-16); ^13^C NMR (DMSO-*d*_6_, 100 MHz) δ 182.5 (C-1), 142.6 (C-4), 128.4, 127.2 (C-5, C-6), 126.9 (C-7), 60.6 (C-3), 46.7 (C-13), 44.5 (C-11), 40.7 (C-9), 28.2 (C-15 or C-16), 25.8, 25.7, 25.3 (C-10, C-14, C-15 or C-16); (+)-HRESIMS [M+H]^+^ *m*/*z* 709.4067 (calcd for C_42_H_57_N_6_S_2_, 709.4081).

##### *N*^1^,*N*^10^-Bis(3-(3-benzhydrylthioureido)propyl)decane-1,10-diaminium chloride (**6e**)

Following general procedure A, reaction of di-*tert*-butyl decane-1,10-diylbis((3-aminopropyl)carbamate) (**5e**) (50 mg, 0.10 mmol) and benzhydryl isothiocyanate (51 mg, 0.23 mmol) afforded di-*tert*-butyl decane-1,10-diylbis((3-(3-benzhydrylthioureido)propyl)carbamate) as a white solid (59 mg, 61%). Following general procedure B, a sub-sample of this material (20 mg, 0.021 mmol) was reacted with 1M HCl in EtOAc (5 mL) to afford the dihydrochloride salt **6e** as a white solid (16 mg. 94%). R*_f_* = 0.26 (RP-18, MeOH:10% aq. HCl, 5:1); m.p. 114–115 °C; IR (ATR) *v*_max_ 3390, 3251, 3060, 2928, 2853, 2782, 2429, 1586, 1542, 1452, 1343, 743 cm^−1^; ^1^H NMR (DMSO-*d*_6_, 400 MHz) δ 8.93–8.78 (2H, m, NH-2), 8.78–8.67 (4H, m, NH_2_-12), 8.17 (2H, t, *J* = 5.5 Hz, NH-8), 7.35–7.26 (16H, m, H-5, H-6), 7.26–7.20 (4H, m, H-7), 6.75–6.66 (2H, m, H-3), 3.51 (4H, dt, *J* = 6.4, 6.2 Hz, H_2_-9), 2.94–2.85 (4H, m, H_2_-11), 2.85–2.77 (4H, m, H_2_-13), 1.85 (4H, tt, *J* = 7.4, 7.4 Hz, H_2_-10), 1.64–1.53 (4H, m, H_2_-14), 1.33–1.22 (12H, m, H_2_-15, H_2_-16, H_2_-17); ^13^C NMR (DMSO-*d*_6_, 100 MHz) δ 182.4 (C-1), 142.6 (C-4), 128.4, 127.2 (C-5, C-6), 126.9 (C-7), 60.6 (C-3), 46.7 (C-13), 44.5 (C-11), 40.7 (C-9), 28.6, 28.4 (C-15 or C-16 or C-17), 25.9, 25.7, 25.4 (C-10, C-14, C-15 or C-16 or C-17); (+)-HRESIMS [M+H]^+^ *m*/*z* 737.4364 (calcd for C_44_H_61_N_6_S_2_, 737.4394).

##### *N*^1^,*N*^12^-Bis(3-(3-benzhydrylthioureido)propyl)dodecane-1,12-diaminium chloride (**6f**)

Following general procedure A, reaction of di-*tert*-butyl dodecane-1,12-diylbis((3-aminopropyl)carbamate) (**5f**) (50 mg, 0.10 mmol) and benzhydryl isothiocyanate (46 mg, 0.20 mmol) afforded di-*tert*-butyl dodecane-1,12-diylbis((3-(3-benzhydrylthioureido)propyl)carbamate) as a white solid (64 mg, 68%). Following general procedure B, a sub-sample of this material (20 mg, 0.021 mmol) was reacted with 1M HCl in EtOAc (5 mL) to afford the dihydrochloride salt **6f** as a white solid (16 mg, 91%). R*_f_* = 0.17 (RP-18, MeOH:10% aq. HCl, 5:1); m.p. 103–104 °C; IR (ATR) *v*_max_ 3370, 3261, 3064, 3029, 2925, 2852, 2781, 1644, 1542, 1450, 1342, 743 cm^−1^; ^1^H NMR (DMSO-*d*_6_, 400 MHz) δ 8.92–8.80 (2H, m, NH-2), 8.80–8.67 (4H, m, NH_2_-12), 8.18 (2H, t, *J* = 5.4 Hz, NH-8), 7.36–7.26 (16H, m, H-5, H-6), 7.26–7.20 (4H, m, H-7), 6.76–6.66 (2H, m, H-3), 3.50 (4H, dt, *J* = 6.0, 5.8 Hz, H_2_-9), 2.94–2.85 (4H, m, H_2_-11), 2.85–2.77 (4H, m, H_2_-13), 1.85 (4H, tt, *J* = 7.4, 7.2 Hz, H_2_-10), 1.64–1.52 (4H, m, H_2_-14), 1.32–1.21 (16H, m, H_2_-15, H_2_-16, H_2_-17, H_2_-18); ^13^C NMR (DMSO-*d*_6_, 100 MHz) δ 182.7 (C-1), 142.7 (C-4), 128.4, 127.2 (C-5, C-6), 126.9 (C-7), 60.6 (C-3), 46.7 (C-13), 44.5 (C-11), 40.7 (C-9), 28.9, 28.8, 28.5 (C-15 or C-16 or C-17 or C-18), 25.9, 25.7, 25.4 (C-10, C-14, C-15 or C-16 or C-17 or C-18); (+)-HRESIMS [M+H]^+^ *m*/*z* 765.4680 (calcd for C_46_H_65_N_6_S_2_, 765.4707).

##### 4-Oxo-4-((3-phenylpropyl)amino)butanoic acid (**8**)

3-Phenylpropylamine (0.63 mL, 4.4 mmol) and succinic anhydride (440 mg, 4.4 mmol) were stirred in dry CH_2_Cl_2_ (5 mL) for 9 h under an N_2_ atmosphere. The solvent was then removed under reduced pressure and the product washed with cold CH_2_Cl_2_ (20 mL) and water (50 mL) to afford **8** as a white solid (432 mg, 1043 mg theory, 41%). R*_f_* = 0.29 (SiO_2_, 10% MeOH/CH_2_Cl_2_); m.p 94–95 °C; IR (ATR) *v*_max_ 3301, 3030, 2932, 2855, 1805, 1688, 1549 cm^−1^; ^1^H NMR (DMSO-*d_6_*, 400 MHz) δ 12.03 (1H, br s, OH), 7.85 (1H, t, *J* = 5.4 Hz, NH-5), 7.29–7.25 (2H, m, H-11), 7.21–7.18 (2H, m, H-10), 7.18–7.15 (1H, m, H-12), 3.04 (2H, dt, *J* = 6.6, 5.4 Hz, H_2_-6), 2.56 (2H, t, *J* = 7.7 Hz, H_2_-8), 2.42 (2H, t, *J* = 6.8 Hz, H_2_-2), 2.30 (2H, t, *J* = 6.8 Hz, H_2_-3), 1.71–1.64 (2H, m, H_2_-7); ^13^C NMR (DMSO-*d_6_*, 100 MHz) δ 173.8 (C-1), 170.8 (C-4), 141.8 (C-9), 128.3, 128.2 (C-10, C-11), 125.7 (C-12), 38.1 (C-6), 32.5 (C-8), 31.0 (C-7), 30.0 (C-3), 29.2 (C-2); (+)-HRESIMS [M+Na]^+^ *m*/*z* 236.1284 (calcd for C_13_H_17_NNaO_3_, 258.1101).

##### 4-((3,3-Diphenylpropyl)amino)-4-oxobutanoic acid (**10**)

To a stirred solution of succinic anhydride (100 mg, 1 mmol) in anhydrous CH_2_Cl_2_ (15 mL) was added 3,3-diphenylpropylamine (211 mg, 1 mmol) under N_2_ atmosphere at room temperature. The mixture was stirred for 18 h and solvent removed under reduced pressure before washing with 1% aq. HCl (1 × 100 mL) to afford **10** as a white solid (302 mg, 97%). R*_f_* = 0.35 (SiO_2_, 10% MeOH/CH_2_Cl_2_); m.p. 134–137°C; IR (ATR) *v*_max_ 3547, 3390, 3361, 3024, 2941, 1716, 1624, 1554, 1173, 747, 695 cm^−1^; ^1^H NMR (DMSO-*d_6_*, 400 MHz) δ 12.07 (1H, br s, OH), 7.85 (1H, t, *J* = 5.3 Hz, NH-5), 7.31–7.25 (8H, m, H-10, H-11), 7.18–7.14 (2H, m, H-12), 3.98 (1H, t, *J* = 7.8 Hz, H-8), 2.93 (2H, dt, *J* = 7.8, 6.9, H_2_-6), 2.40 (2H, t, *J* = 6.6 Hz, H_2_-2 or H_2_-3), 2.28 (2H, t, *J* = 7.0 Hz, H_2_-2 or H_2_-3), 2.14 (2H, dt, *J* = 7.8, 6.9 Hz, H_2_-7); ^13^C NMR (DMSO-*d_6_*, 100 MHz) δ 173.8 (C-1), 170.8 (C-4), 144.8 (C-9), 128.4, 127.6 (C-10, C-11), 126.0 (C-12), 47.8 (C-8), 37.3 (C-6), 34.6 (C-7), 30.0, 29.1 (C-2, C-3); (+)-HRESIMS [M+Na]^+^ *m*/*z* 334.1413 (calcd for C_19_H_21_NNaO_3_, 334.1414).

##### *N*^1^,*N*^4^-Bis(3-(3-phenylpropanamido)propyl)butane-1,4-diaminium 2,2,2-trifluoroacetate (**13a**)

Following general procedure C, reaction of 3-phenylpropanoic acid (**7**) (82 mg, 0.55 mmol) with di-*tert*-butyl butane-1,4-diylbis((3-aminopropyl)carbamate) (**5a**) (100 mg, 0.25 mmol), EDC·HCl (124 mg, 0.65 mmol), HOBt (87 mg, 0.65 mmol) and DIPEA (0.26 mL, 1.5 mmol) afforded di-*tert*-butyl butane-1,4-diylbis((3-(3-phenylpropanamido) propyl)carbamate) as a colorless oil (72 mg, 43%). Following general procedure D, a sub-sample of this material (41 mg, 0.061 mmol) was reacted with TFA (0.2 mL) in CH_2_Cl_2_ (2 mL). Purification was achieved by C_8_ reversed-phase column chromatography (MeOH (+0.05% TFA):H_2_O (+0.05% TFA), 0:100→3:1) to afford the di-TFA salt **13a** as a colorless oil (39 mg, 91%). R*_f_* = 0.54 (RP-18, MeOH:10% aq. HCl, 7:3); IR (ATR) *v*_max_ 3327, 2948, 2835, 1653, 1450, 1412, 1112, 1017 cm^−1^; ^1^H NMR (CD_3_OD, 400 MHz) δ 7.30–7.16 (10H, m, H-5, H-6, H-7), 3.25 (4H, t, *J* = 6.3 Hz, H_2_-9), 2.93 (8H, t, *J* = 7.4 Hz, H_2_-3, H_2_-13), 2.80 (4H, t, *J* = 7.1 Hz, H_2_-11), 2.56 (4H, t, *J* = 7.5 Hz, H_2_-2), 1.83–1.75 (8H, m, H_2_-10, H_2_-14); ^13^C NMR (CD_3_OD, 100 MHz) δ 176.4 (C-1), 141.9 (C-4), 129.5, 129.4 (C-5, C-6), 127.3 (C-7), 48.1 (C-13), 46.0 (C-11), 38.3 (C-2), 36.6 (C-9), 32.6 (C-3), 27.6 (C-10), 24.3 (C-14); (+)-HRESIMS [M+H]^+^ *m*/*z* 467.3380 (calcd for C_28_H_43_N_4_O_2_, 467.3381).

##### *N*^1^,*N*^6^-Bis(3-(3-phenylpropanamido)propyl)hexane-1,6-diaminium 2,2,2-trifluoroacetate (**13b**)

Following general procedure C, 3-phenylpropanoic acid (**7**) (38.4 mg, 0.26 mmol) with di-*tert*-butyl hexane-1,6-diylbis((3-aminopropyl)carbamate) (**5b**) (50 mg, 0.12 mmol), EDC·HCl (57.9 mg, 0.30 mmol), HOBt (40.8 mg, 0.30 mmol) and DIPEA (0.12 mL, 0.69 mmol) afforded di-*tert*-butyl hexane-1,6-diylbis((3-(3-phenylpropanamido)propyl)carbamate) as a colorless oil (48 mg, 58%). Following general procedure D, a sub-sample of this material (27 mg, 0.039 mmol) was reacted with TFA (0.2 mL) in CH_2_Cl_2_ (2 mL). Purification was achieved by C_8_ reversed-phase column chromatography (MeOH (+0.05% TFA):H_2_O (+0.05% TFA), 0:100→3:1) to afford the di-TFA salt **13b** as a colorless gum (27 mg, 96%). R*_f_* = 0.53 (RP-18, MeOH:10% aq. HCl, 7:3); IR (ATR) *v*_max_ 3288, 3029, 2929, 2857, 1671, 1646, 1556, 1497, 1456, 1199, 1176, 1128, 1078, 1020, 833, 799, 750, 720 cm^−1^; ^1^H NMR (CD_3_OD, 400 MHz) δ 7.30–7.15 (10H, m, H-5, H-6, H-7), 3.24 (4H, t, *J* = 6.4 Hz, H_2_-9), 2.93 (4H, t, *J* = 7.4 Hz, H_2_-3), 2.89 (4H, t, *J* = 7.4 Hz, H_2_-13), 2.77 (4H, t, *J* = 7.1 Hz, H_2_-11), 2.55 (4H, t, *J* = 7.5 Hz, H_2_-2), 1.78 (4H, tt, *J* = 7.5, 7.5 Hz, H_2_-10), 1.74–1.66 (4H, m, H_2_-14) 1.50–1.43 (4H, m, H_2_-15); ^13^C NMR (CD_3_OD, 100 MHz) δ 176.3 (C-1), 141.9 (C-4), 129.5, 129.4 (C-5, C-6), 127.3 (C-7), 49.0 (C-13), 46.0 (C-11), 38.3 (C-2), 36.6 (C-9), 32.6 (C-3), 27.5 (C-10), 27.0 (C-15), 26.9 (C-14); (+)-HRESIMS [M+H]^+^ *m*/*z* 495.3679 (calcd for C_30_H_47_N_4_O_2_, 495.3694).

##### *N*^1^,*N*^7^-Bis(3-(3-phenylpropanamido)propyl)heptane-1,7-diaminium 2,2,2-trifluoroacetate (**13c**)

Following general procedure C, reaction of 3-phenylpropanoic acid (**7**) (37.2 mg, 0.25 mmol) with di-*tert*-butyl heptane-1,7-diylbis((3-aminopropyl)carbamate) (**5c**) (50 mg, 0.11 mmol), EDC·HCl (56.1 mg, 0.29 mmol), HOBt (39.6 mg, 0.29 mmol) and DIPEA (0.118 mL, 0.68 mmol) afforded di-*tert*-butyl heptane-1,7-diylbis((3-(3-phenylpropanamido)propyl)carbamate) as colorless oil (17 mg, 22%). Following general procedure D, a sub-sample of this material (9 mg, 0.013 mmol) was reacted with TFA (0.2 mL) in CH_2_Cl_2_ (2 mL). Purification was achieved by C_8_ reversed-phase column chromatography (MeOH (+0.05% TFA):H_2_O (+0.05% TFA), 0:100→3:1) to afford the di-TFA salt **13c** as a colorless gum (8 mg, 85%). R*_f_* = 0.53 (RP-18, MeOH:10% aq. HCl, 7:3); IR (ATR) *v*_max_ 3288, 2939, 1675, 1556, 1456, 1202, 1133, 834, 800, 702, 721 cm^−1^; ^1^H NMR (CD_3_OD, 400 MHz) δ 7.30–7.16 (10H, m, H-5, H-6, H-7), 3.24 (4H, t, *J* = 6.5 Hz, H_2_-9), 2.93 (4H, t, *J* = 7.4 Hz, H_2_-3), 2.87 (4H, t, *J* = 7.4 Hz, H_2_-13), 2.77 (4H, t, *J* = 7.1 Hz, H_2_-11), 2.55 (4H, t, *J* = 7.5 Hz, H_2_-2), 1.77 (4H, tt, *J* = 6.6, 6.6 Hz, H_2_-10), 1.73–1.64 (4H, m, H_2_-14), 1.47–1.42 (6H, m, H_2_-15, H_2_-16); ^13^C NMR (CD_3_OD, 100 MHz) δ 176.3 (C-1), 142.0 (C-4), 129.5, 129.4 (C-5, C-6), 127.3 (C-7), 48.9 (C-13), 46.0 (C-11), 38.3 (C-2), 36.6 (C-9), 32.6 (C-3), 29.7 (C-16), 27.6 (C10), 27.3, 27.2 (C-14, C-15); (+)-HRESIMS [M+H]^+^ *m*/*z* 509.3835 (calcd for C_31_H_49_N_4_O_2_, 509.3850).

##### *N*^1^,*N*^8^-Bis(3-(3-phenylpropanamido)propyl)octane-1,8-diaminium 2,2,2-trifluoroacetate (**13d**)

Following general procedure C, reaction of 3-phenylpropanoic acid (**7**) (36 mg, 0.24 mmol) with di-*tert*-butyl octane-1,8-diylbis((3-aminopropyl)carbamate) (**5d**) (50 mg, 0.11 mmol), EDC·HCl (54 mg, 0.28 mmol), HOBt (38 mg, 0.28 mmol) and DIPEA (0.114 mL, 0.65 mmol) afforded di-*tert*-butyl octane-1,8-diylbis((3-(3-phenylpropanamido)propyl)carbamate) as a colorless oil (44 mg, 55%). Following general procedure D, a sub-sample of this material (34 mg, 0.047 mmol) was reacted with TFA (0.2 mL) in CH_2_Cl_2_ (2 mL). Purification was achieved by C_8_ reversed-phase column chromatography (MeOH (+0.05% TFA):H_2_O (+0.05% TFA), 0:100→3:1) to afford the di-TFA salt **13d** as a pale yellow gum (32 mg, 91%). R*_f_* = 0.53 (RP-18, MeOH:10% aq. HCl, 7:3); IR (ATR) *v*_max_ 3288, 2929, 2858, 1672, 1556, 1497, 1456, 1200, 1177, 1131, 834, 800, 150, 721 cm^−1^; ^1^H NMR (CD_3_OD, 400 MHz) δ 7.30–7.15 (10H, m, H-5, H-6, H-7), 3.24 (4H, t, *J* = 6.4 Hz, H_2_-9), 2.93 (4H, t, *J* = 7.4 Hz, H_2_-3), 2.87 (4H, t, *J* = 7.4 Hz, H_2_-13), 2.76 (4H, t, *J* = 7.1 Hz, H_2_-11), 2.55 (4H, t, *J* = 7.5 Hz, H_2_-3), 1.77 (4H, tt, *J* = 6.6, 6.6 Hz, H_2_-10), 1.71–1.63 (4H, m, H_2_-14), 1.45–1.39 (8H, m, H_2_-15, H_2_-16); ^13^C NMR (CD_3_OD, 100 MHz) δ 176.3 (C-1), 142.0 (C-4), 129.5, 129.4 (C-5, C-6), 127.3 (C-7), 49.0 (C-13), 46.0 (C-11), 38.3 (C-2), 36.7 (C-9), 32.6 (C-3), 29.9 (C-16), 27.5, 27.4, 27.2 (C-10, C-14, C-15); (+)-HRESIMS [M+H]^+^ *m*/*z* 523.4008 (calcd for C_32_H_51_N_4_O_2_, 523.4007).

##### *N*^1^,*N*^10^-Bis(3-(3-phenylpropanamido)propyl)decane-1,10-diaminium 2,2,2-trifluoroacetate (**13e**)

Following general procedure C, reaction of 3-phenylpropanoic (**7**) (68 mg, 0.45 mmol) with di-*tert*-butyl decane-1,10-diylbis((3-aminopropyl)carbamate) (**5e**) (100 mg, 0.21 mmol), EDC·HCl (103 mg, 0.54 mmol), HOBt (72 mg, 0.53 mmol) and DIPEA (0.22 mL, 1.2 mmol) afforded di-*tert*-butyl decane-1,10-diylbis((3-(3-phenylpropanamido)propyl) carbamate) as a colorless oil (103 mg, 65%). Following general procedure D, a sub-sample of this material (79 mg, 0.11 mmol) was reacted with TFA (0.2 mL) in CH_2_Cl_2_ (2 mL). Purification was achieved by C_8_ reversed-phase column chromatography (MeOH (+0.05% TFA):H_2_O (+0.05% TFA), 0:100→3:1) to afford the di-TFA salt **13e** (83 mg, 97%) as a colorless oil. R*_f_* = 0.29 (RP-18, MeOH:10% aq. HCl, 7:3); IR (ATR) *v*_max_ 3308, 2944, 2832, 1683, 1450, 1114, 1022 cm^−1^; ^1^H NMR (CD_3_OD, 400 MHz) δ 7.30–7.15 (10H, m, H-5, H-6, H-7), 3.24 (4H, t, *J* = 6.5 Hz, H_2_-9), 2.93 (4H, t, *J* = 7.5 Hz, H_2_-3), 2.86 (4H, t, *J* = 7.8 Hz, H_2_-13), 2.75 (4H, t, *J* = 7.2 Hz, H_2_-11), 2.55 (4H, t, *J* = 7.5 Hz, H_2_-2), 1.76 (4H, tt, *J* = 6.7, 6.7 Hz, H_2_-10), 1.70–1.61 (4H, m, H_2_-14), 1.43–1.36 (12H, br s, H_2_-15, H_2_-16, H_2_-17); ^13^C NMR (CD_3_OD, 100 MHz) δ 176.4 (C-1), 142.0 (C-4), 129.5, 129.4 (C-5, C-6), 127.3 (C-7), 49.0 (C-13), 46.0 (C-11), 38.2 (C-2), 36.6 (C-9), 32.6 (C-3), 30.4, 30.2 (C-16, C-17), 27.6, 27.5, 27.3 (C-10, C-14, C-15); (+)-HRESIMS [M+H]^+^ *m*/*z* 551.4314 (calcd for C_34_H_55_N_4_O_2_, 551.4320).

##### *N*^1^,*N*^12^-Bis(3-(3-phenylpropanamido)propyl)dodecane-1,12-diaminium 2,2,2-trifluoroacetate (**13f**)

Following general procedure C, reaction of 3-phenylpropanoic (**7**) (48 mg, 0.32 mmol) with di-*tert*-butyl dodecane-1,12-diylbis((3-aminopropyl)carbamate) (**5f**) (75 mg, 0.15 mmol), EDC·HCl (73 mg, 0.38 mmol), HOBt (51 mg, 0.38 mmol) and DIPEA (0.15 mL, 0.86 mmol) afforded di-*tert*-butyl dodecane-1,12-diylbis((3-(3-phenylpropanamido) propyl)carbamate) as a colorless oil (45 mg, 39%). Following general procedure D, a sub-sample of this material (26 mg, 0.033 mmol) was reacted with TFA (0.2 mL) in CH_2_Cl_2_ (2 mL). Purification was achieved by C_8_ reversed-phase column chromatography (MeOH (+0.05% TFA):H_2_O (+0.05% TFA), 0:100→3:1) to afford the di-TFA salt **13f** (26.6 mg, 100%) as a colorless oil. ^1^H NMR (CD_3_OD, 400 MHz) δ 7.30–7.15 (10H, m, H-5, H-6, H-7), 3.24 (4H, t, *J* = 6.5 Hz, H_2_-9), 2.93 (4H, t, *J* = 7.5 Hz, H_2_-3), 2.85 (4H, t, *J* = 7.8 Hz, H_2_-13), 2.75 (4H, t, *J* = 7.1 Hz, H_2_-11), 2.55 (4H, t, *J* = 7.5 Hz, H_2_-2), 1.76 (4H, tt, *J* = 6.7, 6.7 Hz, H_2_-10), 1.70–1.61 (4H, m, H_2_-14), 1.43–1.32 (16H, br s, H_2_-15, H_2_-16, H_2_-17, H_2_-18); ^13^C NMR (CD_3_OD, 100 MHz) δ 176.4 (C-1), 142.0 (C-4), 129.5, 129.4 (C-5, C-6), 127.3 (C-7), 49.0 (C-13), 46.0 (C-11), 38.2 (C-2), 36.6 (C-9), 32.6 (C-3), 30.6, 30.5, 30.2 (C-16, C-17, C-18), 27.6, 27.5, 27.3 (C-10, C-14, C-15); (+)-HRESIMS [M+Na]^+^ *m*/*z* 601.4453 (calcd for C_36_H_59_NaN_4_O_2_, 601.4452). The NMR data agreed with literature [35].

##### *N*^1^,*N*^4^-Bis(3-(4-oxo-4-((3-phenylpropyl)amino)butanamido)propyl)butane-1,4-diaminium 2,2,2-trifluoroacetate (**14a**)

Following general procedure C, reaction of 4-oxo-4-((3-phenylpropyl)amino)butanoic acid **8** (129.3 mg, 0.55 mmol) with di-*tert*-butyl butane 1,4-diylbis((3-aminopropyl)carbamate) (**5a**) (100 mg, 0.25 mmol), HBTU (237 mg, 0.63 mmol) and DIPEA (0.26 mL, 1.5 mmol) afforded di-*tert*-butyl butane-1,4-diylbis((3-(4-oxo-4-((3-phenylpropyl)amino)butanamido)propyl)carbamate) as a yellow red gum/oil (42 mg, 20%). Following general procedure D, a sub-sample of this material (30 mg, 0.036 mmol) was reacted with TFA (0.2 mL) in CH_2_Cl_2_ (2 mL). Purification was achieved by C_8_ reversed-phase column chromatography (MeOH (+0.05% TFA):H_2_O (+0.05% TFA), 0:100→3:1) to afford the di-TFA salt **14a** as a colorless oil (27 mg, 87%). R*_f_* = 0.80 (RP-18, MeOH: 10% aq. HCl, 9:1); IR (ATR) *v*_max_ 3295, 3087, 2934, 2857, 1637, 1551, 1199, 1178, 1128 cm^−1^; ^1^H NMR (DMSO-*d_6_*, 400 MHz) δ 8.57–8.46 (4H, br s, NH_2_-17), 8.03 (2H, t, *J* = 5.8 Hz, H-13), 7.87 (2H, t *J* = 5.4 Hz, H-5), 7.29–7.26 (4H, m, H-11), 7.20–7.15 (6H, m, H-10, H-12), 3.11 (4H, dt, *J* = 6.4, 6.4 Hz, H_2_-14), 3.03 (4H, dt, J = 6.6, 6.6 Hz, H_2_-6), 2.94–2.84 (8H, br s, H_2_-16, H_2_-18), 2.56 (4H, t, *J* = 7.5 Hz, H_2_-8), 2.36–2.26 (8H, br s, H_2_-2, H_2_-3), 1.74–1.63 (8H, m, H_2_-7, H_2_-15), 1.63–1.57 (4H, m, H_2_-19); ^13^C NMR (DMSO-*d_6_*, 100 MHz) δ 172.2 (C-1), 171.2 (C-4), 141.7 (C-9), 128.3 (C-10, C-11), 125.7 (C-12), 46.1, 44.5 (C-16, C-18), 38.1 (C-6), 35.5 (C-14), 32.5 (C-8), 30.9, 30.7, 30.6 (C-2, C-3, C-7), 26.1 (C-15), 22.7 (C-19); (+)-HRESIMS [M+Na]^+^ *m*/*z* 659.4231 (calcd for C_36_H_56_N_6_NaO_4_, 659.4255).

##### *N*^1^,*N*^6^-Bis(3-(4-oxo-4-((3-phenylpropyl)amino)butanamido)propyl)hexane-1,6-diaminium 2,2,2-trifluoroacetate (**14b**)

Following general procedure C, reaction of 4-oxo-4-((3-phenylpropyl)amino)butanoic acid (**8**) (68 mg, 0.29 mmol) with di-*tert*-butyl hexane-1,6-diylbis((3-aminopropyl)carbamate) (**5b**) (50 mg, 0.12 mmol), EDC·HCl (62 mg, 0.32 mmol) and DMAP (71 mg, 0.58 mmol) afforded di-*tert*-butyl hexane-1,6-diylbis((3-(4-oxo-4-((3-phenylpropyl)amino)butanamido)propyl)carbamate) as a colorless oil (65 mg, 65%). Following general procedure D, a sub-sample of this material (37 mg, 0.043 mmol) was reacted with TFA (0.2 mL) in CH_2_Cl_2_ (2 mL). Purification was achieved by C_8_ reversed-phase column chromatography (MeOH (+0.05% TFA):H_2_O (+0.05% TFA), 0:100→3:1) to afford the di-TFA salt **14b** as a colorless oil (35 mg, 92%). R*_f_* = 0.37 (RP-18, MeOH:10% aq. HCl, 7:3); IR (ATR) ν_max_ 3289, 3085, 2862, 1665, 1549, 1444, 1259, 1178, 800, 755 cm^−1^; ^1^H NMR (CD_3_OD, 400 MHz) δ 7.28–7.23 (4H, m, H-11), 7.20–7.13 (6H, m, H-10, H-12), 3.29 (4H, br t, *J* = 6.4 Hz, H_2_-14), 3.17 (4H, t, *J* = 7.0 Hz, H_2_-6), 3.00 (4H, t, *J* = 7.0 Hz, H_2_-16), 2.93 (4H, br t, *J* = 7.3 Hz, H_2_-18), 2.63 (4H, br t, *J* = 7.3 Hz, H_2_-8), 2.54–2.45 (8H, m, H_2_-2, H_2_-3), 1.89–1.82 (4H, m, H_2_-15), 1.83–1.75 (4H, m, H_2_-7), 1.71–1.63 (4H, m, H_2_-19), 1.43–1.37 (4H, m, H_2_-20); ^13^C NMR (CD_3_OD, 100 MHz) δ 176.1 (C-1), 174.4 (C-4), 143.0 (C-9), 129.4 (C-10, C-11), 126.9 (C-12), 48.9 (C-18), 46.1 (C-16), 40.1 (C-6), 36.7 (C-14), 34.2 (C-8), 32.3 (C-7), 31.9, 31.7 (C-2, C-3), 27.7, 27.0, 26.9 (C-15, C-19, C-20); (+)-HRESIMS [M+H]^+^ *m*/*z* 665.4731 (calcd for C_38_H_61_N_6_O_4_, 665.4749).

##### *N*^1^,*N*^7^-Bis(3-(4-oxo-4-((3-phenylpropyl)amino)butanamido)propyl)heptane-1,7-diaminium 2,2,2-trifluoroacetate (**14c**)

Following general procedure C, reaction of 4-oxo-4-((3-phenylpropyl)amino)butanoic acid (**8**) (66 mg, 0.28 mmol) with di-tert-butyl heptane-1,7-diylbis((3-aminopropyl)carbamate) (**5c**) (50 mg, 0.11 mmol), EDC·HCl (60 mg, 0.31 mmol) and DMAP (69 mg, 0.56 mmol) afforded di-*tert*-butyl heptane-1,7-diylbis((3-(4-oxo-4-((3-phenylpropyl)amino)butanamido)propyl)carbamate) as a colorless oil (83 mg, 84%). Following general procedure D, a sub-sample of this material (28 mg, 0.032 mmol) was reacted with TFA (0.2 mL) in CH_2_Cl_2_ (2 mL). Purification was achieved by C_8_ reversed-phase column chromatography (MeOH (+0.05% TFA):H_2_O (+0.05% TFA), 0:100→3:1) to afford the di-TFA salt **14c** as a colorless oil (26 mg, 90%). R*_f_* = 0.50 (MeOH:10% aq. HCl, 7:3); IR (ATR) *v*_max_ 3290, 3091, 2942, 1646, 1559, 1455, 1201, 1132, 799, 721 cm^−1^; ^1^H NMR (CD_3_OD, 400 MHz) δ 7.28–7.23 (4H, m, H-11), 7.20–7.13 (6H, m, H-10, H-12), 3.29 (4H, br t, *J* = 6.4 Hz, H_2_-14), 3.17 (4H, t, *J* = 7.0 Hz, H_2_-6), 3.00 (4H, t, *J* = 7.0 Hz, H_2_-16), 2.92 (4H, br t, *J* = 7.5 Hz, H_2_-18), 2.63 (4H, br t, *J* = 7.3 Hz, H_2_-8), 2.54–2.45 (8H, m, H_2_-2, H_2_-3), 1.89–1.82 (4H, m, H_2_-15), 1.83–1.75 (4H, m, H_2_-7), 1.70–1.61 (4H, m, H_2_-19), 1.41-1.35 (6H, br s, H_2_-20, H_2_-21); ^13^C NMR (CD_3_OD, 100 MHz) δ 176.2 (C-1), 174.5 (C-4), 143.0 (C-9), 129.4 (C-10, C-11), 126.9 (C-12), 48.9 (C-18), 46.1 (C-16), 40.1 (C-6), 36.7 (C-14), 34.2 (C-8), 32.3 (C-7), 31.9, 31.7 (C-2, C-3), 29.7 (C-21), 27.7 (C-15), 27.3, 27.2 (C-19, C-20); (+)-HRESIMS [M+H]^+^ *m*/*z* 679.4924 (calcd for C_39_H_63_N_6_O_4_, 679.4905).

##### *N*^1^,*N*^8^-Bis(3-(4-oxo-4-((3-phenylpropyl)amino)butanamido)propyl)octane-1,8-diaminium 2,2,2-trifluoroacetate **(14d**)

Following general procedure C, reaction of 4-oxo-4-((3-phenylpropyl)amino)butanoic acid (**8**) (65 mg, 0.28 mmol) with di-*tert*-butyl octane-1,8-diylbis((3-aminopropyl)carbamate) (**5d**) (50 mg, 0.11 mmol), EDC·HCl (59 mg, 0.31 mmol) and DMAP (67 mg, 0.55 mmol) afforded di-*tert*-butyl octane-1,8-diylbis((3-(4-oxo-4-((3-phenylpropyl)amino)butanamido)propyl)carbamate) as a colorless oil (53 mg, 54%). Following general procedure D, the product (53 mg, 0.059 mmol) was reacted with TFA (0.2 mL) in CH_2_Cl_2_ (2 mL). Purification was achieved by C_8_ reversed-phase column chromatography (MeOH (+0.05% TFA):H_2_O (+0.05% TFA), 0:100→3:1) to afford the di-TFA salt **14d** as a colorless oil (48 mg, 88%). R*_f_* = 0.38 (RP-18, MeOH:10% aq. HCl, 7:3); IR (ATR) ν_max_ 3288, 3085, 2936, 1643, 1553, 1454, 1200, 1130, 799, 720 cm^−1^; ^1^H NMR (CD_3_OD, 400 MHz) δ 7.28–7.23 (4H, m, H-11), 7.20–7.13 (6H, m, H-10, H-12), 3.29 (4H, br t, *J* = 6.4 Hz, H_2_-14), 3.17 (4H, t, *J* = 7.0 Hz, H_2_-6), 3.00 (4H, t, *J* = 7.0 Hz, H_2_-16), 2.92 (4H, br t, *J* = 7.3 Hz, H_2_-18), 2.63 (4H, br t, *J* = 7.3 Hz, H_2_-8), 2.54–2.45 (8H, m, H_2_-2, H_2_-3), 1.89–1.82 (4H, m, H_2_-15), 1.83–1.75 (4H, m, H_2_-7), 1.70–1.61 (4H, m, H_2_-19), 1.41–1.32 (8H, br s, H_2_-20, H_2_-21); ^13^C NMR (CD_3_OD, 100 MHz) δ 176.1 (C-1), 174.4 (C-4), 143.0 (C-9), 129.4 (C-10, C-11), 126.9 (C-12), 49.1 (C-18), 46.1 (C-16), 40.0 (C-6), 36.7 (C-14), 34.2 (C-8), 32.3 (C-7), 31.9, 31.7 (C-2, C-3), 29.9 (C-21), 27.7 (C-15), 27.4, 27.2 (C-19, C-20); (+)-HRESIMS [M+2H]^2+^ *m*/*z* 347.2564 (calcd for C_40_H_66_N_6_O_4_, 347.2567).

##### *N*^1^,*N*^10^-Bis(3-(4-oxo-4-((3-phenylpropyl)amino)butanamido)propyl)decane-1,10-diaminium 2,2,2-trifluoroacetate (**14e**)

Following general procedure C, reaction of 4-oxo-4-((3-phenylpropyl)amino)butanoic acid (**8**) (53.2 mg, 0.23 mmol) with di-*tert*-butyl decane 1,10-diylbis((3-aminopropyl)carbamate) (**5e**) (50 mg, 0.10 mmol), EDC·HCl (49.8 mg, 0.26 mmol), HOBt (35.1 mg, 0.26 mmol) and DIPEA (0.105 mL, 0.60 mmol) afforded di-*tert*-butyl decane-1,10-diylbis((3-(4-oxo-4-((3-phenylpropyl)amino)butanamido)propyl)carbamate) as a yellow gum (66 mg, 72%). Following general procedure D, this product (66 mg, 0.072 mmol) was reacted with TFA (0.2 mL) in CH_2_Cl_2_ (2 mL). Purification was achieved by C_8_ reversed-phase column chromatography (MeOH (+0.05% TFA):H_2_O (+0.05% TFA), 0:100→3:1) to afford the di-TFA salt **14e** as an orange oil (57 mg, 84%). R*_f_* = 0.83 (RP-18, MeOH:10% aq. HCl, 9:1); IR (ATR) *v*_max_ 3283, 3028, 2929, 2858, 1645, 1549, 1199, 1172, 1127 cm^−1^; ^1^H NMR (DMSO-*d_6_*, 400 MHz) δ 8.34–8.23 (4H, m, NH_2_-17), 8.03 (2H, t, *J* = 6.0 Hz, NH-13), 7.86 (2H, t, *J* = 5.5 Hz, NH-5), 7.30–7.24 (4H, m, H_2_-11), 7.21–7.14 (6H, m, H_2_-10, H_2_-12), 3.12 (4H, dt, *J* = 6.4, 6.4 Hz, H_2_-14), 3.03 (4H, dt, *J* = 6.6, 6.6 Hz, H_2_-6), 2.92–2.85 (4H, br s, H_2_-16), 2.85–2.78 (4H, br s, H_2_-18), 2.56 (4H, t, *J* = 7.8 Hz, H_2_-8), 2.36–2.26 (8H, m, H_2_-2, H_2_-3), 1.74–1.62 (8H, m, H_2_-7, H_2_-15), 1.58–1.48 (4H, m, H_2_-19), 1.30–1.20 (12H, m, H_2_-20, H_2_-21, H_2_-22); ^13^C NMR (DMSO-*d_6_*, 100 MHz) δ 172.3 (C-1), 171.2 (C-4), 141.7 (C-9), 128.3 (C-10, C-11), 125.7 (C-12), 46.8 (C-18), 44.5 (C-16), 38.1 (C-6), 35.4 (C-14), 32.5 (C-8), 30.9, 30.6, 30.5 (C-2, C-3, C-7), 28.8, 28.5, 26.2, 25.9, 25.5 (C-15, C-19, C-20, C-21, C-22); (+)-HRESIMS [M+H]^+^ *m*/*z* 723.5506 (calcd for C_42_H_71_N_6_O_4_, 723.5531).

##### *N*^1^,*N*^12^-Bis(3-(4-oxo-4-((3-phenylpropyl)amino)butanamido)propyl)dodecane-1,12-diaminium 2,2,2-trifluoroacetate (**14f**)

Following general procedure C, reaction of 4-oxo-4-((3-phenylpropyl)amino)butanoic acid (**8**) (56 mg, 0.24 mmol) with di-*tert*-butyl dodecane-1,12-diylbis((3-aminopropyl)carbamate) (**5f**) (50 mg, 0.097 mmol), EDC·HCl (52 mg, 0.27 mmol) and DMAP (60 mg, 0.49 mmol) afforded di-*tert*-butyl dodecane-1,12-diylbis((3-(4-oxo-4-((3-phenylpropyl)amino)butanamido)propyl)carbamate) as a colorless oil (40 mg, 43%). Following general procedure D, a sub-sample of this material (27 mg, 0.028 mmol) was reacted with TFA (0.2 mL) in CH_2_Cl_2_ (2 mL). Purification was achieved by C_8_ reversed-phase column chromatography (MeOH (+0.05% TFA):H_2_O (+0.05% TFA), 0:100→3:1) to afford the di-TFA salt **14f** as a colorless oil (27 mg, 97%). R*_f_* = 0.15 (RP-18, MeOH:10% HCl 7:3); IR (ATR) ν_max_ 3292, 1671, 1556, 1437, 1202, 1133, 800, 722 cm^−1^; ^1^H NMR (CD_3_OD, 400 MHz) δ 7.27–7.23 (4H, m, H-11), 7.20–7.12 (6H, m, H-10, H-12), 3.30 (4H, br t, *J* = 6.5 Hz, H_2_-14), 3.17 (4H, t, *J* = 7.3 Hz, H_2_-6), 3.01 (4H, t, *J* = 7.0 Hz, H_2_-16), 2.92 (4H, br t, *J* = 8.0 Hz, H_2_-18), 2.63 (4H, br t, *J* = 8.0 Hz, H_2_-8), 2.55–2.44 (8H, m, H_2_-2, H_2_-3), 1.89–1.82 (8H, m, H_2_-15), 1.82–1.76 (4H, m, H_2_-7), 1.70–1.60 (4H, m, H_2_-19), 1.38–1.28 (16H, m, H_2_-20, H_2_-21, H_2_-22, H_2_-23); ^13^C NMR (CD_3_OD, 100 MHz) δ 176.2 (C-1), 174.5 (C-4), 143.0 (C-9), 129.4 (C-10, C-11), 126.9 (C-12), 49.1 (C-18), 46.1 (C-16), 40.1 (C-6), 36.6 (C-14), 34.2 (C-8), 32.3 (C-7), 31.8, 31.6 (C-2, C-3), 30.6, 30.5, 30.2 (C-21, C-22, C-23), 27.7, 27.5, 27.3 (C-15, C-19, C-20); (+)-HRESIMS [M+2H]^2+^ *m*/*z* 375.2887 (calcd for C_44_H_74_N_6_O_4_, 375.2880).

##### *N*^1^,*N*^4^-Bis(3-(3,3-diphenylpropanamido)propyl)butane-1,4-diaminium 2,2,2-trifluoroacetate (**15a**)

Following general procedure C, reaction of 3,3-diphenylpropionic acid (**9**) (62 mg, 0.27 mmol) with di-*tert*-butyl butane 1,4-diylbis((3-aminopropyl)carbamate) (**5a**) (50 mg, 0.12 mmol), EDC·HCl (62 mg, 0.32 mmol), HOBt (44 mg, 0.32 mmol) and DIPEA (0.13 mL, 0.74 mmol) afforded di-*tert*-butyl butane-1,4-diylbis((3-(3,3-diphenylpropanamido)propyl)carbamate) as a colorless oil (38 mg, 37%). Following general procedure D, a sub-sample of this material (15 mg, 0.018 mmol) was reacted with TFA (0.2 mL) in CH_2_Cl_2_ (2 mL). Purification was achieved by C_8_ reversed-phase column chromatography (MeOH (+0.05% TFA):H_2_O (+0.05% TFA), 0:100→3:1) to afford the di-TFA salt **15a** as a colorless oil (13 mg, 86%). R*_f_* = 0.51 (RP-18, MeOH:10% aq. HCl, 5:1); IR (ATR) *v*_max_ 3420, 3280, 3065, 3028, 2938, 2849, 2497, 1672, 1641, 1199, 1175, 1126, 719, 699 cm^−1^; ^1^H NMR (DMSO-*d_6_*, 400 MHz) δ 8.53–8.43 (4H, br s, NH_2_-12), 8.18 (2H, t, *J* = 5.9 Hz, NH-8), 7.32–7.25 (16H, m, H-5, H-6), 7.20–7.14 (4H, m, H-7), 4.48 (2H, t, *J* = 8.1 Hz, H-3), 3.05 (4H, dt, *J* = 6.4, 6.2 Hz, H_2_-9), 2.88 (4H, d, *J* = 8.1 Hz, H_2_-2), 2.75–2.67 (4H, br s, H_2_-13), 2.58–2.52 (4H, m, H_2_-11), 1.61–1.56 (4H, m, H_2_-10), 1.56–1.51 (4H, m, H_2_-14); ^13^C NMR (DMSO-*d_6_*, 100 MHz) δ 171.0 (C-1), 144.1 (C-4), 128.4 (C-6), 127.5 (C-5), 126.2 (C-7), 46.8 (C-3), 46.1 (C-13), 44.2 (C-11), 41.1 (C-2), 35.2 (C-9), 26.0 (C-10), 22.6 (C-14); (+)-HRESIMS [M+H]^+^ *m*/*z* 619.4000 (calcd for C_40_H_51_N_4_O_2_, 619.4007).

##### *N*^1^,*N*^6^-Bis(3-(3,3-diphenylpropanamido)propyl)hexane-1,6-diaminium 2,2,2-trifluoroacetate (**15b**)

Following general procedure C, reaction of 3,3-diphenylpropionic acid (**9**) (58 mg, 0.26 mmol) with di-*tert*-butyl hexane-1,6-diylbis((3-aminopropyl)carbamate) (**5b**) (50 mg, 0.12 mmol), EDC·HCl (58 mg, 0.30 mmol), HOBt (41 mg, 0.30 mmol) and DIPEA (0.12 mL, 0.70 mmol) afforded di-*tert*-butyl hexane-1,6-diylbis((3-(3,3-diphenylpropanamido)propyl)carbamate) as a colorless oil (42 mg, 43%). Following general procedure D, a sub-sample of this material (20 mg, 0.024 mmol) was reacted with TFA (0.2 mL) in CH_2_Cl_2_ (2 mL). Purification was achieved by C_8_ reversed-phase column chromatography (MeOH (+0.05% TFA):H_2_O (+0.05% TFA), 0:100→3:1) to afford the di-TFA salt **15b** as a colorless oil (18 mg, 86%). R*_f_* = 0.51 (RP-18, MeOH:10% aq. HCl, 5:1); IR (ATR) *v*_max_ 3405, 3282, 3028, 2940, 2851, 2500, 1673, 1642, 1555, 1199, 1174, 1127, 719, 701 cm^−1^; ^1^H NMR (DMSO-*d_6_*, 400 MHz) δ 8.44–8.35 (4H, m, NH_2_-12), 8.18 (2H, t, *J* = 5.9 Hz, NH-8), 7.30–7.24 (16H, m, H-5, H-6), 7.18–7.14 (4H, m, H-7), 4.48 (2H, t, *J* = 8.1 Hz, H-3), 3.05 (4H, dt, *J* = 6.3, 6.1 Hz, H_2_-9), 2.88 (4H, d, *J* = 8.1 Hz, H_2_-2), 2.71–2.65 (4H, m, H_2_-13), 2.56–2.50 (4H, m, H_2_-11), 1.60–1.54 (4H, m, H_2_-10), 1.54–1.46 (4H, m, H_2_-14), 1.33–1.23 (4H, m, H_2_-15); ^13^C NMR (DMSO-*d_6_*, 100 MHz) δ 171.0 (C-1), 144.1 (C-4), 128.4, 127.5 (C-5, C-6), 126.2 (C-7), 46.8 (C-3), 46.7 (C-13), 44.2 (C-11), 41.1 (C-2), 35.2 (C-9), 26.1 (C-10), 25.5, 25.3 (C-14, C-15); (+)-HRESIMS [M+H]^+^ *m*/*z* 647.4295 (calcd for C_42_H_55_N_4_O_2_, 647.4320).

##### *N*^1^,*N*^7^-Bis(3-(3,3-diphenylpropanamido)propyl)heptane-1,7-diaminium 2,2,2-trifluoroacetate (**15c**)

Following general procedure C, reaction of 3,3-diphenylpropionic acid (**9**) (56 mg, 0.25 mmol) with di-*tert*-butyl heptane-1,7-diylbis((3-aminopropyl)carbamate) (**5c**) (50 mg, 0.11 mmol), EDC·HCl (56 mg, 0.29 mmol), HOBt (39 mg, 0.29 mmol) and DIPEA (0.12 mL, 0.67 mmol) afforded di-*tert*-butyl heptane-1,7-diylbis((3-(3,3-diphenylpropanamido)propyl)carbamate) as a colorless oil (54 mg, 56%). Following general procedure D, a sub-sample of this material (20 mg, 0.023 mmol) was reacted with TFA (0.2 mL) in CH_2_Cl_2_ (2 mL). Purification was achieved by C_8_ reversed-phase column chromatography (MeOH (+0.05% TFA):H_2_O (+0.05% TFA), 0:100→3:1) to afford the di-TFA salt **15c** as a colorless oil (19 mg, 93%). R*_f_* = 0.49 (RP-18, MeOH:10% aq. HCl, 5:1); IR (ATR) *v*_max_ 3405, 3282, 3075, 3028, 2938, 2858, 2495, 1673, 1644, 1199, 1174, 1127, 719, 700 cm^−1^; ^1^H NMR (DMSO-*d_6_*, 400 MHz) δ 8.44–8.34 (4H, m, NH_2_-12), 8.17 (2H, t, *J* = 5.8 Hz, NH-8), 7.32–7.24 (16H, m, H-5, H-6), 7.19–7.14 (4H, m, H-7), 4.48 (2H, t, *J* = 8.2 Hz, H-3), 3.05 (4H, dt, *J* = 6.3, 6.1 Hz, H_2_-9), 2.88 (4H, d, *J* = 8.2 Hz, H_2_-2), 2.70–2.64 (4H, m, H_2_-13), 2.56–2.50 (4H, m, H_2_-11), 1.60–1.53 (4H, m, H_2_-10), 1.53–1.47 (4H, m, H_2_-14), 1.31–1.25 (6H, m, H_2_-15, H_2_-16); ^13^C NMR (DMSO-*d_6_*, 100 MHz) δ 171.0 (C-1), 144.1 (C-4), 128.4 (C-6), 127.5 (C-5), 126.2 (C-7), 46.83 (C-3), 46.75 (C-13), 44.2 (C-11), 41.1 (C-2), 35.2 (C-9), 28.0 (C-15 or C-16), 26.1 (C-10), 25.7, 25.4 (C-14, C-15 or C-16); (+)-HRESIMS [M+H]^+^ *m*/*z* 661.4460 (calcd for C_43_H_57_N_4_O_2_, 661.4476).

##### *N*^1^,*N*^8^-Bis(3-(3,3-diphenylpropanamido)propyl)octane-1,8-diaminium 2,2,2-trifluoroacetate (**15d**)

Following general procedure C, reaction of 3,3-diphenylpropionic acid (**9**) (54 mg, 0.24 mmol) with di-*tert*-butyl octane-1,8-diylbis((3-aminopropyl)carbamate) (**5d**) (50 mg, 0.11 mmol), EDC·HCl (54 mg, 0.28 mmol), HOBt (38 mg, 0.28 mmol) and DIPEA (0.11 mL, 0.65 mmol) afforded di-*tert*-butyl octane-1,8-diylbis((3-(3,3-diphenylpropanamido)propyl)carbamate) as a colorless oil (51 mg, 53%). Following general procedure D, a sub-sample of this material (20 mg, 0.023 mmol) was reacted with TFA (0.2 mL) in CH_2_Cl_2_ (2 mL). Purification was achieved by C_8_ reversed-phase column chromatography (MeOH (+0.05% TFA):H_2_O (+0.05% TFA), 0:100→3:1) to afford the di-TFA salt **15d** as a colorless oil (17 mg, 82%). R*_f_* = 0.46 (RP-18, MeOH:10% aq. HCl, 5:1); IR (ATR) *v*_max_ 3418, 3281, 3068, 3028, 2936, 2858, 2485, 1672, 1643, 1199, 1174, 1127, 719, 700 cm^−1^; ^1^H NMR (DMSO-*d_6_*, 400 MHz) δ 8.41–8.31 (4H, m, NH_2_-12), 8.17 (2H, t, *J* = 8.1 Hz, NH-8), 7.32–7.24 (16H, m, H-5, H-6), 7.18–7.14 (4H, m, H-7), 4.48 (2H, t, *J* = 8.1 Hz, H-3), 3.05 (4H, dt, *J* = 6.3, 6.1 Hz, H_2_-9), 2.87 (4H, d, *J* = 8.1 Hz, H_2_-2), 2.70–2.63 (4H, m, H_2_-13), 2.55–2.50 (4H, m, H_2_-11), 1.60–1.52 (4H, m, H_2_-10), 1.52–1.47 (4H, m, H_2_-14), 1.32–1.25 (8H, m, H_2_-15, H_2_-16); ^13^C NMR (DMSO-*d_6_*, 100 MHz) δ 171.0 (C-1), 144.1 (C-4), 128.4 (C-6), 127.5 (C-5), 126.2 (C-7), 46.81, 46.78 (C-3, C-13), 44.2 (C-11), 41.1 (C-2), 35.2 (C-9), 28.3 (C-15 or C-16), 26.1 (C-10), 25.8, 25.4 (C-14, C-15 or C-16); (+)-HRESIMS [M+H]^+^ *m*/*z* 675.4611 (calcd for C_44_H_59_N_4_O_2_, 675.4633).

##### *N*^1^,*N*^10^-Bis(3-(3,3-diphenylpropanamido)propyl)decane-1,10-diaminium 2,2,2-trifluoroacetate (**15e**)

Following general procedure C, reaction of 3,3-diphenylpropionic acid (**9**) (51 mg, 0.23 mmol) with di-*tert*-butyl decane-1,10-diylbis((3-aminopropyl)carbamate) (**5e**) (50 mg, 0.10 mmol), EDC·HCl (51 mg, 0.27 mmol), HOBt (36 mg, 0.27 mmol) and DIPEA (0.11 mL, 0.62 mmol) afforded di-*tert*-butyl decane-1,10-diylbis((3-(3,3-diphenylpropanamido)propyl)carbamate) as a colorless oil (50 mg, 54%). Following general procedure D, a sub-sample of this material (20 mg, 0.022 mmol) was reacted with TFA (0.2 mL) in CH_2_Cl_2_ (2 mL). Purification was achieved by C_8_ reversed-phase column chromatography (MeOH (+0.05% TFA):H_2_O (+0.05% TFA), 0:100→3:1) to afford the di-TFA salt **15e** as a colorless oil (16 mg, 78%). R*_f_* = 0.37 (RP-18, MeOH:10% aq. HCl, 5:1); IR (ATR) *v*_max_ 3420, 3281, 3080, 3028, 2932, 2857, 2502, 1673, 1644, 1200, 1175, 1128, 719, 700 cm^−1^; ^1^H NMR (DMSO-*d_6_*, 400 MHz) δ 8.44–8.35 (4H, m, NH_2_-12), 8.18 (2H, t, *J* = 5.8 Hz, NH-8), 7.29–7.24 (16H, m, H-5, H-6), 7.19–7.13 (4H, m, H-7), 4.48 (2H, t, *J* = 8.1 Hz, H-3), 3.04 (4H, dt, *J* = 6.3, 6.0 Hz, H_2_-9), 2.89 (4H, d, *J* = 8.1 Hz, H_2_-2), 2.69–2.63 (4H, m, H_2_-13), 2.55–2.48 (4H, m, H_2_-11), 1.59–1.53 (4H, m, H_2_-10), 1.53–1.46 (4H, m, H_2_-14), 1.32–1.23 (12H, m, H_2_-15, H_2_-16, H_2_-17); ^13^C NMR (DMSO-*d_6_*, 100 MHz) δ 171.0 (C-1), 144.1 (C-4), 128.4 (C-6), 127.5 (C-5), 126.2 (C-7), 46.83, 46.80 (C-3, C-13), 44.2 (C-11), 41.1 (C-2), 35.2 (C-9), 28.7, 28.5 (C-15 or C-16 or C-17), 26.0, 25.9, 25.4 (C-10, C-14, C-15 or C-16 or C-17); (+)-HRESIMS [M+H]^+^ *m*/*z* 703.4926 (calcd for C_46_H_63_N_4_O_2_, 703.4946).

##### *N*^1^,*N*^12^-Bis(3-(3,3-diphenylpropanamido)propyl)dodecane-1,12-diaminium 2,2,2-trifluoroacetate (**15f**)

Following general procedure C, reaction of 3,3-diphenylpropionic acid (**9**) (48 mg, 0.21 mmol) with di-*tert*-butyl dodecane-1,12-diylbis((3-aminopropyl)carbamate) (**5f**) (50 mg, 0.10 mmol), EDC·HCl (48 mg, 0.25 mmol), HOBt (34 mg, 0.25 mmol) and DIPEA (0.10 mL, 0.58 mmol) afforded di-*tert*-butyl dodecane-1,12-diylbis((3-(3,3-diphenylpropanamido)propyl)carbamate) as a colorless oil (42 mg, 47%). Following general procedure D, a sub-sample of this material (20 mg, 0.021 mmol) was reacted with TFA (0.2 mL) in CH_2_Cl_2_ (2 mL). Purification was achieved by C_8_ reversed-phase column chromatography (MeOH (+0.05% TFA):H_2_O (+0.05% TFA), 0:100→3:1) to afford the di-TFA salt **15f** as a colorless oil (19 mg, 95%). R*_f_* = 0.60 (RP-18, MeOH:10% aq. HCl, 9:1); IR (ATR) *v*_max_ 3420, 3282, 3075, 3028, 2928, 2855, 2477, 1672, 1643, 1199, 1174, 1128, 719, 700 cm^−1^; ^1^H NMR (DMSO-*d_6_*, 400 MHz) δ 8.34–8.24 (4H, m, NH_2_-12), 8.16 (2H, t, *J* = 5.9 Hz, NH-8), 7.30–7.24 (16H, m, H-5, H-6), 7.19–7.14 (4H, m, H-7), 4.47 (2H, t, *J* = 8.1 Hz, H-3), 3.05 (4H, dt, *J* = 6.4, 6.2 Hz, H_2_-9), 2.87 (4H, d, *J* = 8.1 Hz, H_2_-2), 2.69–2.62 (4H, m, H_2_-13), 2.54–2.48 (4H, m, H_2_-11), 1.59–1.52 (4H, m, H_2_-10), 1.52–1.45 (4H, m, H_2_-14), 1.32–1.23 (16H, m, H_2_-15, H_2_-16, H_2_-17, H_2_-18); ^13^C NMR (DMSO-*d_6_*, 100 MHz) δ 171.1 (C-1), 144.1 (C-4), 128.4 (C-6), 127.5 (C-5), 126.2 (C-7), 46.8 (C-3, C-13), 44.2 (C-11), 41.1 (C-2), 35.2 (C-9), 29.0, 28.9, 28.5 (C-15 or C-16 or C-17 or C-18), 26.1 (C-10), 25.9, 25.4 (C-14, C-15 or C-16 or C-17 or C-18); (+)-HRESIMS [M+H]^+^ *m*/*z* 731.5232 (calcd for C_48_H_67_N_4_O_2_, 731.5259).

##### *N*^1^,*N*^4^-Bis(3-(4-((3,3-diphenylpropyl)amino)-4-oxobutanamido)propyl)butane-1,4-diaminium 2,2,2-trifluoroacetate (**16a**)

Following general procedure C, reaction of 4-((3,3-diphenylpropyl)amino)-4-oxobutanoic acid (**10**) (171 mg, 0.55 mmol) with di-*tert*-butyl butane-1,4-diylbis((3-aminopropyl)carbamate) (**5a**) (100 mg, 0.25 mmol), HBTU (237 mg, 0.63 mmol) and DIPEA (0.26 mL, 1.5 mmol) afforded di-*tert*-butyl butane-1,4-diylbis((3-(4-((3,3-diphenylpropyl)amino)-4-oxobutanamido) propyl)carbamate) as a colorless oil (45 mg, 18%). Following general procedure D, a sub-sample of this material (40 mg, 0.040 mmol) was reacted with TFA (0.2 mL) in CH_2_Cl_2_ (2 mL). Purification was achieved by C_8_ reversed-phase column chromatography (MeOH (+0.05% TFA):H_2_O (+0.05% TFA), 0:100→3:1) to afford the di-TFA salt **16a** as a colorless oil (27 mg, 68%). R*_f_* = 0.69 (RP-18, MeOH:10% aq. HCl, 9:1); IR (ATR) *v*_max_ 3288, 3029, 2940, 2857, 1669, 1641, 1199, 1176, 1126 cm-1; ^1^H NMR (DMSO-*d*_6_, 400 MHz) δ 8.57 (4H, br s, NH_2_-17), 8.03 (2H, t, *J* = 5.8 Hz, NH-13), 7.89 (2H, t, *J* = 5.4 Hz, NH-5), 7.30–7.25 (16H, m, H-10, H-11), 7.18–7.14 (4H, m, H-12), 3.97 (2H, t, *J* = 8.0 Hz, H-8), 3.11 (4H, dt, *J* = 6.4, 6.4 Hz, H_2_-14), 2.95–2.89 (4H, m, H_2_-6), 2.89–2.83 (8H, m, H_2_-16, H_2_-18), 2.39–2.27 (8H, m, H_2_-2, H_2_-3), 2.14 (4H, dt, *J* = 8.8, 6.8 Hz, H_2_-7), 1.71 (4H, tt, *J* = 7.2, 7.2 Hz, H_2_-15), 1.64–1.56 (4H, m, H_2_-19); ^13^C NMR (DMSO-*d*_6_, 100 MHz) δ 172.1 (C-1), 171.2 (C-4), 144.8 (C-9), 128.4 (C-11), 127.6 (C-10), 126.1 (C-12), 47.9 (C-8), 46.1 (C-18), 44.5 (C-16), 37.4 (C-6), 35.5 (C-14), 34.6 (C-7), 30.7, 30.6 (C-2, C-3), 26.1 (C-14), 22.7 (C-19); (+)-HRESIMS [M+Na]^+^ *m*/*z* 811.4862 (calcd for C_48_H_64_N_6_NaO_4_, 811.4881).

##### *N*^1^,*N*^6^-Bis(3-(4-((3,3-diphenylpropyl)amino)-4-oxobutanamido)propyl)hexane-1,6-diaminium 2,2,2-trifluroracetate (**16b**)

Following general procedure C, reaction of 4-((3,3-diphenylpropyl)amino)-4-oxobutanoic acid (**10**) (48 mg, 0.15 mmol) with di-*tert*-butyl hexane-1,6-diylbis((3-aminopropyl)carbamate) (**5b**) (30 mg, 0.07 mmol), EDC·HCl (35 mg, 0.18 mmol), HOBt (25 mg, 0.18 mmol) and DIPEA (0.07 mL, 0.42 mmol) afforded di-*tert*-butyl hexane-1,6-diylbis((3-(4-((3,3-diphenylpropyl)amino)-4-oxobutanamido)propyl)carbamate) as a colorless oil (40 mg, 56%). Following general procedure D, a sub-sample of the material (20 mg, 0.020 mmol) was reacted with TFA (0.2 mL) in CH_2_Cl_2_ (2 mL). Purification was achieved by C_8_ reversed-phase column chromatography (MeOH (+0.05% TFA):H_2_O (+0.05% TFA), 0:100→3:1) to afford the di-TFA salt **16b** as a colorless oil (14 mg, 68%). R*_f_* = 0.39 (RP-18, MeOH:10% aq. HCl, 5:1); IR (ATR) *v*_max_ 3392, 3287, 3086, 2917, 2849, 1645, 1554, 1201, 1131, 1024, 700 cm^−1^; ^1^H NMR (DMSO-*d_6_*, 400 MHz) δ 8.39–8.30 (4H, m, NH_2_-17), 8.04 (2H, t, *J* = 5.8 Hz, NH-13), 7.89 (2H, t, *J* = 5.5 Hz, NH-5), 7.33–7.26 (16H, m, H-10, H-11), 7.22–7.15 (4H, m, H-12), 3.99 (2H, t, *J* = 7.8 Hz, H-8), 3.13 (4H, dt, *J* = 6.4, 6.2 Hz, H_2_-14), 2.98–2.90 (4H, m, H_2_-6), 2.90–2.79 (8H, m, H_2_-16, H_2_-18), 2.34–2.20 (8H, m, H_2_-2, H_2_-3), 2.16 (4H, dt, *J* = 7.8, 6.7 Hz, H_2_-7), 1.71 (4H, tt, *J* = 7.2, 6.6 Hz, H_2_-15), 1.59–1.49 (4H, m, H_2_-19), 1.33–1.23 (4H, m, H_2_-20); ^13^C NMR (DMSO-*d_6_*, 100 MHz) δ 172.2 (C-1), 171.2 (C-4), 144.7 (C-9), 128.4 (C-11), 127.6 (C-10), 126.1 (C-12), 47.9 (C-8), 46.7 (C-18), 44.5 (C-16), 37.3 (C-6), 35.4 (C-14), 34.6 (C-7), 30.6, 30.5 (C-2, C-3), 26.1 (C-15), 25.4, 25.3 (C-19, C-20); (+)-HRESIMS [M+Na]^+^ *m*/*z* 839.5166 (calcd for C_50_H_68_N_6_NaO_4_, 839.5194).

##### *N*^1^,*N*^7^-Bis(3-(4-((3,3-diphenylpropyl)amino)-4-oxobutanamido)propyl)heptane-1,7-diaminium 2,2,2-trifluoroacetate (**16c**)

Following general procedure C, reaction of 4-((3,3-diphenylpropyl)amino)-4-oxobutanoic acid (**10**) (46 mg, 0.15 mmol) with di-*tert*-butyl heptane-1,7-diylbis((3-aminopropyl)carbamate) (**5c**) (30 mg, 0.07 mmol), EDC·HCl (33 mg, 0.17 mmol), HOBt (24 mg, 0.17 mmol) and DIPEA (0.07 mL, 0.42 mmol) afforded di-*tert*-butyl heptane-1,7-diylbis((3-(4-((3,3-diphenylpropyl)amino)-4-oxobutanamido)propyl)carbamate) as a colorless oil (40 mg, 58%). Following general procedure D, a sub-sample of this material (20 mg, 0.019 mmol) was reacted with TFA (0.2 mL) in CH_2_Cl_2_ (2 mL). Purification was achieved by C_8_ reversed-phase column chromatography (MeOH (+0.05% TFA):H_2_O (+0.05% TFA), 0:100→3:1) to afford the di-TFA salt **16c** as a colorless oil (10 mg, 49%). R*_f_* = 0.39 (RP-18, MeOH:10% aq. HCl, 5:1); IR (ATR) *v*_max_ 3412, 3289, 3062, 2934, 2858, 1642, 1552, 1200, 1176, 1129, 669 cm^−1^; ^1^H NMR (DMSO-*d_6_*, 400 MHz) δ 8.42–8.31 (4H, m, NH_2_-17), 8.04 (2H, t, *J* = 5.9 Hz, NH-13), 7.89 (2H, t, *J* = 5.4 Hz, NH-5), 7.31–7.27 (16H, m, H-10, H-11), 7.21–7.15 (4H, m, H-12), 3.99 (2H, t, *J* = 7.7 Hz, H-8), 3.13 (4H, dt, *J* = 6.4, 6.3 Hz, H_2_-14), 3.00–2.91 (4H, m, H_2_-6), 2.91–2.79 (8H, m, H_2_-16, H_2_-18), 2.36–2.28 (8H, m, H_2_-2, H_2_-3), 2.16 (4H, dt, *J* = 7.7, 6.7 Hz, H_2_-7), 1.71 (4H, tt, *J* = 7.0, 6.4 Hz, H_2_-15), 1.59–1.49 (4H, m, H_2_-19), 1.31–1.22 (6H, m, H_2_-20, H_2_-21); ^13^C NMR (DMSO-*d_6_*, 100 MHz) δ 172.2 (C-1), 171.2 (C-4), 144.7 (C-9), 128.4 (C-11), 127.6 (C-10), 126.1 (C-12), 47.9 (C-8), 46.7 (C-18), 44.5 (C-16), 37.3 (C-6), 35.4 (C-14), 34.6 (C-7), 30.6, 30.5 (C-2, C-3), 28.0 (C-20 or C-21), 26.1 (C-15), 25.7, 25.4 (C-19, C-20 or C-21); (+)-HRESIMS [M+Na]^+^ *m*/*z* 853.5331 (calcd for C_51_H_70_N_6_NaO_4_, 853.5351).

##### *N*^1^,*N*^8^-Bis(3-(4-((3,3-diphenylpropyl)amino)-4-oxobutanamido)propyl)octane-1,8-diaminium 2,2,2-trifluoroacetate (**16d**)

Following general procedure C, reaction of 4-((3,3-diphenylpropyl)amino)-4-oxobutanoic acid (**10**) (45 mg, 0.14 mmol) with di-*tert*-butyl octane-1,8-diylbis((3-aminopropyl)carbamate) (**5d**) (30 mg, 0.07 mmol), EDC·HCl (32 mg, 0.17 mmol), HOBt (23 mg, 0.17 mmol) and DIPEA (0.07 mL, 0.39 mmol) afforded di-*tert*-butyl octane-1,8-diylbis((3-(4-((3,3-diphenylpropyl)amino)-4-oxobutanamido)propyl)carbamate) as a colorless oil (32 mg, 47%). Following general procedure D, a sub-sample of this material (15 mg, 0.014 mmol) was reacted with TFA (0.2 mL) in CH_2_Cl_2_ (2 mL). Purification was achieved by C_8_ reversed-phase column chromatography (MeOH (+0.05% TFA):H_2_O (+0.05% TFA), 0:100→3:1) to afford the di-TFA salt **16d** as a colorless oil (14 mg, 93%). R*_f_* = 0.37 (RP-18, MeOH:10% aq. HCl, 5:1); IR (ATR) *v*_max_ 3288, 3062, 3028, 2936, 2859, 1643, 1550, 1199, 1175, 1128, 699 cm^−1^; ^1^H NMR (DMSO-*d_6_*, 400 MHz) δ 8.46–8.38 (4H, m, NH_2_-17), 8.03 (2H, t, *J* = 5.8 Hz, NH-13), 7.88 (2H, t, *J* = 5.4 Hz, NH-5), 7.30–7.25 (16H, m, H-10, H-11), 7.19–7.14 (4H, m, H-12), 3.97 (2H, t, *J* = 7.8 Hz, H-8), 3.11 (4H, dt, *J* = 6.8, 6.2 Hz, H_2_-14), 2.96–2.89 (4H, m, H_2_-6), 2.89–2.84 (4H, m, H_2_-16), 2.84–2.77 (4H, m, H_2_-18), 2.34–2.27 (8H, m, H_2_-2, H_2_-3), 2.14 (4H, dt, *J* = 7.8, 6.9 Hz, H_2_-7), 1.70 (4H, tt, *J* = 7.1, 6.8 Hz, H_2_-15), 1.57–1.48 (4H, m, H_2_-19), 1.30–1.20 (8H, m, H_2_-20, H_2_-21); ^13^C NMR (DMSO-*d_6_*, 100 MHz) δ 172.2 (C-1), 171.2 (C-4), 144.7 (C-9), 128.4 (C-11), 127.6 (C-10), 126.1 (C-12), 47.9 (C-8), 46.8 (C-18), 44.5 (C-16), 37.3 (C-6), 35.4 (C-14), 34.6 (C-7), 30.6, 30.5 (C-2, C-3), 28.3 (C-20 or C-21), 26.1 (C-15), 25.8, 25.4 (C-19, C-20 or C-21); (+)-HRESIMS [M+H]^+^ *m*/*z* 845.5661 (calcd for C_52_H_73_N_6_O_4_, 845.5688).

##### *N*^1^,*N*^10^-Bis(3-(4-((3,3-diphenylpropyl)amino)-4-oxobutanamido)propyl)decane-1,10-diaminium 2,2,2-trifluoroacetate (**16e**)

Following general procedure C, reaction of 4-((3,3-diphenylpropyl)amino)-4-oxobutanoic acid (**10**) (72 mg, 0.23 mmol) with di-*tert*-butyl decane-1,10-diylbis((3-aminopropyl)carbamate) (**5e**) (50 mg, 0.10 mmol), HBTU (94.8 mg, 025 mmol) and DIPEA (0.105 mL, 0.6 mmol) afforded di-*tert*-butyl decane-1,10-diylbis((3-(4-((3,3-diphenylpropyl)amino)-4-oxobutanamido) propyl)carbamate) as a colorless gum (13 mg, 12%). Following general procedure D, this material (13 mg, 0.012 mmol) was reacted with TFA (0.2 mL) in CH_2_Cl_2_ (2 mL). Purification was achieved by C_8_ reversed-phase column chromatography (MeOH (+0.05% TFA):H_2_O (+0.05% TFA), 0:100→3:1) affording the di-TFA salt **16e** as a colorless oil (8 mg, 62%). R*_f_* = 0.69 (RP-18, MeOH:10% aq. HCl, 9:1); IR (ATR) *v*_max_ 3279, 3027, 2930, 2857, 1669, 1643, 1553, 1199, 1175, 1128, 720, 700 cm^−1^; ^1^H NMR (DMSO-*d*_6_, 400 MHz) δ 8.44–8.28 (4H, m, NH_2_-17), 8.02 (2H, t, *J* = 5.8 Hz, NH-13), 7.87 (2H, t, *J* = 5.4 Hz, NH-5), 7.29–7.25 (16H, m, H-10, H-11), 7.19–7.12 (4H, m, H-12), 3.97 (2H, t, *J* = 7.7 Hz, H-8), 3.11 (4H, dt, *J* = 6.2, 6.2 Hz, H_2_-14), 2.96–2.89 (4H, m, H_2_-6), 2.88–2.85 (4H, m, H_2_-16), 2.85–2.78 (4H, m, H_2_-18), 2.35–2.25 (8H, m, H_2_-2, H_2_-3), 2.14 (4H, dt, *J* = 8.1, 6.8 Hz, H_2_-7), 1.69 (4H, tt, *J* = 7.2, 6.8 Hz, H_2_-15), 1.58–1.48 (4H, m, H_2_-19), 1.31–1.20 (12H, m, H_2_-20, H_2_-21, H_2_-22); ^13^C NMR (DMSO-*d*_6_, 100 MHz) δ 172.3 (C-1), 171.2 (C-4), 144.7 (C-9), 128.4 (C-11), 127.6 (C-10), 126.1 (C-12), 47.9 (C-8), 46.8 (C-18), 44.5 (C-16), 37.3 (C-6) 35.4 (C-14), 34.6 (C-7), 30.6, 30.4 (C-2, C-3), 28.8, 28.5 (C-20 or C-21 or C-22), 26.1 (C-15), 25.9, 25.5 (C-19, C20 or C-21 or C-22); (+)-HRESIMS [M+H]^+^ *m*/*z* 875.6139 (calcd for C_54_H_79_N_6_O_4_, 875.6157).

##### *N*^1^,*N*^12^-Bis(3-(4-((3,3-diphenylpropyl)amino)-4-oxobutanamido)propyl)dodecane-1,12-diaminium 2,2,2-trifluroracetate (**16f**)

Following general procedure C, reaction of 4-((3,3-diphenylpropyl)amino)-4-oxobutanoic acid (**10**) (40 mg, 0.13 mmol) with di-*tert*-butyl dodecane-1,12-diylbis((3-aminopropyl)carbamate) (**5f**) (30 mg, 0.06 mmol), EDC·HCl (29 mg, 0.15 mmol), HOBt (20 mg, 0.15 mmol) and DIPEA (0.06 mL, 0.35 mmol) afforded di-*tert*-butyl dodecane-1,12-diylbis((3-(4-((3,3-diphenylpropyl)amino)-4-oxobutanamido)propyl)carbamate) as a colorless oil (34 mg, 53%). Following general procedure D, a sub-sample of this material (15 mg, 0.014 mmol) was reacted with TFA (0.2 mL) in CH_2_Cl_2_ (2 mL). Purification was achieved by C_8_ reversed-phase column chromatography (MeOH (+0.05% TFA):H_2_O (+0.05% TFA), 0:100→3:1) to afford the di-TFA salt **16f** as a colorless oil (15 mg, 97%). R*_f_* = 0.26 (RP-18, MeOH:10% aq. HCl, 5:1); IR (ATR) *v*_max_ 3450, 3288, 3062, 3028, 2928, 2855, 1644, 1550, 1200, 1175, 1129, 720, 699 cm^−1^; ^1^H NMR (DMSO-*d_6_*, 400 MHz) δ 8.43–8.33 (4H, m, NH_2_-17), 8.03 (2H, t, *J* = 5.8 Hz, NH-13), 7.88 (2H, t, *J* = 5.4 Hz, NH-5), 7.29–7.25 (16H, m, H-10, H-11), 7.19–7.14 (4H, m, H-12), 3.97 (2H, t, *J* = 7.8 Hz, H-8), 3.11 (4H, dt, *J* = 6.8, 6.2 Hz, H_2_-14), 2.96–2.89 (4H, m, H_2_-6), 2.89–2.84 (4H, m, H_2_-16), 2.84–2.77 (4H, m, H_2_-18), 2.34–2.26 (8H, m, H_2_-2, H_2_-3), 2.14 (4H, dt, *J* = 7.8, 7.0 Hz, H_2_-7), 1.69 (4H, tt, *J* = 7.1, 6.8 Hz, H_2_-15), 1.57–1.48 (4H, m, H_2_-19), 1.28–1.21 (16H, m, H_2_-20, H_2_-21, H_2_-22, H_2_-23); ^13^C NMR (DMSO-*d_6_*, 100 MHz) δ 172.3 (C-1), 171.2 (C-4), 144.7 (C-9), 128.4 (C-11), 127.6 (C-10), 126.1 (C-12), 47.9 (C-8), 46.8 (C-18), 44.4 (C-16), 37.3 (C-6), 35.4 (C-14), 34.6 (C-7), 30.6, 30.5 (C-2, C-3), 29.0, 28.8, 28.5 (C-20 or C-21 or C-22 or C-23), 26.1 (C-15), 25.9, 25.5 (C-19, C-20 or C-21 or C-22 or C-23); (+)-HRESIMS [M+2H]^2+^ *m*/*z* 451.3207 (calcd for C_56_H_82_N_6_O_4_, 451.3193).

##### *N*^1^,*N*^4^-Bis(3-(2,2,2-triphenylacetamido)propyl)butane-1,4-diaminium 2,2,2-trifluoroacetate (**17a**)

Following general procedure C, reaction of 2,2,2-triphenylacetic acid (**11**) (79 mg, 0.27 mmol) with di-*tert*-butyl butane 1,4-diylbis((3-aminopropyl)carbamate) (**5a**) (50 mg, 0.12 mmol), EDC·HCl (62 mg, 0.32 mmol), HOBt (44 mg, 0.32 mmol) and DIPEA (0.13 mL, 0.74 mmol) afforded di-*tert*-butyl butane-1,4-diylbis((3-(2,2,2-triphenylacetamido)propyl)carbamate) as a pale yellow oil (43 mg, 37%). Following general procedure D, a sub-sample of this material (20 mg, 0.021 mmol) was reacted with TFA (0.2 mL) in CH_2_Cl_2_ (2 mL). Purification was achieved by C_8_ reversed-phase column chromatography (MeOH (+0.05% TFA):H_2_O (+0.05% TFA), 0:100→3:1) to afford the di-TFA salt **17a** as a white oil (13 mg, 64%). R*_f_* = 0.46 (RP-18, MeOH:10% aq. HCl, 5:1); IR (ATR) *v*_max_ 3513, 3433, 3055, 2846, 2499, 1667, 1492, 1201, 1184, 1128, 721, 700 cm^−1^; ^1^H NMR (DMSO-*d_6_*, 400 MHz) δ 8.53–8.44 (4H, m, NH_2_-11), 7.42 (2H, t, *J* = 5.6 Hz, NH-7), 7.34–7.27 (12H, m, H-4/H-5/H-6), 7.27–7.17 (18H, m, H-4/H-5/H-6), 3.20 (4H, dt, *J* = 6.2, 6.0 Hz, H_2_-8), 2.83–2.74 (4H, m, H_2_-12), 2.67–2.58 (4H, m, H_2_-10), 1.70 (4H, tt, *J* = 7.0, 6.6 Hz, H_2_-9), 1.57–1.50 (4H, m, H_2_-13); ^13^C NMR (DMSO-*d_6_*, 100 MHz) δ 172.7 (C-1), 143.6 (C-3), 130.1, 127.7 (C-4, C-5), 126.5 (C-6), 67.3 (C-2), 46.0 (C-12), 44.5 (C-10), 36.6 (C-8), 25.6 (C-9), 22.6 (C-13); (+)-HRESIMS [M+H]^+^ *m*/*z* 743.4299 (calcd for C_50_H_55_N_4_O_2_, 743.4320).

##### *N*^1^,*N*^6^-Bis(3-(2,2,2-triphenylacetamido)propyl)hexane-1,6-diaminium 2,2,2-trifluoroacetate (**17b**)

Following general procedure C, reaction of 2,2,2-triphenylacetic acid (**11**) (74 mg, 0.26 mmol) with di-*tert*-butyl hexane-1,6-diylbis((3-aminopropyl)carbamate) (**5b**) (50 mg, 0.12 mmol), EDC·HCl (58 mg, 0.30 mmol), HOBt (41 mg, 0.30 mmol) and DIPEA (0.12 mL, 0.70 mmol) afforded di-*tert*-butyl hexane-1,6-diylbis((3-(2,2,2-triphenylacetamido)propyl)carbamate) as a colorless oil (45 mg, 40%). Following general procedure D, a sub-sample of this material (20 mg, 0.021 mmol) was reacted with TFA (0.2 mL) in CH_2_Cl_2_ (2 mL). Purification was achieved by C_8_ reversed-phase column chromatography (MeOH (+0.05% TFA):H_2_O (+0.05% TFA), 0:100→3:1) to afford the di-TFA salt **17b** as a colorless oil (14 mg, 67%). R*_f_* = 0.47 (RP-18, MeOH:10% aq. HCl, 9:1); IR (ATR) *v*_max_ 3495, 3384 3330, 3030, 2937, 2865, 2456, 2256, 2129, 1668, 1651, 1492, 1195, 1127, 1023, 996, 741, 717, 698 cm^−1^; ^1^H NMR (DMSO-*d_6_*, 400 MHz) δ 8.48–8.39 (4H, m, NH_2_-11), 7.42 (2H, t, *J* = 5.8 Hz, NH-7), 7.34–7.27 (12H, m, H-4/H-5/H-6), 7.27–7.20 (18H, m, H-4/H-5/H-6), 3.21 (4H, dt, *J* = 6.5, 6.2 Hz, H_2_-8), 2.81–2.71 (4H, m, H_2_-12), 2.67–2.57 (4H, m, H_2_-10), 1.70 (4H, tt, *J* = 7.4, 6.5 Hz, H_2_-9), 1.55–1.45 (4H, m, H_2_-13), 1.31–1.24 (4H, m, H_2_-14); ^13^C NMR (DMSO-*d_6_*, 100 MHz) δ 172.7 (C-1), 143.7 (C-3), 130.1, 127.7 (C-4, C-5), 126.5 (C-6), 67.3 (C-2), 46.6 (C-12), 44.6 (C-10), 36.6 (C-8), 25.6, 25.5, 25.3 (C-9, C-13, C-14); (+)-HRESIMS [M+H]^+^ *m*/*z* 771.4632 (calcd for C_52_H_59_N_4_O_2_, 771.4633).

##### *N*^1^,*N*^7^-Bis(3-(2,2,2-triphenylacetamido)propyl)heptane-1,7-diaminium 2,2,2-trifluoroacetate (**17c**)

Following general procedure C, reaction of 2,2,2-triphenylacetic acid (**11**) (71 mg, 0.25 mmol) with di-*tert*-butyl heptane-1,7-diylbis((3-aminopropyl)carbamate) (**5c**) (50 mg, 0.11 mmol), EDC·HCl (56 mg, 0.29 mmol), HOBt (39 mg, 0.29 mmol) and DIPEA (0.12 mL, 0.67 mmol) afforded di-*tert*-butyl heptane-1,7-diylbis((3-(2,2,2-triphenylacetamido)propyl)carbamate) as a colorless oil (50 mg, 45%). Following general procedure D, a sub-sample of this material (20 mg, 0.020 mmol) was reacted with TFA (0.2 mL) in CH_2_Cl_2_ (2 mL). Purification was achieved by C_8_ reversed-phase column chromatography (MeOH (+0.05% TFA):H_2_O (+0.05% TFA), 0:100→3:1) to afford the di-TFA salt **17c** as a whiteish oil (13 mg, 64%). R*_f_* = 0.47 (RP-18, MeOH:10% aq. HCl, 9:1); IR (ATR) *v*_max_ 3452, 3360, 3029, 2939, 2859, 2492, 1670, 1492, 1446, 1200, 1176, 1130, 700 cm^−1^; ^1^H NMR (DMSO-*d_6_*, 400 MHz) δ 8.41–8.32 (4H, m, NH_2_-11), 7.42 (2H, t, *J* = 5.9 Hz, NH-7), 7.34–7.27 (12H, m, H-4/H-5/H-6), 7.27–7.19 (18H, m, H-4/H-5/H-6), 3.21 (4H, dt, *J* = 6.4, 6.2 Hz, H_2_-8), 2.80–2.71 (4H, m, H_2_-12), 2.66–2.57 (4H, m, H_2_-10), 1.69 (4H, tt, *J* = 7.4, 6.8 Hz, H_2_-9), 1.55–1.45 (4H, m, H_2_-13), 1.31–1.22 (6H, m, H_2_-14, H_2_-15); ^13^C NMR (DMSO-*d_6_*, 100 MHz) δ 172.8 (C-1), 143.7 (C-3), 130.1, 127.7 (C-4, C-5), 126.5 (C-6), 67.3 (C-2), 46.6 (C-12), 44.5 (C-10), 36.6 (C-8), 28.0, 25.7 (C-14, C-15), 25.6, 25.3 (C-9, C-13); (+)-HRESIMS [M+H]^+^ *m*/*z* 785.4765 (calcd for C_53_H_61_N_4_O_2_, 785.4789).

##### *N*^1^,*N*^8^-Bis(3-(2,2,2-triphenylacetamido)propyl)octane-1,8-diaminium 2,2,2-trifluoroacetate (**17d**)

Following general procedure C, reaction of 2,2,2-triphenylacetic acid (**11**) (69 mg, 0.24 mmol) with di-*tert*-butyl octane-1,8-diylbis((3-aminopropyl)carbamate) (**5d**) (50 mg, 0.11 mmol), EDC·HCl (54 mg, 0.28 mmol), HOBt (38 mg, 0.28 mmol) and DIPEA (0.11 mL, 0.65 mmol) afforded di-*tert*-butyl octane-1,8-diylbis((3-(2,2,2-triphenylacetamido)propyl)carbamate) as a colorless oil (54 mg, 50%). Following general procedure D, a sub-sample of this material (20 mg, 0.020 mmol) was reacted with TFA (0.2 mL) in CH_2_Cl_2_ (2 mL). Purification was achieved by C_8_ reversed-phase column chromatography (MeOH (+0.05% TFA):H_2_O (+0.05% TFA), 0:100→3:1) to afford the di-TFA salt **17d** as a whiteish oil (12 mg, 59%). R*_f_* = 0.43 (RP-18, MeOH:10% aq. HCl, 9:1); IR (ATR) *v*_max_ 3433, 3335, 3030, 2936, 2858, 2502, 1673, 1652, 1492, 1199, 1177, 1129, 719, 699 cm^−1^; ^1^H NMR (DMSO-*d_6_*, 400 MHz) δ 8.38–8.28 (4H, m, NH_2_-11), 7.43 (2H, t, *J* = 6.0 Hz, NH-7), 7.34–7.27 (12H, m, H-4/H-5/H-6), 7.27–7.19 (18H, m, H-4/H-5/H-6), 3.21 (4H, dt, *J* = 6.4, 6.2 Hz, H_2_-8), 2.80–2.71 (4H, m, H_2_-12), 2.66–2.57 (4H, m, H_2_-10), 1.69 (4H, tt, *J* = 7.2, 6.5 Hz, H_2_-9), 1.54–1.44 (4H, m, H_2_-13), 1.32–1.21 (8H, m, H_2_-14, H_2_-15); ^13^C NMR (DMSO-*d_6_*, 100 MHz) δ 172.8 (C-1), 143.7 (C-3), 130.1, 127.7 (C-4, C-5), 126.5 (C-6), 67.3 (C-2), 46.7 (C-12), 44.5 (C-10), 36.6 (C-8), 28.3, 25.8 (C-14, C-15), 25.7, 25.4 (C-9, C-13); (+)-HRESIMS [M+H]^+^ *m*/*z* 799.4914 (calcd for C_54_H_63_N_4_O_2_, 799.4946).

##### *N*^1^,*N*^10^-Bis(3-(2,2,2-triphenylacetamido)propyl)decane-1,10-diaminium 2,2,2-trifluoroacetate (**17e**)

Following general procedure C, reaction of 2,2,2-triphenylacetic acid (**11**) (66 mg, 0.23 mmol) with di-*tert*-butyl decane-1,10-diylbis((3-aminopropyl)carbamate) (**5e**) (50 mg, 0.10 mmol), EDC·HCl (51 mg, 0.27 mmol), HOBt (36 mg, 0.27 mmol) and DIPEA (0.11 mL, 0.62 mmol) afforded di-*tert*-butyl decane-1,10-diylbis((3-(2,2,2-triphenylacetamido)propyl)carbamate) as a colorless oil (54 mg, 51%). Following general procedure D, a sub-sample of this material (20 mg, 0.019 mmol) was reacted with TFA (0.2 mL) in CH_2_Cl_2_ (2 mL). Purification was achieved by C_8_ reversed-phase column chromatography (MeOH (+0.05% TFA):H_2_O (+0.05% TFA), 0:100→3:1) to afford the di-TFA salt **17e** as a colorless oil (19 mg, 95%). R*_f_* = 0.46 (RP-18, MeOH:10% aq. HCl, 9:1); IR (ATR) *v*_max_ 3430, 3345, 3030, 2931, 2856, 2502, 1673, 1652, 1492, 1199, 1175, 1130, 719, 700 cm^−1^; ^1^H NMR (DMSO-*d_6_*, 400 MHz) δ 8.47–8.37 (4H, m, NH_2_-11), 7.42 (2H, t, *J* = 6.0 Hz, NH-7), 7.33–7.27 (12H, m, H-4/H-5/H-6), 7.26–7.20 (18H, m, H-4/H-5/H-6), 3.20 (4H, dt, *J* = 6.4, 6.2 Hz, H_2_-8), 2.79–2.72 (4H, m, H_2_-12), 2.65–2.58 (4H, m, H_2_-10), 1.70 (4H, tt, *J* = 7.2, 6.4 Hz, H_2_-9), 1.54–1.46 (4H, m, H_2_-13), 1.33–1.21 (12H, m, H_2_-14, H_2_-15, H_2_-16); ^13^C NMR (DMSO-*d_6_*, 100 MHz) δ 172.7 (C-1), 143.7 (C-3), 130.1, 127.7 (C-4, C-5), 126.5 (C-6), 67.3 (C-2), 46.7 (C-12), 44.5 (C-10), 36.6 (C-8), 28.7, 28.5, 25.9 (C-14, C-15, C-16), 25.6, 25.4 (C-9, C-13); (+)-HRESIMS [M+H]^+^ *m*/*z* 827.5245 (calcd for C_56_H_67_N_4_O_2_, 827.5259).

##### *N*^1^,*N*^12^-Bis(3-(2,2,2-triphenylacetamido)propyl)dodecane-1,12-diaminium 2,2,2-trifluoroacetate (**17f**)

Following general procedure C, reaction of 2,2,2-triphenylacetic acid (**11**) (61 mg, 0.21 mmol) with di-*tert*-butyl dodecane-1,12-diylbis((3-aminopropyl)carbamate) (**5f**) (50 mg, 0.10 mmol), EDC·HCl (48 mg, 0.25 mmol), HOBt (34 mg, 0.25 mmol) and DIPEA (0.10 mL, 0.58 mmol) afforded di-*tert*-butyl dodecane-1,12-diylbis((3-(2,2,2-triphenylacetamido)propyl)carbamate) as a colorless oil (46 mg, 45%). Following general procedure D, a sub-sample of this material (20 mg, 0.019 mmol) was reacted with TFA (0.2 mL) in CH_2_Cl_2_ (2 mL). Purification was achieved by C_8_ reversed-phase column chromatography (MeOH (+0.05% TFA):H_2_O (+0.05% TFA), 0:100→3:1) to afford the di-TFA salt **17f** as a whiteish oil (5 mg, 24%). R*_f_* = 0.37 (RP-18, MeOH:10% aq. HCl, 9:1); IR (ATR) *v*_max_ 3357, 3018, 2928, 2855, 1672, 1492, 1201, 1176, 1131, 720 cm^−1^; ^1^H NMR (DMSO-*d_6_*, 400 MHz) δ 8.26–8.16 (4H, m, NH_2_-11), 7.43 (2H, t, *J* = 5.9 Hz, NH-7), 7.34–7.27 (12H, m, H-4/H-5/H-6), 7.27–7.19 (18H, m, H-4/H-5/H-6), 3.20 (4H, dt, *J* = 6.3, 6.1 Hz, H_2_-8), 2.79–2.70 (4H, m, H_2_-12), 2.65–2.56 (4H, m, H_2_-10), 1.68 (4H, tt, *J* = 7.4, 6.2 Hz, H_2_-9), 1.54–1.43 (4H, m, H_2_-13), 1.32–1.21 (16H, m, H_2_-14, H_2_-15, H_2_-16, H_2_-17); ^13^C NMR (DMSO-*d_6_*, 100 MHz) δ 172.8 (C-1), 143.7 (C-3), 130.1, 127.7 (C-4, C-5), 126.5 (C-6), 67.3 (C-2), 46.7 (C-12), 44.5 (C-10), 36.5 (C-8), 29.0, 28.9, 28.5 (C-14 or C-15 or C-16 or C-17), 25.9, 25.7, 25.4 (C-9, C-13, C-14 or C-15 or C-16 or C-17); (+)-HRESIMS [M+H]^+^ *m*/*z* 855.5545 (calcd for C_58_H_71_N_4_O_2_, 855.5572).

##### *N*^1^,*N*^4^-Bis(3-(3,3,3-triphenylpropanamido)propyl)butane-1,4-diaminium 2,2,2-trifluoroacetate (**18a**)

Following general procedure C, reaction of 3,3,3-triphenylpropionic acid (**12**) (83 mg, 0.27 mmol) with di-*tert*-butyl butane 1,4-diylbis((3-aminopropyl)carbamate) (**5a**) (50 mg, 0.12 mmol), EDC·HCl (62 mg, 0.32 mmol), HOBt (44 mg, 0.32 mmol) and DIPEA (0.13 mL, 0.74 mmol) afforded di-*tert*-butyl butane-1,4-diylbis((3-(3,3,3-triphenylpropanamido)propyl)carbamate) as a colorless oil (39 mg, 32%). Following general procedure D, a sub-sample of this material (15 mg, 0.015 mmol) was reacted with TFA (0.2 mL) in CH_2_Cl_2_ (2 mL). Purification was achieved by C_8_ reversed-phase column chromatography (MeOH (+0.05% TFA):H_2_O (+0.05% TFA), 0:100→3:1) to afford the di-TFA salt **18a** as a colorless oil (12 mg, 80%). R*_f_* = 0.46 (RP-18, MeOH:10% aq. HCl, 9:1); IR (ATR) *v*_max_ 3420, 3288, 3057, 2827, 2505, 1669, 1645, 1199, 1177, 1127, 720, 699 cm^−1^; ^1^H NMR (DMSO-*d_6_*, 400 MHz) δ 8.44–8.34 (4H, m, NH_2_-12), 7.87 (2H, t, *J* = 5.8 Hz, NH-8), 7.27–7.22 (12H, m, H-5/H-6/H-7), 7.22–7.15 (18H, m, H-5/H-6/H-7), 3.62 (4H, br s, H_2_-2), 2.88 (4H, dt, *J* = 6.4, 6.2 Hz, H_2_-9), 2.74–2.65 (4H, m, H_2_-13), 2.50–2.45 (4H, m, H_2_-11), 1.53–1.47 (4H, m, H_2_-14), 1.44 (4H, tt, *J* = 6.9, 6.9 Hz, H_2_-10); ^13^C NMR (DMSO-*d_6_*, 100 MHz) δ 170.6 (C-1), 147.2 (C-4), 129.2, 127.5 (C-5, C-6), 125.8 (C-7), 55.7 (C-3), 46.5 (C-2), 46.1 (C-13), 44.3 (C-11), 35.2 (C-9), 25.9 (C-10), 22.5 (C-14); (+)-HRESIMS [M+H]^+^ *m*/*z* 771.4634 (calcd for C_52_H_59_N_4_O_2_, 771.4633).

##### *N*^1^,*N*^6^-Bis(3-(3,3,3-triphenylpropanamido)propyl)hexane-1,6-diaminium 2,2,2-trifluoroacetate (**18b**)

Following general procedure C, reaction of 3,3,3-triphenylpropionic acid (**12**) (77 mg, 0.26 mmol) with di-*tert*-butyl hexane-1,6-diylbis((3-aminopropyl)carbamate) (**5b**) (50 mg, 0.12 mmol), EDC·HCl (58 mg, 0.30 mmol), HOBt (41 mg, 0.30 mmol) and DIPEA (0.12 mL, 0.70 mmol) afforded di-*tert*-butyl hexane-1,6-diylbis((3-(3,3,3-triphenylpropanamido)propyl)carbamate) as a colorless oil (64 mg, 55%). Following general procedure D, a sub-sample of this material (20 mg, 0.020 mmol) was reacted with TFA (0.2 mL) in CH_2_Cl_2_ (2 mL). Purification was achieved by C_8_ reversed-phase column chromatography (MeOH (+0.05% TFA):H_2_O (+0.05% TFA), 0:100→3:1) to afford the di-TFA salt **18b** as a colorless oil (14 mg, 68%). R*_f_* = 0.49 (RP-18, MeOH:10% aq. HCl, 9:1); IR (ATR) *v*_max_ 3423, 3240, 3071, 2928, 2756, 1676, 1637, 1466, 1181, 1131, 702 cm^−1^; ^1^H NMR (DMSO-*d_6_*, 400 MHz) δ 8.34–8.25 (4H, m, NH_2_-12), 7.89 (2H, t, *J* = 5.8 Hz, NH-8), 7.27–7.22 (12H, m, H-5/H-6/H-7), 7.22–7.15 (18H, m, H-5/H-6/H-7), 3.63 (4H, br s, H_2_-2), 2.89 (4H, dt, *J* = 6.3, 6.1 Hz, H_2_-9), 2.70–2.64 (4H, m, H_2_-13), 2.48–2.42 (4H, m, H_2_-11), 1.51–1.45 (4H, m, H_2_-14), 1.45–1.40 (4H, m, H_2_-10), 1.27–1.21 (4H, m, H_2_-15); ^13^C NMR (DMSO-*d_6_*, 100 MHz) δ 170.7 (C-1), 147.2 (C-4), 129.2, 127.5 (C-5, C-6), 125.8 (C-7), 55.7 (C-3), 46.7 (C-13), 46.5 (C-2), 44.2 (C-11), 35.2 (C-9), 26.0 (C-10), 25.5, 25.3 (C-14, C-15); (+)-HRESIMS [M+H]^+^ *m*/*z* 799.4946 (calcd for C_54_H_63_N_4_O_2_, 799.4946).

##### *N*^1^,*N*^7^-Bis(3-(3,3,3-triphenylpropanamido)propyl)heptane-1,7-diaminium 2,2,2-trifluoroacetate (**18c**)

Following general procedure C, reaction of 3,3,3-triphenylpropionic acid (**12**) (75 mg, 0.25 mmol) with di-*tert*-butyl heptane-1,7-diylbis((3-aminopropyl)carbamate) (**5c**) (50 mg, 0.11 mmol), EDC·HCl (56 mg, 0.29 mmol), HOBt (39 mg, 0.29 mmol) and DIPEA (0.12 mL, 0.67 mmol) afforded di-*tert*-butyl heptane-1,7-diylbis((3-(3,3,3-triphenylpropanamido)propyl)carbamate) as a colorless oil (69 mg, 61%). Following general procedure D, a sub-sample of this material (20 mg, 0.020 mmol) was reacted with TFA (0.2 mL) in CH_2_Cl_2_ (2 mL). Purification was achieved by C_8_ reversed-phase column chromatography (MeOH (+0.05% TFA):H_2_O (+0.05% TFA), 0:100→3:1) to afford the di-TFA salt **18c** as a colorless oil (16 mg, 77%). R*_f_* = 0.49 (RP-18, MeOH:10% aq. HCl, 9:1); IR (ATR) *v*_max_ 3405, 3288, 3058, 2939, 2859, 2505, 1670, 1645, 1199, 1175, 1126, 719, 699 cm^−1^; ^1^H NMR (DMSO-*d_6_*, 400 MHz) δ 8.34–8.24 (4H, m, NH_2_-12), 7.90 (2H, t, *J* = 5.8 Hz, NH-8), 7.27–7.22 (12H, m, H-5/H-6/H-7), 7.22–7.15 (18H, m, H-5/H-6/H-7), 3.63 (4H, br s, H_2_-2), 2.89 (4H, dt, *J* = 6.4, 6.2 Hz, H_2_-9), 2.70–2.64 (4H, m, H_2_-13), 2.48–2.42 (4H, m, H_2_-11), 1.52–1.46 (4H, m, H_2_-14), 1.45–1.40 (4H, m, H_2_-10), 1.28–1.20 (6H, m, H_2_-15, H_2_-16); ^13^C NMR (DMSO-*d_6_*, 100 MHz) δ 170.7 (C-1), 147.2 (C-4), 129.2, 127.4 (C-5, C-6), 125.8 (C-7), 55.7 (C-3), 46.7 (C-13), 46.5 (C-2), 44.2 (C-11), 35.2 (C-9), 28.0 (C-15 or C-16), 26.0 (C-10), 25.7, 25.4 (C-14, C-15 or C-16); (+)-HRESIMS [M+H]^+^ *m*/*z* 813.5101 (calcd for C_55_H_65_N_4_O_2_, 813.5102).

##### *N*^1^,*N*^8^-Bis(3-(3,3,3-triphenylpropanamido)propyl)octane-1,8-diaminium 2,2,2-trifluoroacetate (**18d**)

Following general procedure C, reaction of 3,3,3-triphenylpropionic acid (**12**) (73 mg, 0.24 mmol) with di-*tert*-butyl octane-1,8-diylbis((3-aminopropyl)carbamate) (**5d**) (50 mg, 0.11 mmol), EDC·HCl (54 mg, 0.28 mmol), HOBt (38 mg, 0.28 mmol) and DIPEA (0.11 mL, 0.65 mmol) afforded di-*tert*-butyl octane-1,8-diylbis((3-(3,3,3-triphenylpropanamido)propyl)carbamate) as a colorless oil (62 mg, 55%). Following general procedure D, a sub-sample of this material (20 mg, 0.019 mmol) was reacted with TFA (0.2 mL) in CH_2_Cl_2_ (2 mL). Purification was achieved by C_8_ reversed-phase column chromatography (MeOH (+0.05% TFA):H_2_O (+0.05% TFA), 0:100→3:1) to afford the di-TFA salt **18d** as a colorless oil (10 mg, 50%). R*_f_* = 0.49 (RP-18, MeOH:10% aq. HCl, 9:1); IR (ATR) *v*_max_ 3455, 3287, 3058, 2933, 2857, 1673, 1446, 1199, 1175, 1127, 719, 699 cm^−1^; ^1^H NMR (DMSO-*d_6_*, 400 MHz) δ 8.30–8.20 (4H, m, NH_2_-12), 7.90 (2H, t, *J* = 5.8 Hz, NH-8), 7.28–7.22 (12H, m, H-6), 7.22–7.15 (18H, m, H-5, H-7), 3.63 (4H, br s, H_2_-2), 2.89 (4H, dt, *J* = 6.4, 6.2 Hz, H_2_-9), 2.70–2.63 (4H, m, H_2_-13), 2.47–2.41 (4H, m, H_2_-11), 1.51–1.46 (4H, m, H_2_-14), 1.46–1.39 (4H, m, H_2_-10), 1.28–1.19 (8H, m, H_2_-15, H_2_-16); ^13^C NMR (DMSO-*d_6_*, 100 MHz) δ 170.7 (C-1), 147.2 (C-4). 129.2, 127.4 (C-5, C-6), 125.8 (C-7), 55.7 (C-3), 46.8 (C-13), 46.5 (C-2), 44.2 (C-11), 35.1 (C-9), 28.3 (C-15 or C-16), 26.0 (C-10), 25.8, 25.4 (C-14, C-15 or C-16); (+)-HRESIMS [M+H]^+^ *m*/*z* 827.5265 (calcd for C_56_H_67_N_4_O_2_, 827.5259).

##### *N*^1^,*N*^10^-Bis(3-(3,3,3-triphenylpropanamido)propyl)decane-1,10-diaminium 2,2,2-trifluoroacetate (**18e**)

Following general procedure C, reaction of 3,3,3-triphenylpropionic acid (**12**) (69 mg, 0.23 mmol) with di-*tert*-butyl decane-1,10-diylbis((3-aminopropyl)carbamate) (**5e**) (50 mg, 0.10 mmol), EDC·HCl (51 mg, 0.27 mmol), HOBt (36 mg, 0.27 mmol) and DIPEA (0.11 mL, 0.62 mmol) afforded di-*tert*-butyl decane-1,10-diylbis((3-(3,3,3-triphenylpropanamido)propyl)carbamate) as a colorless oil (33 mg, 30%). Following general procedure D, a sub-sample of this material (15 mg, 0.014 mmol) was reacted with TFA (0.2 mL) in CH_2_Cl_2_ (2 mL). Purification was achieved by C_8_ reversed-phase column chromatography (MeOH (+0.05% TFA):H_2_O (+0.05% TFA), 0:100→3:1) to afford the di-TFA salt **18e** as a colorless oil (10 mg, 66%). R*_f_* = 0.43 (RP-18, MeOH:10% aq. HCl, 9:1); IR (ATR) *v*_max_ 3251, 3068, 2925, 2861, 2473, 1676, 1640, 1443, 1199, 1172, 1128, 758, 720, 702 cm^−1^; ^1^H NMR (DMSO-*d_6_*, 400 MHz) δ 8.27–8.17 (4H, m, NH_2_-12), 7.90 (2H, t, *J* = 5.9 Hz, NH-8), 7.27–7.22 (12H, m, H-5/H-6/H-7), 7.22–7.15 (18H, m, H-5/H-6/H-7), 3.63 (4H, br s, H_2_-2), 2.89 (4H, dt, *J* = 6.4, 6.2 Hz, H_2_-9), 2.70–2.63 (4H, m, H_2_-13), 2.47–2.40 (4H, m, H_2_-11), 1.51–1.45 (4H, m, H_2_-14), 1.45–1.39 (4H, m, H_2_-10), 1.28–1.21 (12H, m, H_2_-15, H_2_-16, H_2_-17); ^13^C NMR (DMSO-*d_6_*, 100 MHz) δ 170.8 (C-1), 147.2 (C-4), 129.2, 127.4 (C-5, C-6), 125.8 (C-7), 55.7 (C-3), 46.8 (C-13), 46.5 (C-2), 44.2 (C-11), 35.1 (C-9), 28.7, 28.5 (C-15 or C-16 or C-17), 26.0 (C-10), 25.9, 25.4 (C-14, C-15 or C-16 or C-17); (+)-HRESIMS [M+H]^+^ *m*/*z* 855.5546 (calcd for C_58_H_71_N_4_O_2_, 855.5572).

##### *N*^1^,*N*^12^-Bis(3-(3,3,3-triphenylpropanamido)propyl)dodecane-1,12-diaminium 2,2,2-trifluoroacetate (**18f**)

Following general procedure C, reaction of 3,3,3-triphenylpropionic acid (**12**) (63 mg, 0.21 mmol) with di-*tert*-butyl dodecane-1,12-diylbis((3-aminopropyl)carbamate) (**5f**) (50 mg, 0.10 mmol), EDC·HCl (48 mg, 0.25 mmol), HOBt (34 mg, 0.25 mmol) and DIPEA (0.10 mL, 0.58 mmol) afforded di-*tert*-butyl dodecane-1,12-diylbis((3-(3,3,3-triphenylpropanamido)propyl)carbamate) as a colorless oil (48 mg, 46%). Following general procedure D, a sub-sample of this material (20 mg, 0.018 mmol) was reacted with TFA (0.2 mL) in CH_2_Cl_2_ (2 mL). Purification was achieved by C_8_ reversed-phase column chromatography (MeOH (+0.05% TFA):H_2_O (+0.05% TFA), 0:100→3:1) to afford the di-TFA salt **18f** as a colorless oil (13 mg, 65%). R*_f_* = 0.31 (RP-18, MeOH:10% aq. HCl, 9:1); IR (ATR) *v*_max_ 3276, 3100, 3057, 2926, 2854, 1667, 1641, 1199, 1174, 1130, 699 cm^−1^; ^1^H NMR (DMSO-*d_6_*, 400 MHz) δ 8.32–8.22 (4H, m, NH_2_-12), 7.90 (2H, t, *J* = 5.8 Hz, NH-8), 7.28–7.22 (12H, m, H-5/H-6/H-7), 7.22–7.14 (18H, m, H-5/H-6/H-7), 3.63 (4H, br s, H_2_-2), 2.89 (4H, dt, *J* = 6.4, 6.4 Hz, H_2_-9), 2.71–2.62 (4H, m, H_2_-13), 2.48–2.39 (4H, m, H_2_-11), 1.53–1.44 (4H, m, H_2_-14), 1.44–1.39 (4H, m, H_2_-10), 1.30–1.20 (16H, m, H_2_-15, H_2_-16, H_2_-17, H_2_-18); ^13^C NMR (DMSO-*d_6_*, 100 MHz) δ 170.7 (C-1), 147.2 (C-4), 129.2, 127.4 (C-5, C-6), 125.8 (C-7), 55.7 (C-3), 46.8 (C-13), 46.5 (C-2), 44.2 (C-11), 35.1 (C-9), 28.9, 28.8, 28.5 (C-15 or C-16 or C-17 or C-18), 26.0 (C-10), 25.9, 25.4 (C-14, C-15 or C-16 or C-17 or C-18); (+)-HRESIMS [M+H]^+^ *m*/*z* 883.5863 (calcd for C_60_H_75_N_4_O_2_, 883.5885).

### 3.3. Antimicrobial Assays

Antimicrobial evaluation against *Staphylococcus aureus* (ATCC 25923 or 29213), *S. aureus* (CF-Marseille) [34], *Bacillus cereus* (ATCC 11778) and *Pseudomonas aeruginosa* (ATCC 27853 or PAO1) was determined in microplates using the standard broth dilution method in accordance with the recommendations of the Comité de l’AntibioGramme de la Société Française de Microbiologie (CA-SFM). Briefly, the minimal inhibitory concentrations (MICs) were determined with an inoculum of 10^5^ CFU in 200 µL of Mueller–Hinton broth (MHB) containing two-fold serial dilutions of each drug. The MIC was defined as the lowest concentration of drug that completely inhibited visible growth after incubation for 18 h at 37 °C. To determine all MICs, the measurements were independently repeated in triplicate.

Additional antimicrobial evaluation against MRSA (ATCC 43300), *E. coli* (ATCC 25922), *Klebsiella pneumoniae* (ATCC 700603), *Acinetobacter baumannii* (ATCC 19606), *Candida albicans* (ATCC 90028) and *Cryptococcus neoformans* (ATCC 208821) was undertaken at the Community for Open Antimicrobial Drug Discovery at The University of Queensland (Australia) according to their standard protocols [36]. For antimicrobial assays, the tested strains were cultured in either Luria broth (LB) (In Vitro Technologies, USB75852), nutrient broth (NB) (Becton Dickson, 234000) or MHB at 37 °C overnight. A sample of culture was then diluted 40-fold in fresh MHB and incubated at 37 °C for 1.5−2 h. The compounds were serially diluted 2-fold across the wells of 96-well plates (Corning 3641, nonbinding surface), with compound concentrations ranging from 0.015 to 64 μg/ mL, plated in duplicate. The resultant mid log phase cultures were diluted to a final concentration of 1 × 10^6^ CFU/mL; then, 50 μL was added to each well of the compound-containing plates, giving a final compound concentration range of 0.008 to 32 μg/mL and cell density of 5 × 10^5^ CFU/mL. All plates were then covered and incubated at 37 °C for 18 h. Resazurin was added at 0.001% final concentration to each well and incubated for 2 h before MICs were read by eye.

For the antifungal assay, fungi strains were cultured for 3 days on YPD agar at 30 °C. A yeast suspension of 1 × 10^6^ to 5 × 10^6^ CFU/mL was prepared from five colonies. These stock suspensions were diluted with yeast nitrogen base (YNB) (Becton Dickinson, 233520) broth to a final concentration of 2.5 × 10^3^ CFU/mL. The compounds were serially diluted 2-fold across the wells of 96-well plates (Corning 3641, nonbinding surface), with compound concentrations ranging from 0.015 to 64 μg/mL and final volumes of 50 μL, plated in duplicate. Then, 50 μL of the fungi suspension that was previously prepared in YNB broth to a final concentration of 2.5 × 10^3^ CFU/mL was added to each well of the compound-containing plates, giving a final compound concentration range of 0.008 to 32 μg/mL. Plates were covered and incubated at 35 °C for 36 h without shaking. *C. albicans* MICs were determined by measuring the absorbance at OD_530_. For *C. neoformans*, resazurin was added at 0.006% final concentration to each well and incubated for a further 3 h before MICs were determined by measuring the absorbance at OD_570−600_.

Colistin and vancomycin were used as positive bacterial inhibitor standards for Gram-negative and Gram-positive bacteria, respectively. Fluconazole was used as a positive fungal inhibitor standard for *C. albicans* and *C. neoformans*. The antibiotics were provided in 4 concentrations, with 2 above and 2 below its MIC value, and plated into the first 8 wells of column 23 of 384-well NBS plates. Quality control (QC) of the assays was determined by antimicrobial controls and Z’-factor (using positive and negative controls). Each plate was deemed to fulfil the quality criteria (pass QC) if the Z’-factor was above 0.4 and the antimicrobial standards showed full range of activity, with full growth inhibition at their highest concentration and no growth inhibition at their lowest concentration.

### 3.4. Determination of the MICs of Antibiotics in the Presence of Synergizing Compounds

Briefly, restoring enhancer concentrations were determined with an inoculum of 5 × 10^5^ CFU in 200 µL of MHB containing two-fold serial dilutions of each derivative in the presence of doxycycline at 2 µg/mL. The lowest concentration of the polyamine adjuvant that completely inhibited visible growth after incubation for 18 h at 37 °C was determined. These measurements were independently repeated in triplicate.

### 3.5. Cytotoxicity Assays

HEK293 cells were counted manually in a Neubauer hemocytometer and plated at a density of 5000 cells/well into each well of the 384-well plates containing the 25× (2 μL) concentrated compounds. The medium used was Dulbecco’s modified eagle medium (DMEM) supplemented with 10% fetal bovine serum (FBS). Cells were incubated together with the compounds for 20 h at 37 °C, 5% CO_2_. To measure cytotoxicity, 5 μL (equals 100 μM final) of resazurin was added to each well after incubation and incubated for further 3 h at 37 °C with 5% CO_2_. After final incubation, fluorescence intensity was measured as Fex 560/10 nm, em 590/10 nm (F_560/590_) using a Tecan M1000 Pro monochromator plate reader. CC_50_ values (concentration at 50% cytotoxicity) were calculated by normalizing the fluorescence readout, with 74 μg/mL tamoxifen as negative control (0%) and normal cell growth as positive control (100%). The concentration-dependent percentage cytotoxicity was fitted to a dose–response function (using Pipeline Pilot) and CC_50_ values determined.

### 3.6. Hemolytic Assays

Human whole blood was washed three times with 3 volumes of 0.9% NaCl and then resuspended in same to a concentration of 0.5 × 10^8^ cells/mL, as determined by manual cell count in a Neubauer hemocytometer. The washed cells were then added to the 384-well compound-containing plates for a final volume of 50 μL. After a 10 min shake on a plate shaker, the plates were then incubated for 1 h at 37 °C. After incubation, the plates were centrifuged at 1000× *g* for 10 min to pellet cells and debris; 25 μL of the supernatant was then transferred to a polystyrene 384-well assay plate. Hemolysis was determined by measuring the supernatant absorbance at 405 mm (OD_405_). The absorbance was measured using a Tecan M1000 Pro monochromator plate reader. HC_10_ and HC_50_ (concentration at 10% and 50% hemolysis, respectively) were calculated by curve fitting the inhibition values vs. log (concentration) using a sigmoidal dose–response function with variable fitting values for top, bottom and slope.

### 3.7. Real-Time Growth Curves

Solutions of compound **15d** at concentrations of 2, 4 and 16 µg/mL were tested, each in triplicate, against *S. aureus* ATCC 25923, MRSA (CF-Marseille) and *Bacillus cereus* ATCC 11778. Typically, in a 96-well plate, 10 µL of 40, 80 and 320 µg/mL stock solutions of compound **15d** were placed, as well as 190 µL of a 5 × 10^5^ CFU/mL of the selected bacterial suspension in brain heart infusion (BHI) broth. Positive controls containing only 200 µL of a 5 × 10^5^ CFU/mL of bacterial suspension in BHI and negative controls containing only 200 µL of BHI broth were added. The plate was incubated at 37 °C in a TECAN Spark Reader (Roche Diagnostic), and real-time bacterial growth was followed by OD_590_ nm measurement every 10 min during 19 h.

### 3.8. Minimum Bactericidal Concentration Test

A pure culture of a specified microorganism was grown overnight, then diluted in growth-supporting broth (typically Mueller–Hinton II broth) to a concentration between 1 × 10^5^ and 1 × 10^6^ CFU/mL. A stock dilution of the antimicrobial test compound was created at approximately 100 times the expected previously determined MIC. Further 1:1 dilution was made in 96-well microtiter plates. All dilutions of the test compound were inoculated with equal volumes of the specified microorganism (typically 100 µL). A positive and negative control tube or well is included to demonstrate adequate microbial growth over the course of the incubation period and media sterility, respectively. An aliquot of the positive control is plated and used to establish a baseline concentration of the microorganism used. The microtiter plates were then incubated at 37 °C for 24 h. Turbidity indicates growth of the microorganism, and the MIC is the lowest concentration where no growth is visually observed. To determine the minimum bactericidal concentration (MBC), the dilution representing the MIC and at least two of the more concentrated test product dilutions are plated on a solidified agar plate to determine the bacterial viability. The MBC is the lowest concentration where no growth is encountered when compared to the MIC dilution.

### 3.9. ATP Release Assay

Solutions of test compound **15d** were prepared in DMSO at various concentrations. A suspension of growing *S. aureus* to be studied in Muller–Hinton II broth was prepared and incubated at 37 °C. An aliquot (90 µL) of this suspension was added to 10 µL of test compound solution and vortexed for 10 s. Luciferin–luciferase reagent (Yelen, France; 50 µL) was immediately added to this mixture and luminescent signal quantified with an Infinite M200 microplate reader (Tecan) over a 30 min period. ATP concentration was quantified by internal sample addition. A similar procedure was used for the CTAB positive control.

## 4. Conclusions

In this study, α,ω-disubstituted polyamines exhibit promising antimicrobial properties and can also enhance action of other antibiotics towards drug-resistant Gram-negative bacteria. The present study explored variation in polyamine chain length, aromatic head group lipophilicity and linker chemistry on intrinsic antimicrobial and antibiotic enhancement biological activities. Favorable antimicrobial and antibiotic enhancement activities were observed for thiourea-linked examples, supporting previously reported interest in this class of polyamine derivative. The observation of cytotoxicity and/or hemolytic properties prompted investigation of alternative amide-linked alternatives. Of note was the discovery of diaryl-aromatic-head-group-substituted examples that exhibited growth inhibition of MRSA and *E. coli* with no cytotoxic or red blood cell hemolytic effects. Presence of diaryl substitution at each end of the polyamine chain was found to be optimal for antimicrobial selectivity, with mono-aryl examples being essentially inactive and triaryl variants being cytotoxic and/or hemolytic. While antibiotic enhancement was observed for the majority of the thiourea-linked examples, little to no enhancement was observed for the amide-linked analogues, highlighting the need for further research to define the attributes and influence of end group and linker chemistry on antimicrobial and antibiotic enhancement properties of substituted polyamines.

## Figures and Tables

**Scheme 2:**
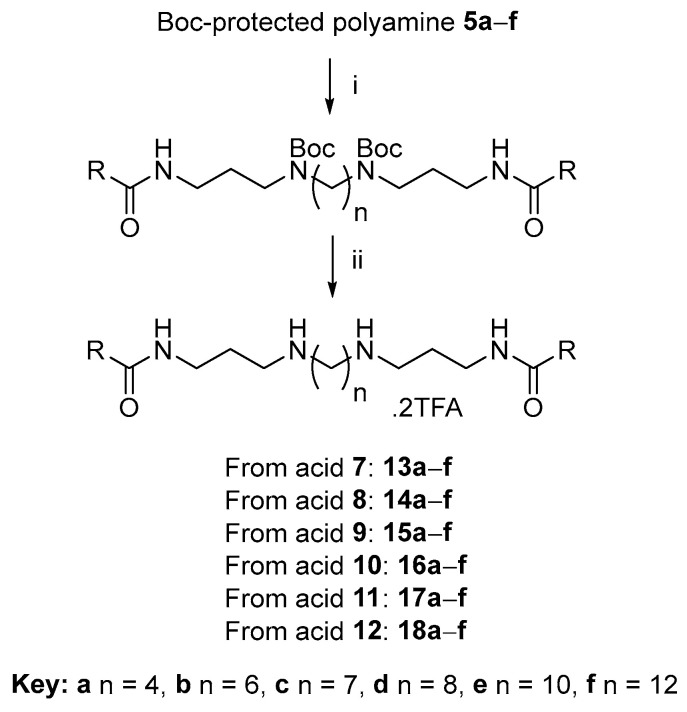
General method for synthesis of target polyamine analogues **13**–**18**. *Reagents and conditions*: (i) carboxylic acid RCO_2_H (**7**–**12**) (2.2 equiv.), Boc-protected polyamine (**5a**–**f**) (1 equiv.), EDC·HCl (2.6 equiv.) or EDC·HCl/HOBt (2.6 equiv.) or HBTU (2.5 equiv.), DIPEA (6 equiv.) or DMAP (2 equiv.), DMF or CH_2_Cl_2_, 0 °C, N_2_, 18 h (yields 12–84%); (ii) TFA (0.2 mL), CH_2_Cl_2_ (2 mL), r.t., 2 h (yields 24–100%).

**Table 1:** Antimicrobial and antifungal activities (MIC, μM) of analogues **6, 13**–**18**.

Cmpd	S. a ^a^	MRSA ^b^	Ec ^c^	P. a ^d^	K. p ^e^	A. b ^f^	C. a ^g^	C. n ^h^
**6a**	>276	≤0.34	2.8	138	>44.1	≤0.35	≤0.34	11.0
**6b**	>265	≤0.33	≤0.33	265	>42.4	10.7	≤0.33	>42.4
**6c**	65	≤0.33	≤0.33	>260	>41.7	2.6	≤0.33	0.65
**6d**	6.25	≤0.32	≤0.32	100	40.9	5.1	≤0.32	20.5
**6e**	>247	≤0.31	≤0.31	247	>39.5	4.9	≤0.31	>39.5
**6f**	>239	≤0.30	2.4	120	>38.2	>38.2	≤0.30	>38.2
**13a**	720	>46.1	>46.1	720	>46.1	>46.1	>46.1	>46.1
**13b**	>277	>44.3	>44.3	>277	>44.3	>44.3	>44.3	>44.3
**13c**	>271	>43.4	>43.4	>271	>43.4	>43.4	>43.4	>43.4
**13d**	>266	>42.6	>42.6	>266	>42.6	>42.6	>42.6	>42.6
**13e**	32	10.3	41.1	642	>41.1	>41.1	>41.1	41.1
**13f**	50	19.8	>39.7	300	>39.7	>39.7	>39.7	>39.7
**14a**	100	>37.0	>37.0	>200	>37.0	>37.0	>37.0	>37.0
**14b**	>200	>35.8	>35.8	>200	>35.8	>35.8	>35.8	>35.8
**14c**	200	>35.3	>35.3	>200	>35.3	>35.3	>35.3	>35.3
**14d**	>100	>34.7	>34.7	>100	>34.7	>34.7	>34.7	>34.7
**14e**	100	4.21	33.7	>200	>33.7	>33.7	>33.7	33.7
**14f**	6.25	≤0.26	>32.7	>100	>32.7	>32.7	>32.7	>32.7
**15a**	6.25	≤0.30	≤0.30	50	>37.8	>37.8	>37.8	>37.8
**15b**	12.5	≤0.29	4.6	100	>36.6	>36.6	>36.6	>36.6
**15c**	6.25	≤0.28	2.3	100	>36.0	>36.0	>36.0	≤0.28
**15d**	3.13	≤0.28	2.2	100	>35.4	>35.4	>35.4	>35.4
**15e**	3.13	≤0.27	≤0.27	200	>34.4	2.15	>34.4	>34.4
**15f**	1.56	≤0.26	2.1	200	>33.4	1.0	8.34	≤0.26
**16a**	25	≤0.25	>31.5	20	>31.5	>31.5	>31.5	≤0.25
**16b**	24	≤0.24	>30.6	480	>30.6	>30.6	>30.6	>30.6
**16c**	24	≤0.24	>30.2	480	>30.2	>30.2	>30.2	30.2
**16d**	12.5	≤0.23	>29.8	300	>29.8	>29.8	>29.8	>29.8
**16e**	12.5	≤0.23	14.5	200	29.1	29.1	29.1	≤0.23
**16f**	3.13	≤0.22	>28.3	300	>28.3	>28.3	>28.3	>28.3
**17a**	6.25	<0.26	1.03	300	>33.0	8.24	>33.0	<0.26
**17b**	6.25	<0.25	4.00	300	32.0	4.00	16.0	<0.25
**17c**	12.5	<0.25	7.90	300	31.6	3.95	15.8	15.8
**17d**	6.25	<0.24	15.6	300	>31.2	7.79	15.6	<0.24
**17e**	3.125	<0.23	<0.23	300	>30.3	>30.3	>30.3	<0.23
**17f**	6.25	<0.23	>29.5	300	>29.5	>29.5	<0.23	<0.23
**18a**	3.125	<0.25	0.50	25	32.0	4.00	8.00	32.0
**18b**	1.56	<0.24	0.97	50	31.2	7.8	7.79	31.2
**18c**	1.56	<0.24	3.84	100	30.7	1.92	3.84	30.7
**18d**	3.125	<0.24	7.58	200	>30.3	7.6	<0.24	7.6
**18e**	1.56	<0.23	>29.5	200	>29.5	<0.23	<0.23	<0.23
**18f**	25	<0.22	>28.8	300	>28.8	>28.8	<0.23	<0.23

^a^ *Staphylococcus aureus* ATCC 25923 or ATCC 29213 with streptomycin (MIC 21.5 μM) and chloramphenicol (MIC 1.5–3 μM) used as positive controls and values presented as the mean (*n* = 3); ^b^ MRSA ATCC 43300 with vancomycin (MIC 0.7 μM) used as a positive control and values presented as the mean (*n* = 2); ^c^ *Escherichia coli* ATCC 25922 with colistin (MIC 0.1 μM) used as positive control and values presented as the mean (*n* = 2); ^d^ *Pseudomonas aeruginosa* PAO1 or ATCC 27853 with streptomycin (MIC 21.5 μM) and colistin (MIC 1 μM) used as positive controls and values presented as the mean (*n* = 3); ^e^ *Klebsiella pneumoniae* ATCC700603 with colistin (MIC 0.2 μM) as a positive control and values presented as the mean (*n* = 2); ^f^ *Acinetobacter baumannii* ATCC19606 with colistin (MIC 0.2 μM) as a positive control and values presented as the mean (*n* = 2); ^g^ *Candida albicans* ATCC90028 with fluconazole (MIC 0.4 μM) as a positive control and values presented as the mean (*n* = 2); ^h^ *Cryptococcus neoformans* ATCC208821 with fluconazole (MIC 26 μM) as a positive control and values presented as the mean (*n* = 2).

**Table 2:** Cytotoxic and hemolytic activity of analogues **6**, **13**–**18**.

Compound	Cytotoxicity ^a^	Hemolysis ^b^	cLogP ^c^	Compound	Cytotoxicity ^a^	Hemolysis ^b^	cLogP ^c^
**6a**	>44.1	7.73	6.3	**16a**	>31.5	>31.5	6.6
**6b**	>42.4	2.96	7.3	**16b**	>30.6	>30.6	7.6
**6c**	>41.7	≤0.326	7.7	**16c**	>30.2	>30.2	8.0
**6d**	10.12	2.07	8.2	**16d**	>29.8	>29.8	8.5
**6e**	>39.5	>39.5	9.1	**16e**	17.3	17.9	9.4
**6f**	≤0.298	>38.2	10.0	**16f**	>28.3	3.17	10.3
**13a**	nt ^d^	nt ^d^	3.6	**17a**	0.329	5.57	7.8
**13b**	>44.3	>44.3	4.5	**17b**	≤0.250	6.27	8.7
**13c**	>43.4	>43.4	5.0	**17c**	≤0.247	8.16	9.2
**13d**	>42.6	>42.6	5.4	**17d**	≤0.243	0.719	9.6
**13e**	>41.1	>41.1	6.3	**17e**	>30.3	≤0.234	10.5
**13f**	>39.7	>39.7	7.3	**17f**	≤0.231	≤0.231	11.4
**14a**	>37	>37	3.6	**18a**	18.3	1.58	8.7
**14b**	nt ^d^	nt ^d^	4.5	**18b**	15.7	0.985	9.6
**14c**	nt ^d^	nt ^d^	5.0	**18c**	13.2	3.12	10.1
**14d**	>34.7	>34.7	5.4	**18d**	9.70	3.17	10.5
**14e**	>33.7	>33.7	6.3	**18e**	7.23	≤0.231	11.4
**14f**	>32.7	>32.7	7.3	**18f**	0.409	≤0.225	12.4
**15a**	>37.8	>37.8	6.6				
**15b**	>36.6	>36.6	7.6				
**15c**	>36.0	>36.0	8.0				
**15d**	>35.4	>35.4	8.5				
**15e**	>34.4	>34.4	9.4				
**15f**	5.46	1.17	10.3				

^a^ Concentration of compound at 50% cytotoxicity on HEK293 human embryonic kidney cells and values presented as the mean (*n* = 2). Highest dose tested was 32 μg/mL. Tamoxifen was the positive control (IC_50_ 24 μM); ^b^ concentration (HC_10_, μM) of compound at 10% hemolytic activity on human red blood cells and values presented as the mean (*n* = 2). Melittin was the positive control (HC_10_ 0.95 μM); ^c^ cLogP values calculated in DataWarrior v05.05.00 [32]; ^d^ not tested.

**Table 3:** Doxycycline potentiation activity of analogues **6, 13**–**18**.

Compound	Conc (µM) for Potentiation ^a^	Compound	Conc (µM) for Potentiation ^a^
**6a**	8.6	**16a**	25
**6b**	16.6	**16b**	96
**6c**	8.14	**16c**	47
**6d**	50	**16d**	100
**6e**	15.4	**16e**	100
**6f**	>120	**16f**	300
**13a**	720	**17a**	300
**13b**	280	**17b**	300
**13c**	68	**17c**	300
**13d**	270	**17d**	300
**13e**	260	**17e**	300
**13f**	300	**17f**	300
**14a**	50	**18a**	12.5
**14b**	200	**18b**	25
**14c**	100	**18c**	50
**14d**	>100	**18d**	200
**14e**	200	**18e**	200
**14f**	50	**18f**	300
**15a**	12.5		
**15b**	50		
**15c**	50		
**15d**	50		
**15e**	100		
**15f**	100		

^a^ Concentration (µM) required to restore doxycycline activity at 2 μg/mL (4.5 µM) against *P. aeruginosa* ATCC27853.

## Data Availability

Data are contained within the article or Supplementary Materials.

## Supplementary Materials

The supporting information can be downloaded at: https://www.mdpi.com/article/10.3390/ijms24065882/s1.
