# Supplementary material for: α,ω-Diacyl-Substituted Analogues of Natural and Unnatural Polyamines: Identification of Potent Bactericides That Selectively Target Bacterial Membranes
Source: Int J Mol Sci. 2023 Mar 20;24(6):5882. doi: 10.3390/ijms24065882 (PMC10052977; doi:10.3390/ijms24065882)
Supplement: Supplementary file 1 [file ijms-24-05882-s001.zip › ijms-2281200-supplementary.pdf]

Supporting Information

α,ω-Diacyl-substituted analogues of natural and unnatural polyamines: identification of potent bactericides that selectively target bacterial membranes

Dan Chen [1], Melissa M. Cadelis [1], Florent Rouvier [2], Thomas Troia [2], Liam R. Edmeades [1], Kyle Fraser [1], Evangelene S. Gill [1], Marie-Lise Bourguet-Kondracki [3], Jean Michel Brunel [2], and Brent R. Copp [1,*]

[1] School of Chemical Sciences, The University of Auckland, Private Bag 92019, Auckland 1142, New Zealand

[2] UMR MD1 "Membranes et Cibles Thérapeutiques", U1261 INSERM, Faculté de Pharmacie, Aix-Marseille Universite, 27 bd Jean Moulin, 13385 Marseille, France

[3] Laboratoire Molécules de Communication et Adaptation des Micro-organismes, UMR 7245 CNRS, Muséum National d'Histoire Naturelle, 57 rue Cuvier (C.P. 54), 75005 Paris, France

*Corresponding author. Brent R. Copp (b.copp@auckland.ac.nz)

Table of Figures

Figure **S1** ^{1}H NMR (DMSO-d_6, 400 MHz) and ^{13}C NMR (DMSO-d_6, 100 MHz) spectra for **6a**. 4
Figure **S2** ^{1}H NMR (DMSO-d_6, 400 MHz) and ^{13}C NMR (DMSO-d_6, 100 MHz) spectra for **6b**. 5
Figure **S3** ^{1}H NMR (DMSO-d_6, 400 MHz) and ^{13}C NMR (DMSO-d_6, 100 MHz) spectra for **6c**. 6
Figure **S4** ^{1}H NMR (DMSO-d_6, 400 MHz) and ^{13}C NMR (DMSO-d_6, 100 MHz) spectra for **6d**. 7
Figure **S5** ^{1}H NMR (DMSO-d_6, 400 MHz) and ^{13}C NMR (DMSO-d_6, 100 MHz) spectra for **6e**. 8
Figure **S6** ^{1}H NMR (DMSO-d_6, 400 MHz) and ^{13}C NMR (DMSO-d_6, 100 MHz) spectra for **6f**. 9
Figure **S7** ^{1}H NMR (DMSO-d_6, 400 MHz) and ^{13}C NMR (DMSO-d_6, 100 MHz) spectra for **8**. 10
Figure **S8** ^{1}H NMR (DMSO-d_6, 400 MHz) and ^{13}C NMR (DMSO-d_6, 100 MHz) spectra for **10**. 11
Figure **S9** ^{1}H NMR (CD_3OD, 400 MHz) and ^{13}C NMR (CD_3OD, 100 MHz) spectra for **13a**. 12
Figure **S10** ^{1}H NMR (CD_3OD, 400 MHz) and ^{13}C NMR (CD_3OD, 100 MHz) spectra for **13b**. 13
Figure **S11** ^{1}H NMR (CD_3OD, 400 MHz) and ^{13}C NMR (CD_3OD, 100 MHz) spectra for **13c**. 14
Figure **S12** ^{1}H NMR (CD_3OD, 400 MHz) and ^{13}C NMR (CD_3OD, 100 MHz) spectra for **13d**. 15
Figure **S13** ^{1}H NMR (CD_3OD, 400 MHz) and ^{13}C NMR (CD_3OD, 100 MHz) spectra for **13e**. 16
Figure **S14** ^{1}H NMR (CD_3OD, 400 MHz) and ^{13}C NMR (CD_3OD, 100 MHz) spectra for **13f**. 17
Figure **S15** ^{1}H NMR (DMSO-d_6, 400 MHz) and ^{13}C NMR (DMSO-d_6, 100 MHz) spectra for **14a**. 18
Figure **S16** ^{1}H NMR (CD_3OD, 400 MHz) and ^{13}C NMR (CD_3OD, 100 MHz) spectra for **14b**. 19
Figure **S17** ^{1}H NMR (CD_3OD, 400 MHz) and ^{13}C NMR (CD_3OD, 100 MHz) spectra for **14c**. 20
Figure **S18** ^{1}H NMR (CD_3OD, 400 MHz) and ^{13}C NMR (CD_3OD, 100 MHz) spectra for **14d**. 21
Figure **S19** ^{1}H NMR (DMSO-d_6, 400 MHz) and ^{13}C NMR (DMSO-d_6, 100 MHz) spectra for **14e**. 22
Figure **S20** ^{1}H NMR (CD_3OD, 400 MHz) and ^{13}C NMR (CD_3OD, 100 MHz) spectra for **14f**. 23
Figure **S21** ^{1}H NMR (DMSO-d_6, 400 MHz) and ^{13}C NMR (DMSO-d_6, 100 MHz) spectra for **15a**. 24
Figure **S22** ^{1}H NMR (DMSO-d_6, 400 MHz) and ^{13}C NMR (DMSO-d_6, 100 MHz) spectra for **15b**. 25
Figure **S23** ^{1}H NMR (DMSO-d_6, 400 MHz) and ^{13}C NMR (DMSO-d_6, 100 MHz) spectra for **15c**. 26
Figure **S24** ^{1}H NMR (DMSO-d_6, 400 MHz) and ^{13}C NMR (DMSO-d_6, 100 MHz) spectra for **15d**. 27
Figure **S25** ^{1}H NMR (DMSO-d_6, 400 MHz) and ^{13}C NMR (DMSO-d_6, 100 MHz) spectra for **15e**. 28
Figure **S26** ^{1}H NMR (DMSO-d_6, 400 MHz and ^{13}C NMR (DMSO-d_6, 100 MHz) spectra for **15f**. 29
Figure **S27** ^{1}H NMR (DMSO-d_6, 400 MHz) and ^{13}C NMR (DMSO-d_6, 100 MHz) spectra for **16a**. 30
Figure **S28** ^{1}H NMR (DMSO-d_6, 400 MHz) and ^{13}C NMR (DMSO-d_6, 100 MHz) spectra for **16b**. 31
Figure **S29** ^{1}H NMR (DMSO-d_6, 400 MHz) and ^{13}C NMR (DMSO-d_6, 100 MHz) spectra for **16c**. 32
Figure **S30** ^{1}H NMR (DMSO-d_6, 400 MHz) and ^{13}C NMR (DMSO-d_6, 100 MHz) spectra for **16d**. 33
Figure **S31** ^{1}H NMR (DMSO-d_6, 400 MHz) and ^{13}C NMR (DMSO-d_6, 100 MHz) spectra for **16e**. 34
Figure **S32** ^{1}H NMR (DMSO-d_6, 400 MHz) and ^{13}C NMR (DMSO-d_6, 100 MHz) spectra for **16f**. 35
Figure **S33** ^{1}H NMR (DMSO-d_6, 400 MHz) and ^{13}C NMR (DMSO-d_6, 100 MHz) spectra for **17a**. 36
Figure **S34** ^{1}H NMR (DMSO-d_6, 400 MHz) and ^{13}C NMR (DMSO-d_6, 100 MHz) spectra for **17b**. 37
Figure **S35** ^{1}H NMR (DMSO-d_6, 400 MHz) and ^{13}C NMR (DMSO-d_6, 100 MHz) spectra for **17c**. 38
Figure **S36** ^{1}H NMR (DMSO-d_6, 400 MHz) and ^{13}C NMR (DMSO-d_6, 100 MHz) spectra for **17d**. 39
Figure **S37** ^{1}H NMR (DMSO-d_6, 400 MHz) and ^{13}C NMR (DMSO-d_6, 100 MHz) spectra for **17e**. 40
Figure **S38** ^{1}H NMR (DMSO-d_6, 400 MHz) and ^{13}C NMR (DMSO-d_6, 100 MHz) spectra for **17f**. 41
Figure **S39** ^{1}H NMR (DMSO-d_6, 400 MHz) and ^{13}C NMR (DMSO-d_6, 100 MHz) spectra for **18a**. 42
Figure **S40** ^{1}H NMR (DMSO-d_6, 400 MHz) and ^{13}C NMR (DMSO-d_6, 100 MHz) spectra for **18b**. 43

Figure **S41** ^{1}H NMR (DMSO-d_6, 400 MHz) and ^{13}C NMR (DMSO-d_6, 100 MHz) spectra for **18c**. 44
Figure **S42** ^{1}H NMR (DMSO-d_6, 400 MHz) and ^{13}C NMR (DMSO-d_6, 100 MHz) spectra for **18d**..................... 45
Figure **S43** ^{1}H NMR (DMSO-d_6, 400 MHz) and ^{13}C NMR (DMSO-d_6, 100 MHz) spectra for **18e**. 46
Figure **S44** ^{1}H NMR (DMSO-d_6, 400 MHz) and ^{13}C NMR (DMSO-d_6, 100 MHz) spectra for **18f**...................... 47

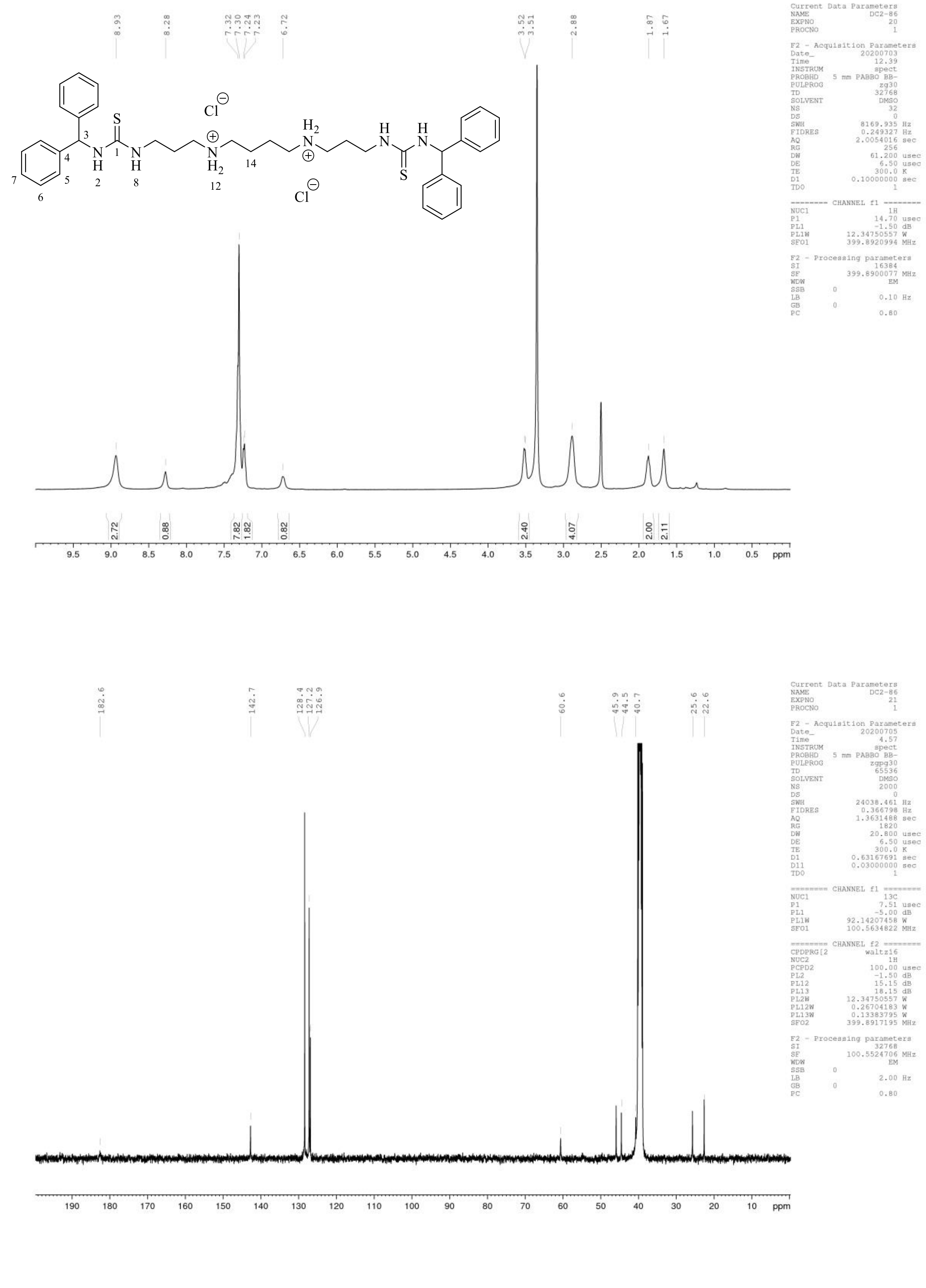

Figure **S1** ^{1}H NMR (DMSO-d_6, 400 MHz) and ^{13}C NMR (DMSO-d_6, 100 MHz) spectra for **6a**.

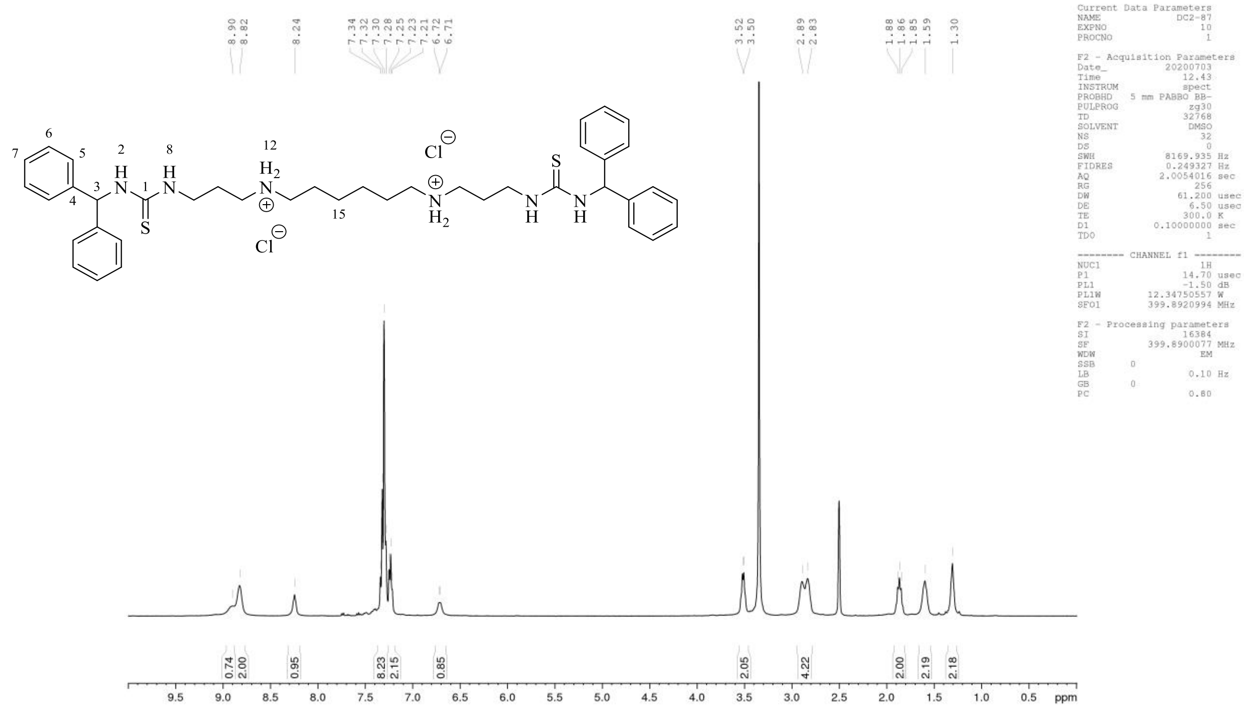

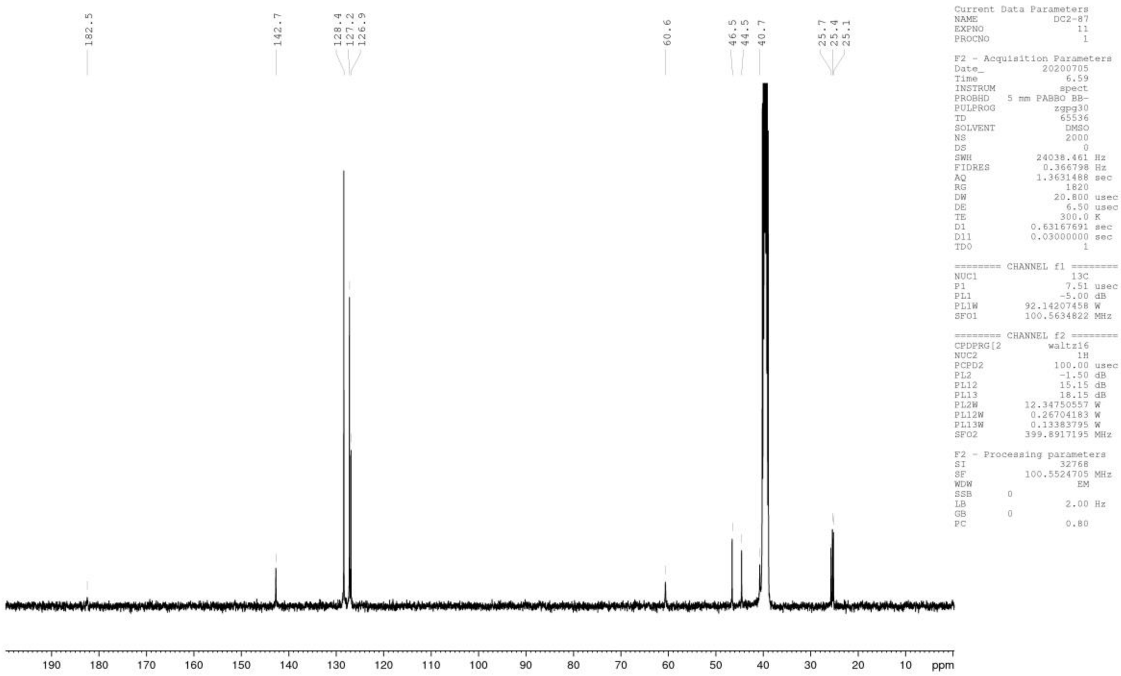

Figure **S2** ^{1}H NMR (DMSO-d_6, 400 MHz) and ^{13}C NMR (DMSO-d_6, 100 MHz) spectra for **6b**.

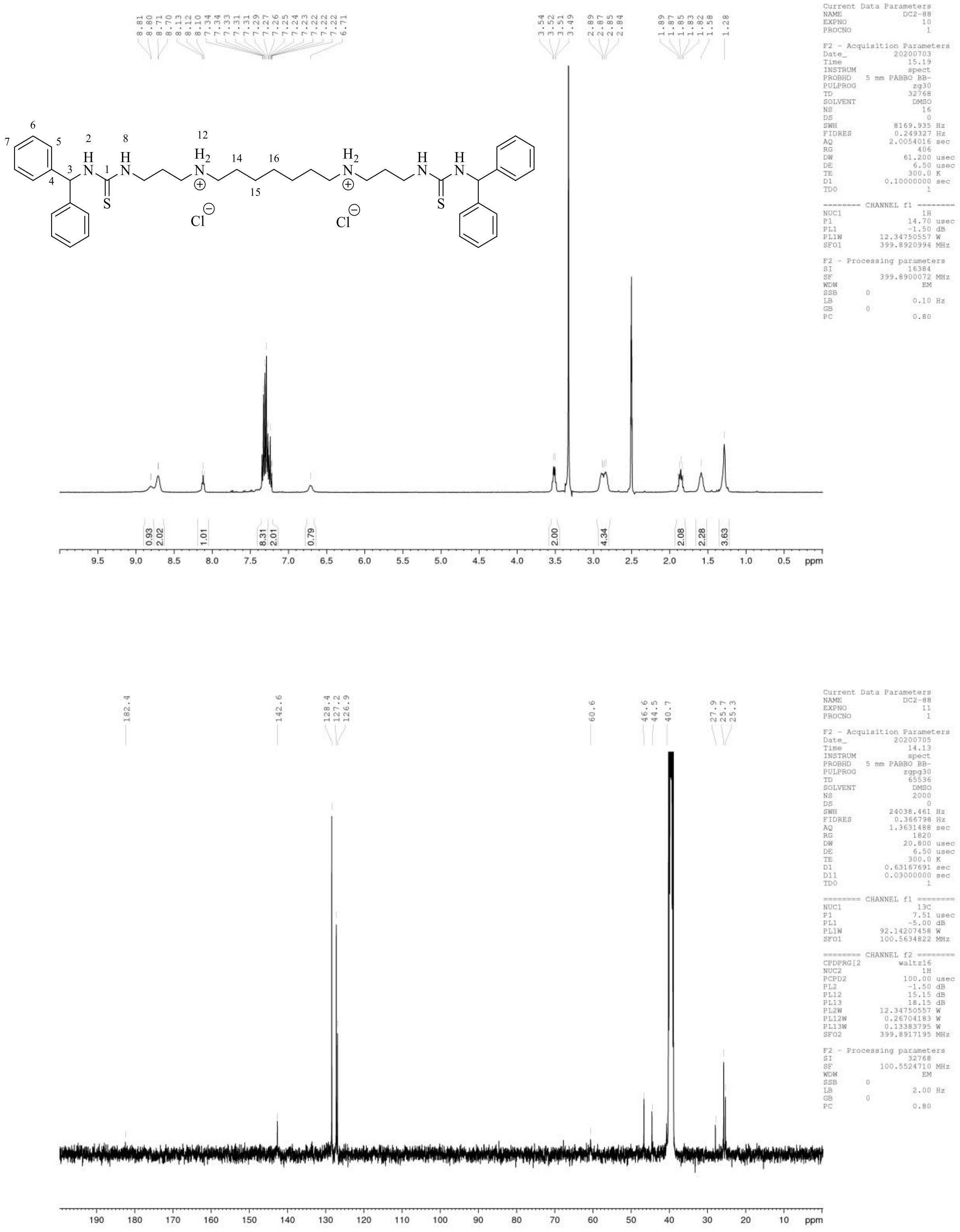

Figure **S3** ^{1}H NMR (DMSO-d_6, 400 MHz) and ^{13}C NMR (DMSO-d_6, 100 MHz) spectra for **6c**.

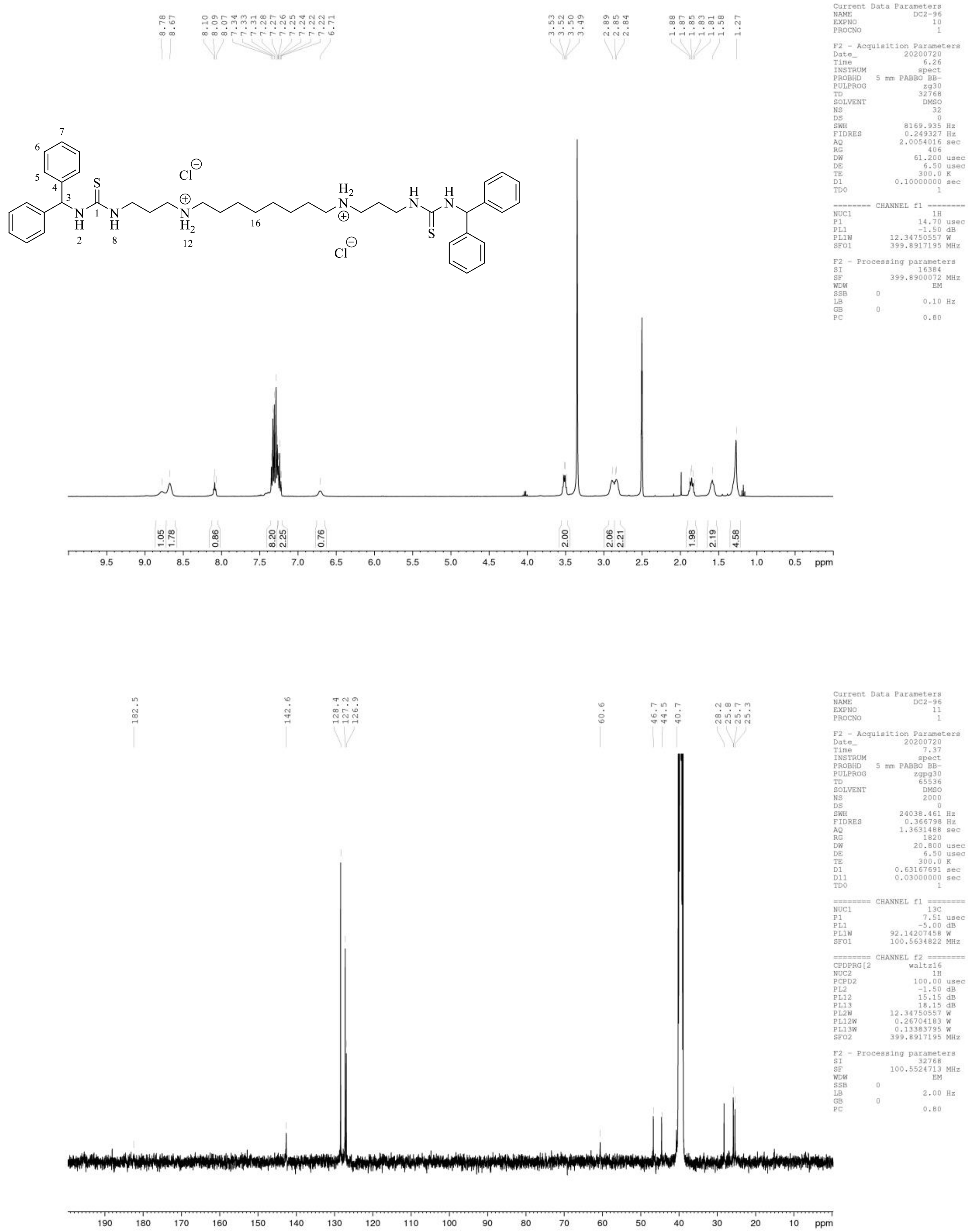

Figure **S4** ^{1}H NMR (DMSO-d_6, 400 MHz) and ^{13}C NMR (DMSO-d_6, 100 MHz) spectra for **6d**.

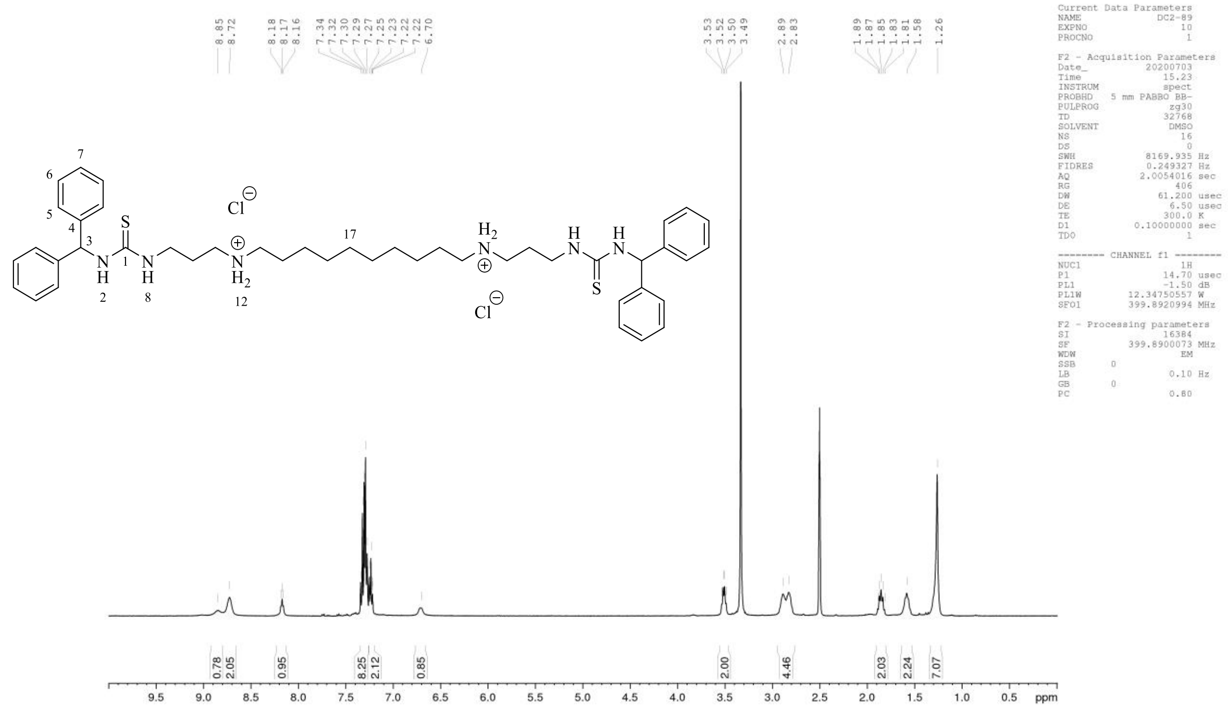

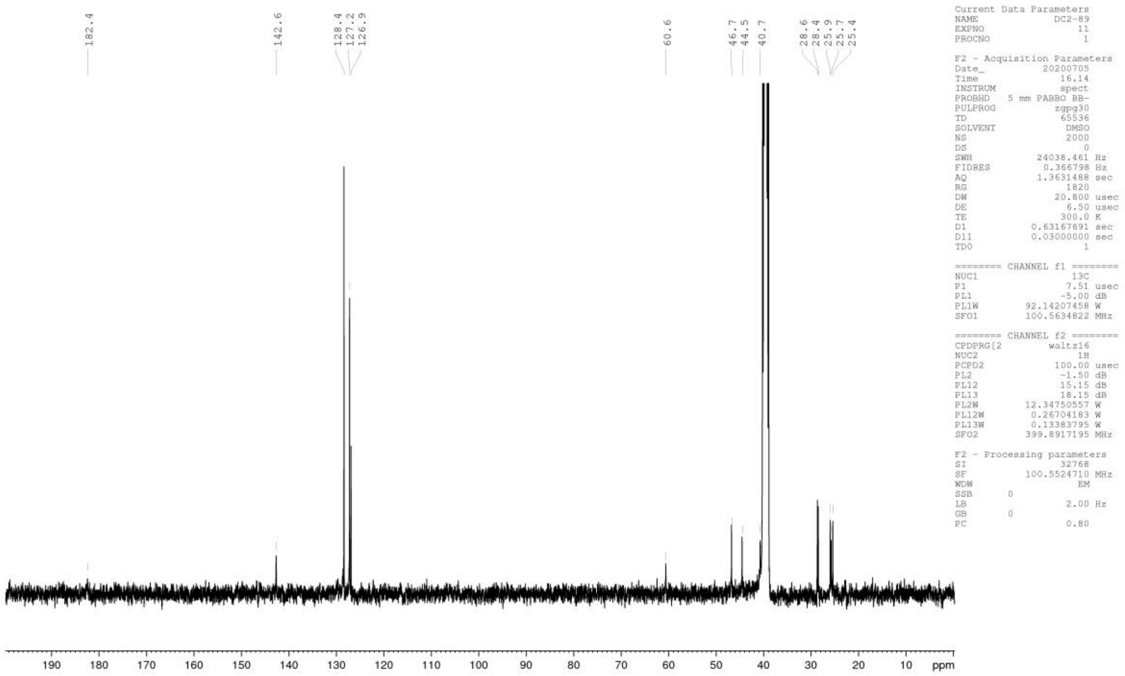

Figure **S5** ^{1}H NMR (DMSO-d_6, 400 MHz) and ^{13}C NMR (DMSO-d_6, 100 MHz) spectra for **6e**.

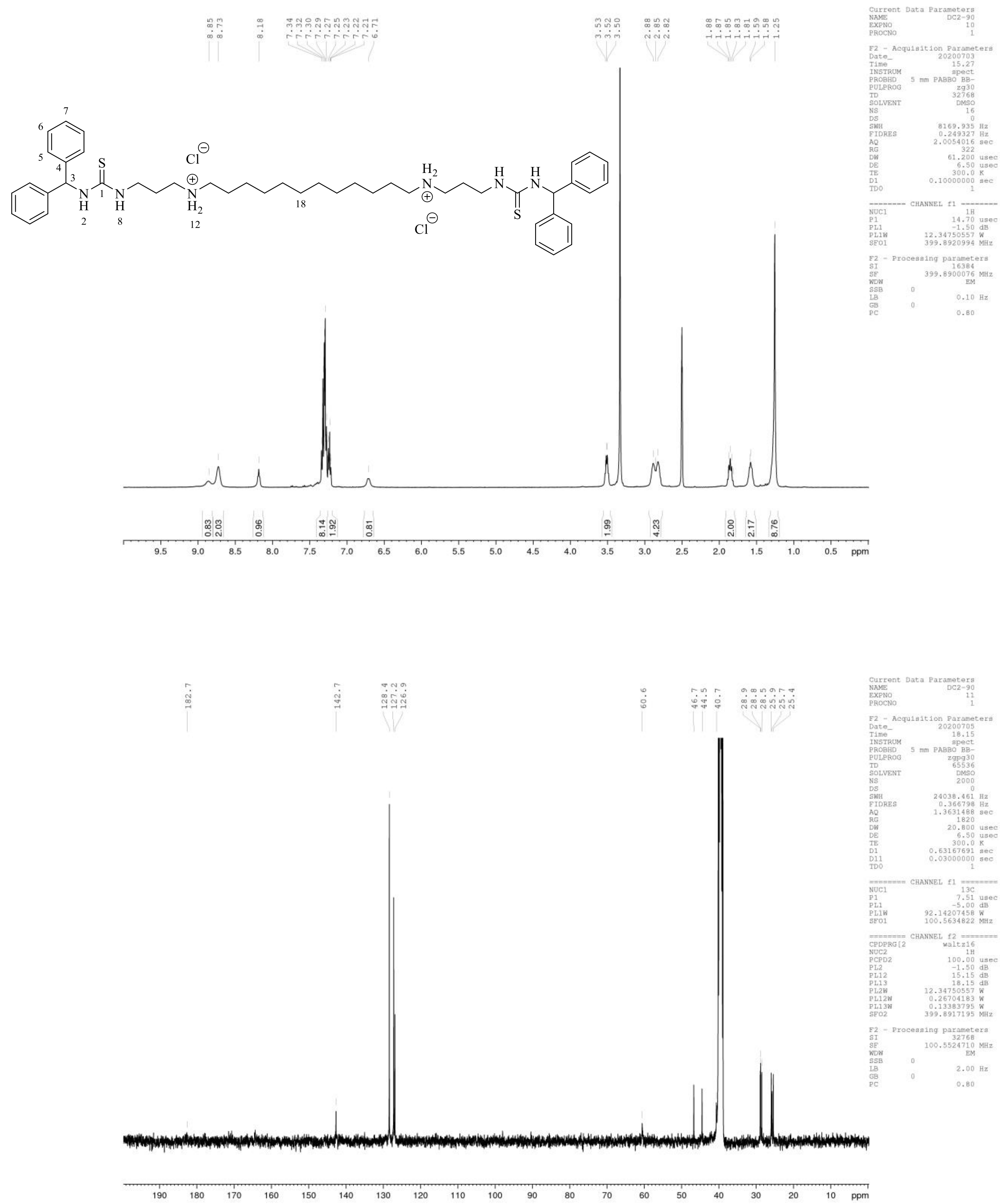

Figure **S6** ^{1}H NMR (DMSO-d_6, 400 MHz) and ^{13}C NMR (DMSO-d_6, 100 MHz) spectra for **6f**.

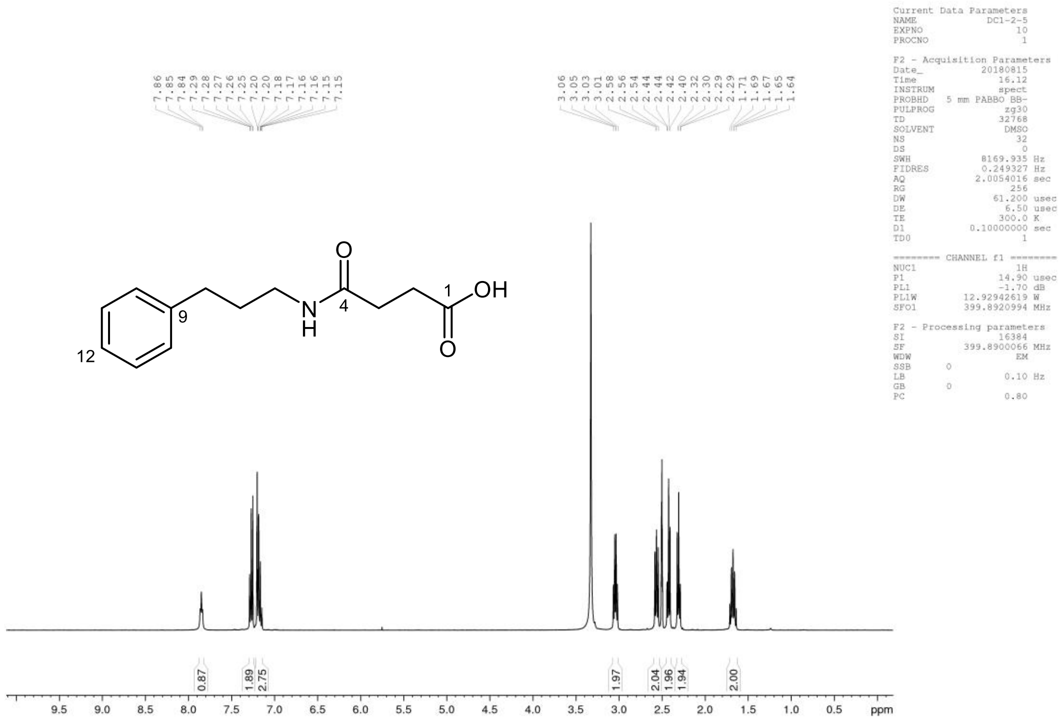

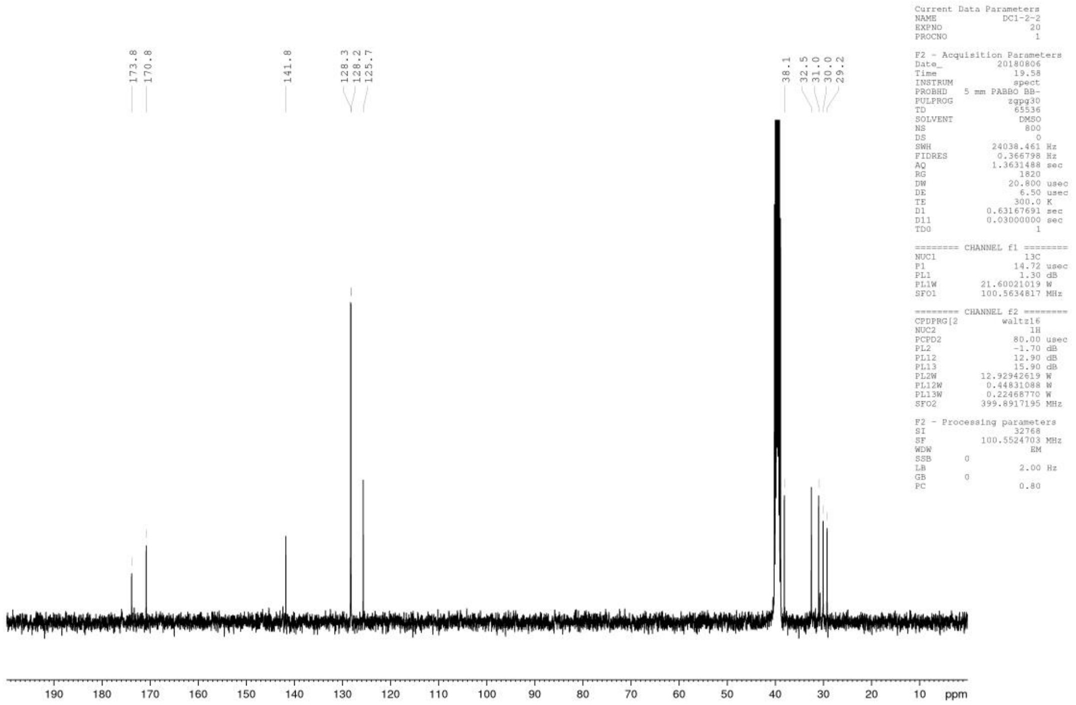

Figure **S7** ^{1}H NMR (DMSO-d_6, 400 MHz) and ^{13}C NMR (DMSO-d_6, 100 MHz) spectra for **8**.

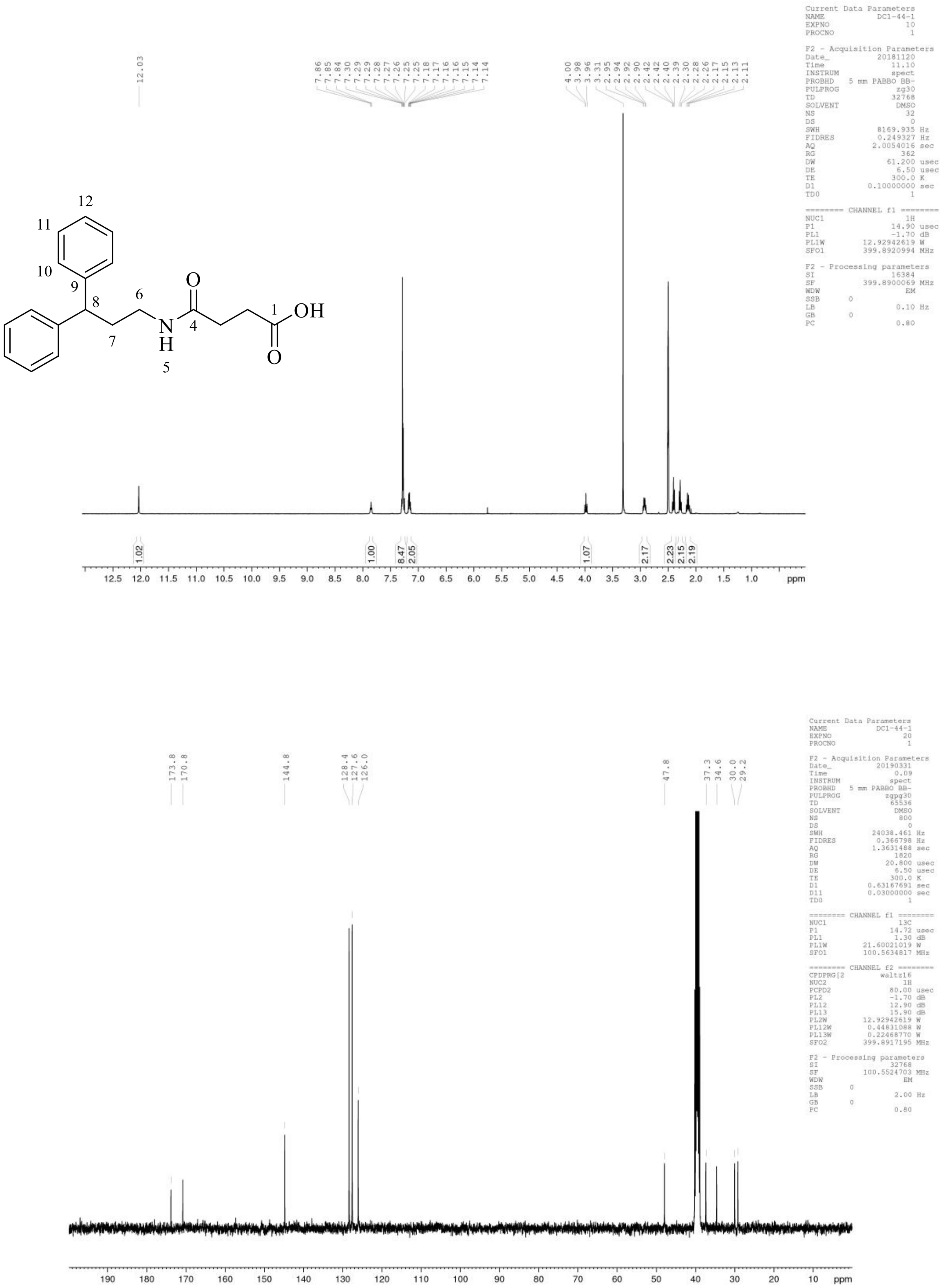

Figure **S8** ^{1}H NMR (DMSO-d_6, 400 MHz) and ^{13}C NMR (DMSO-d_6, 100 MHz) spectra for **10**.

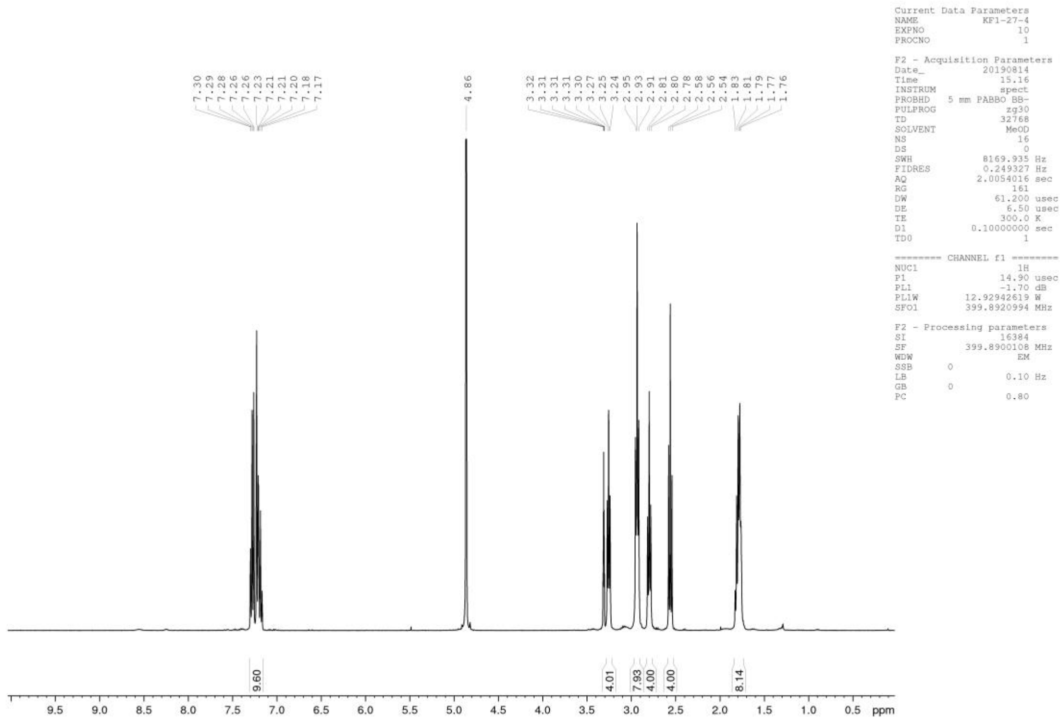

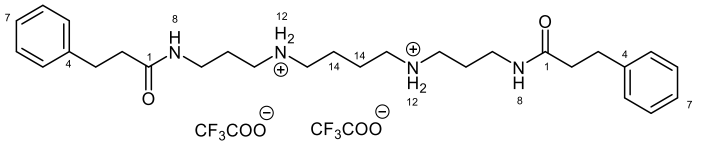

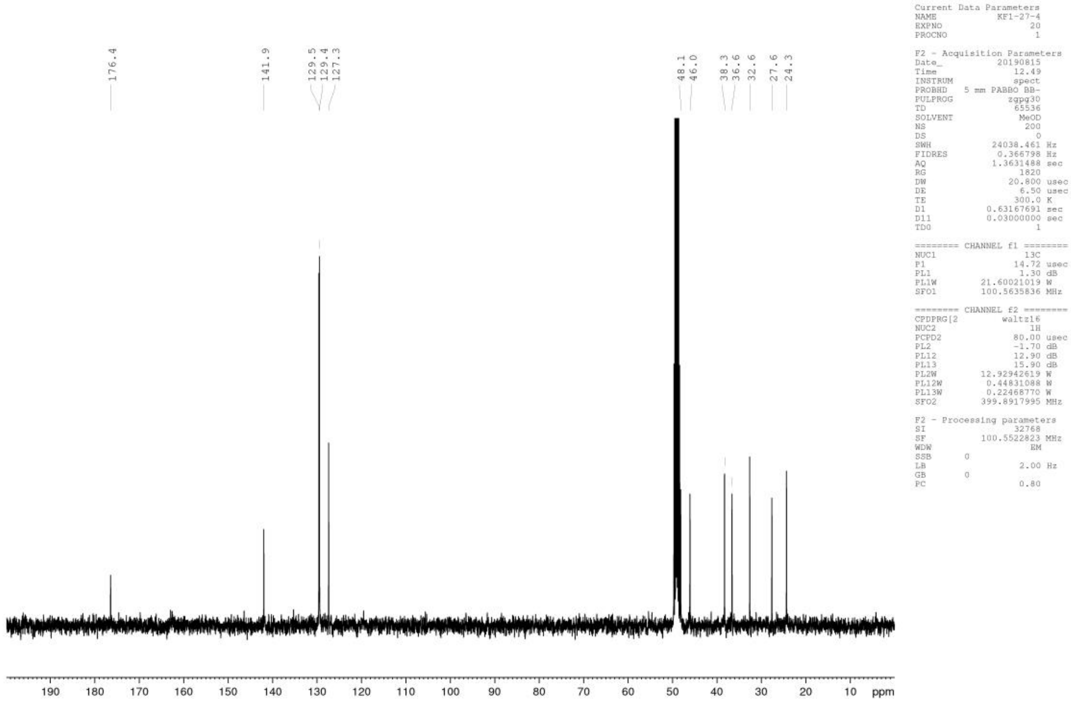

Figure **S9** ^{1}H NMR (CD_3OD, 400 MHz) and ^{13}C NMR (CD_3OD, 100 MHz) spectra for **13a**.

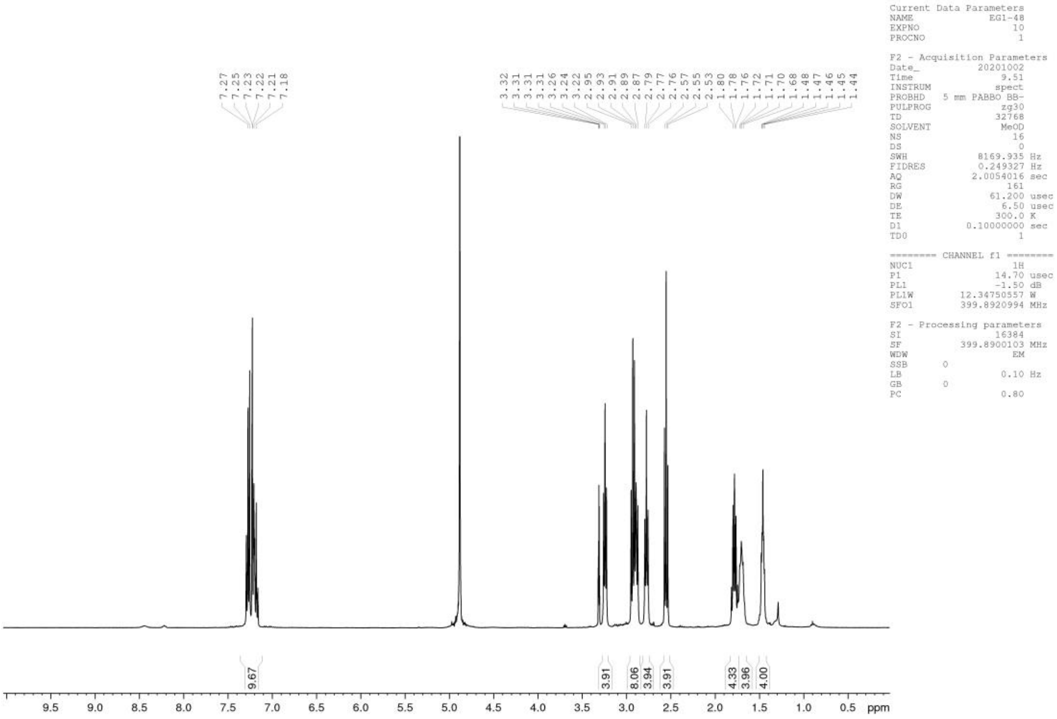

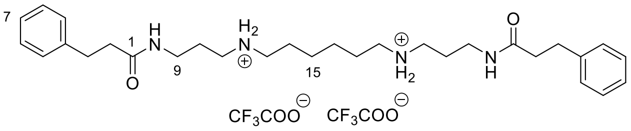

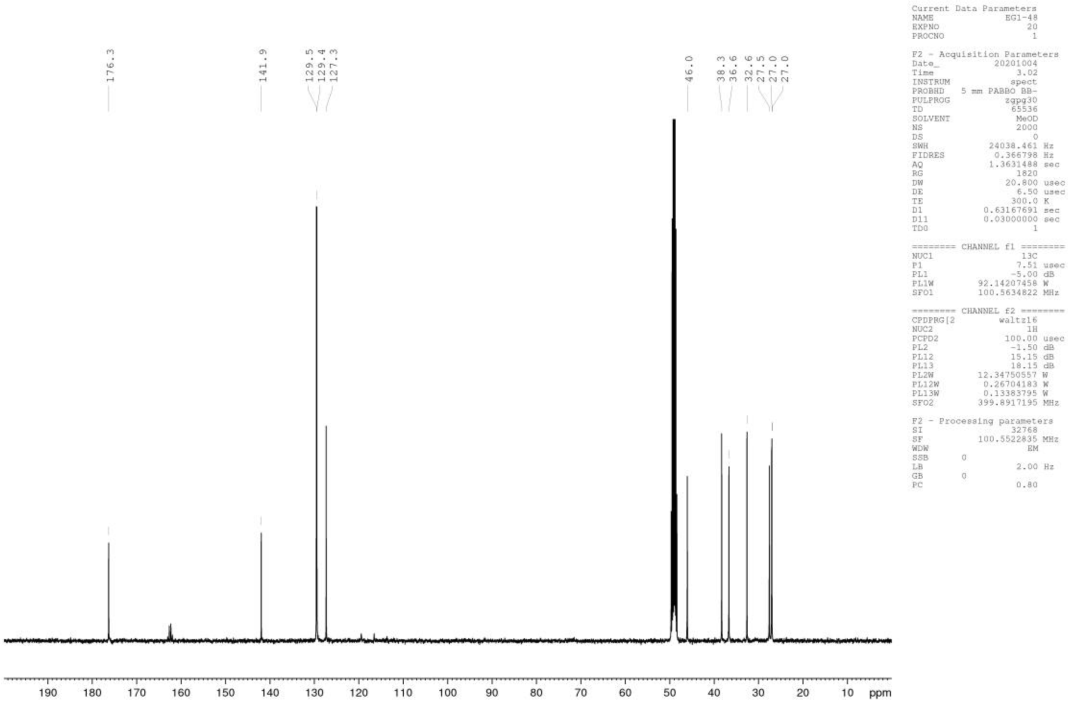

Figure **S10** ^{1}H NMR (CD_3OD, 400 MHz) and ^{13}C NMR (CD_3OD, 100 MHz) spectra for **13b**.

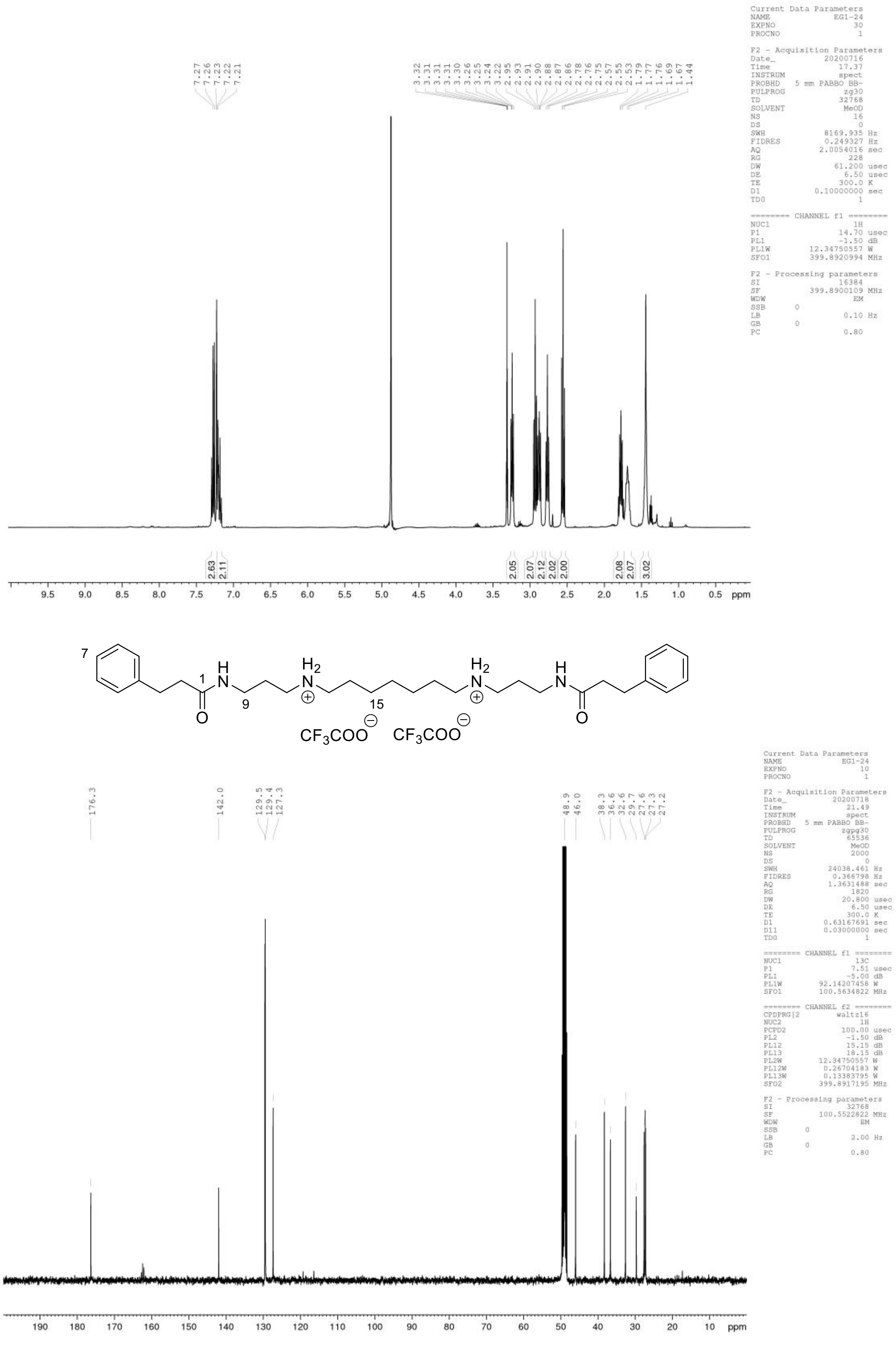

Figure **S11** ^{1}H NMR (CD_3OD, 400 MHz) and ^{13}C NMR (CD_3OD, 100 MHz) spectra for **13c**.

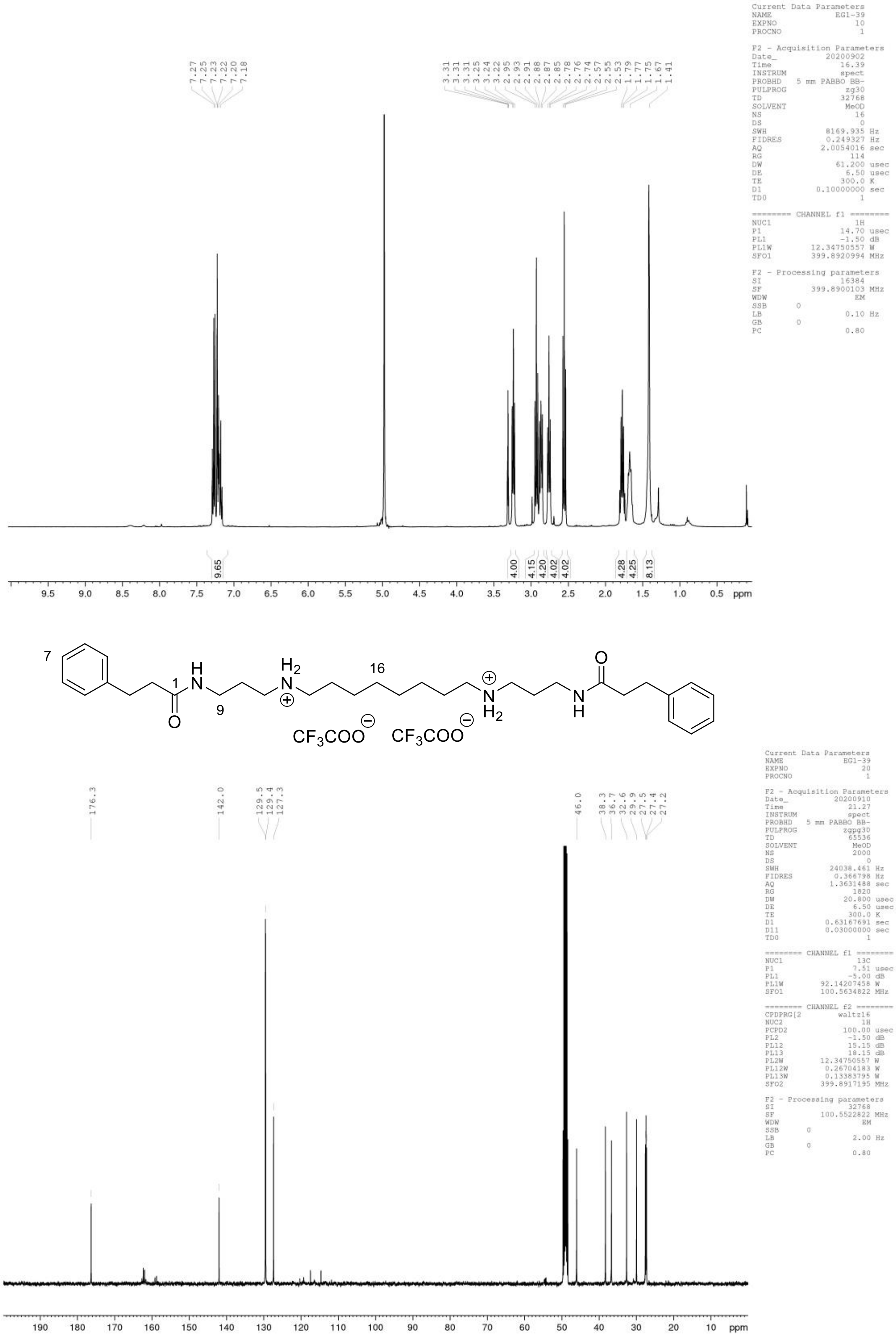

Figure **S12** ^{1}H NMR (CD_3OD, 400 MHz) and ^{13}C NMR (CD_3OD, 100 MHz) spectra for **13d**.

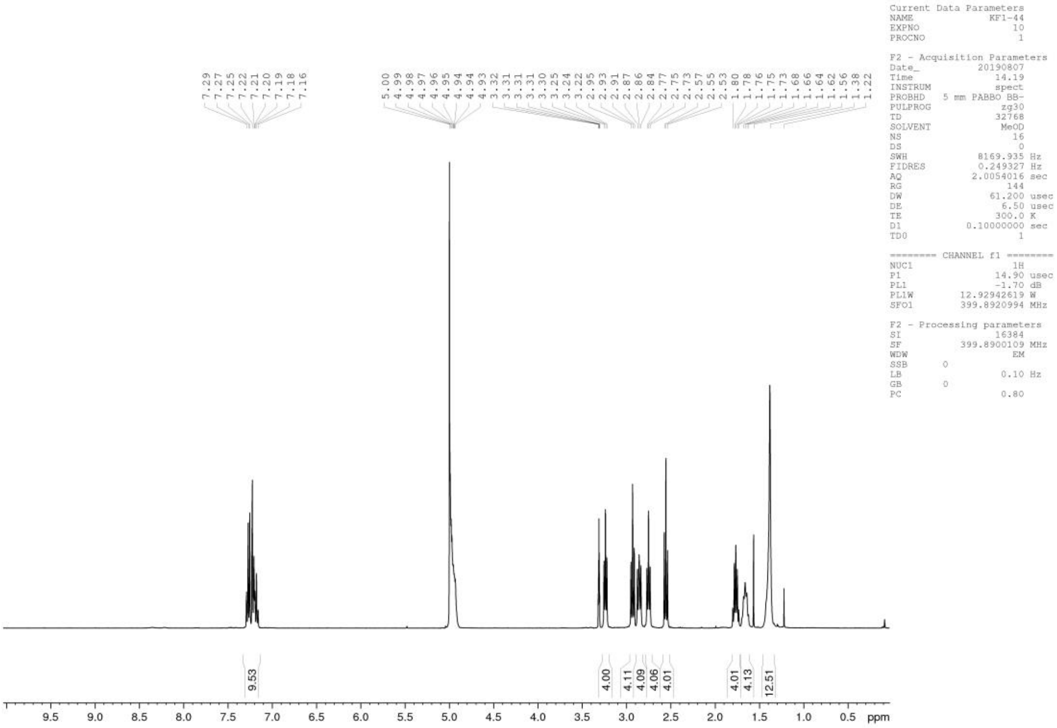

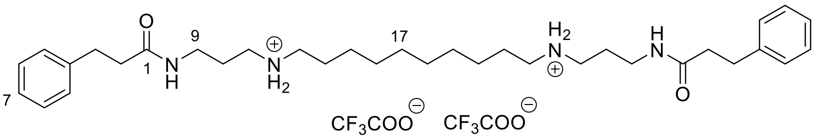

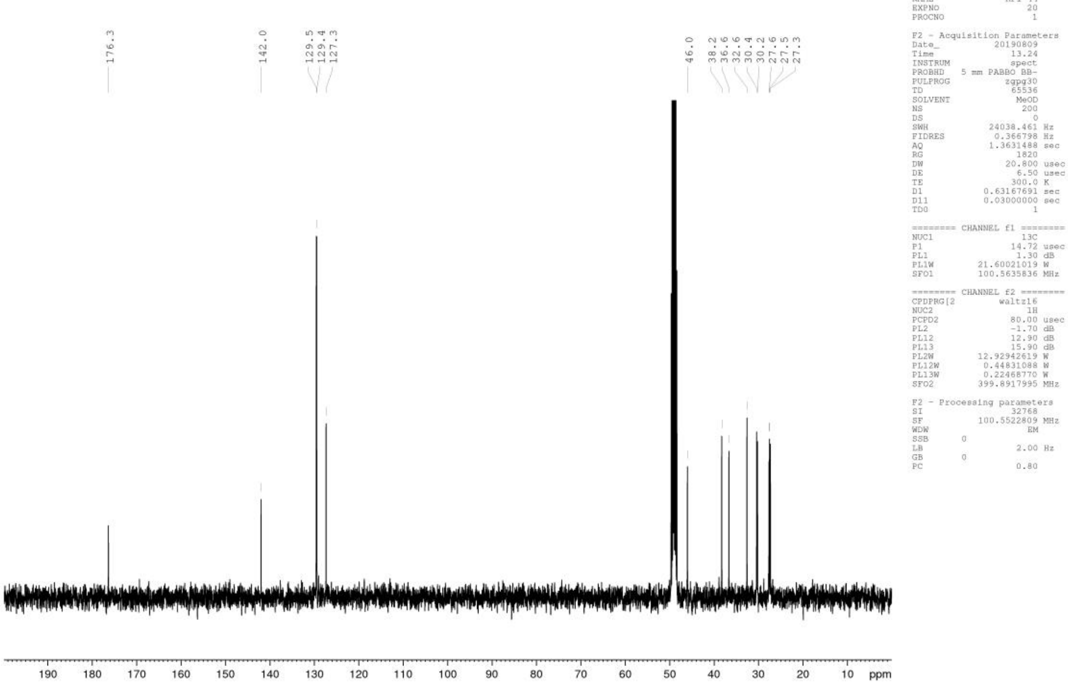

Figure **S13** ^{1}H NMR (CD_3OD, 400 MHz) and ^{13}C NMR (CD_3OD, 100 MHz) spectra for **13e**.

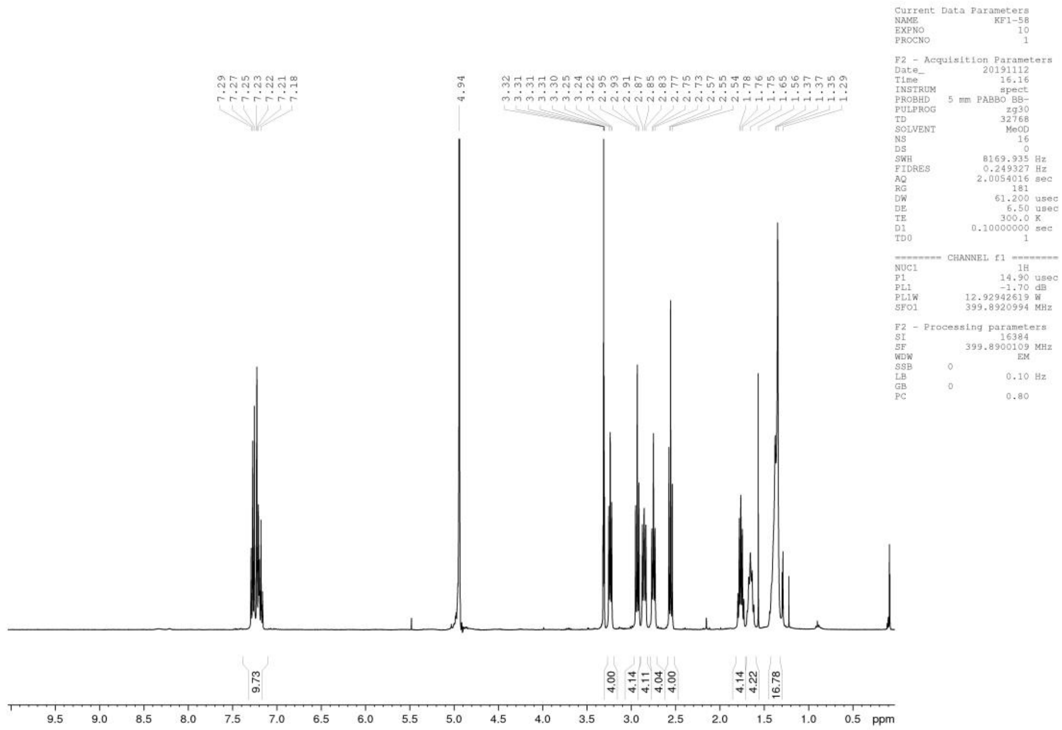

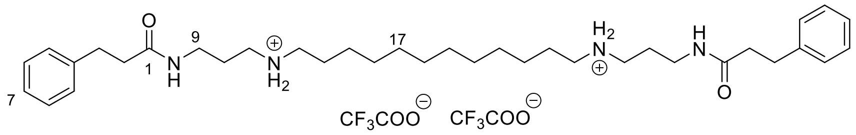

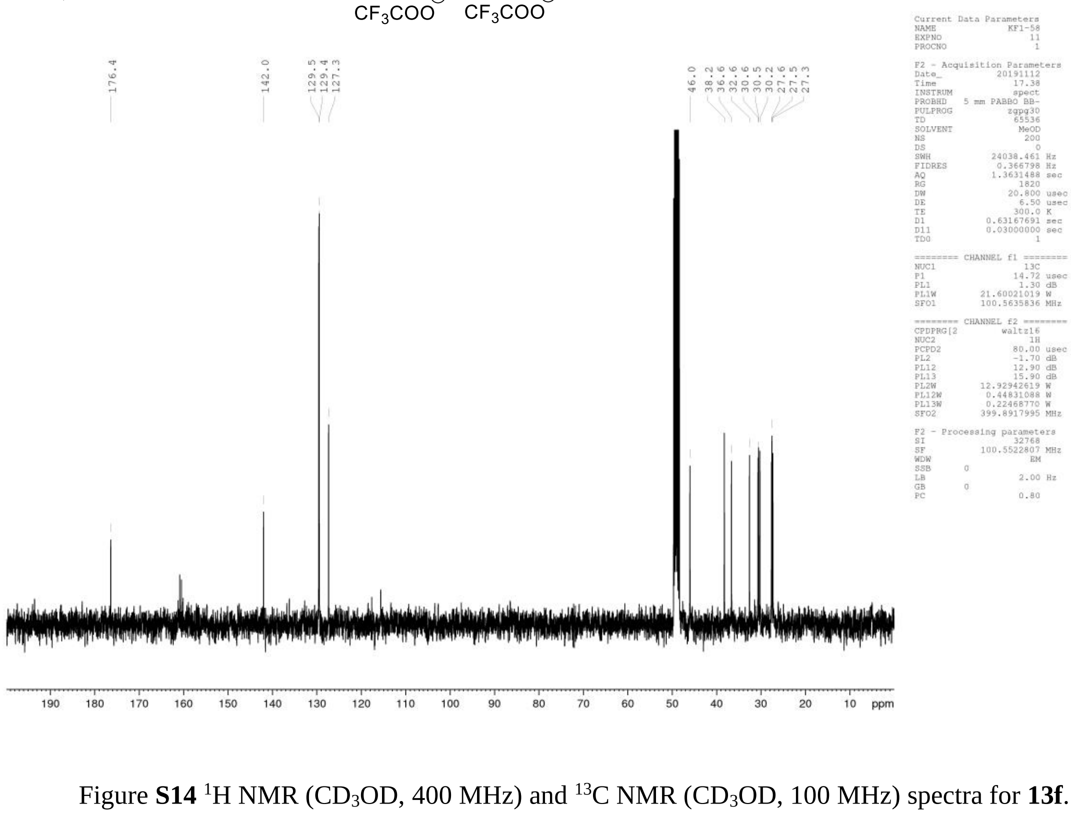

Figure **S14** ^{1}H NMR (CD_3OD, 400 MHz) and ^{13}C NMR (CD_3OD, 100 MHz) spectra for **13f**.

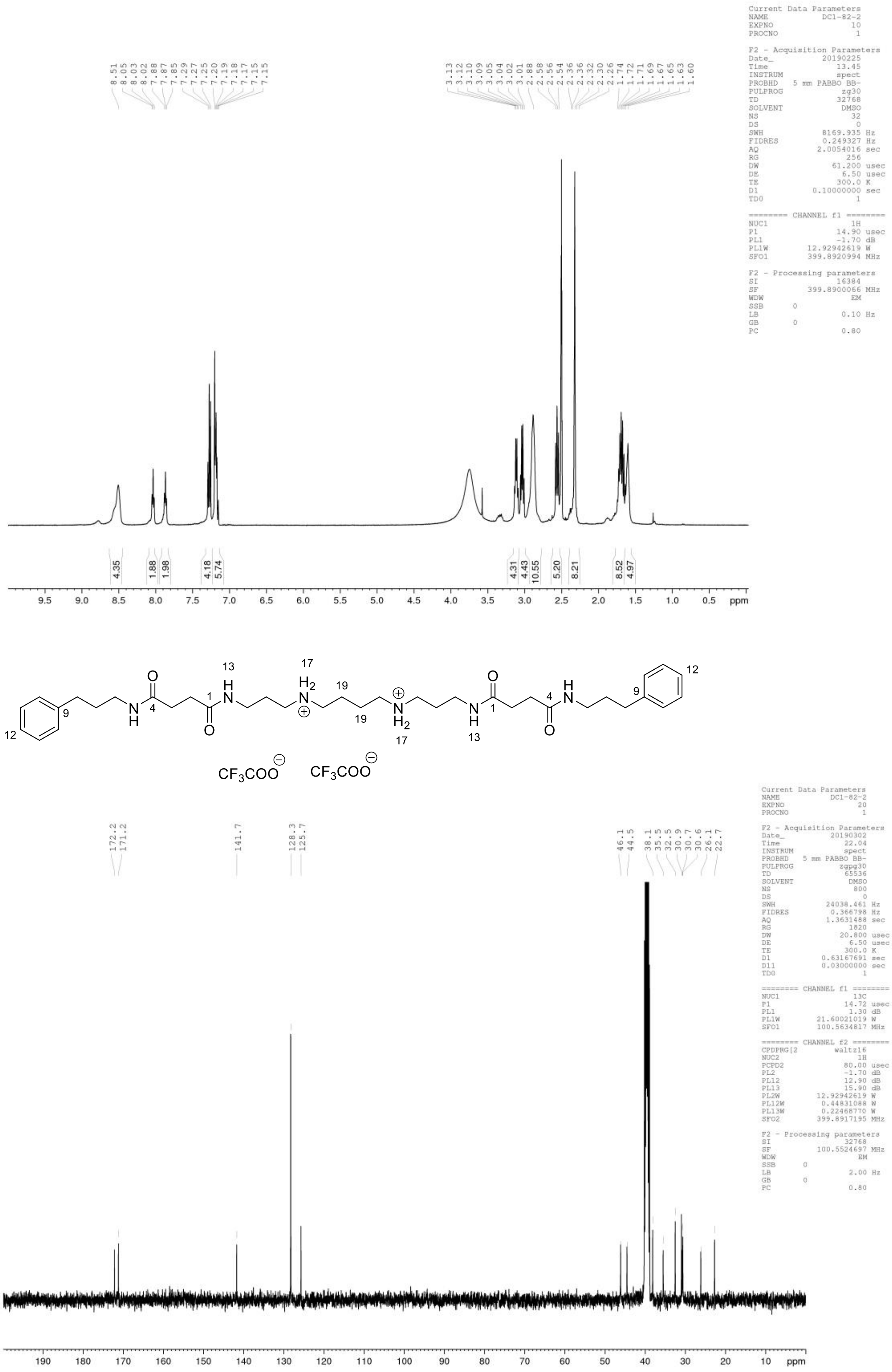

Figure **S15** ^{1}H NMR (DMSO-d_6, 400 MHz) and ^{13}C NMR (DMSO-d_6, 100 MHz) spectra for **14a**.

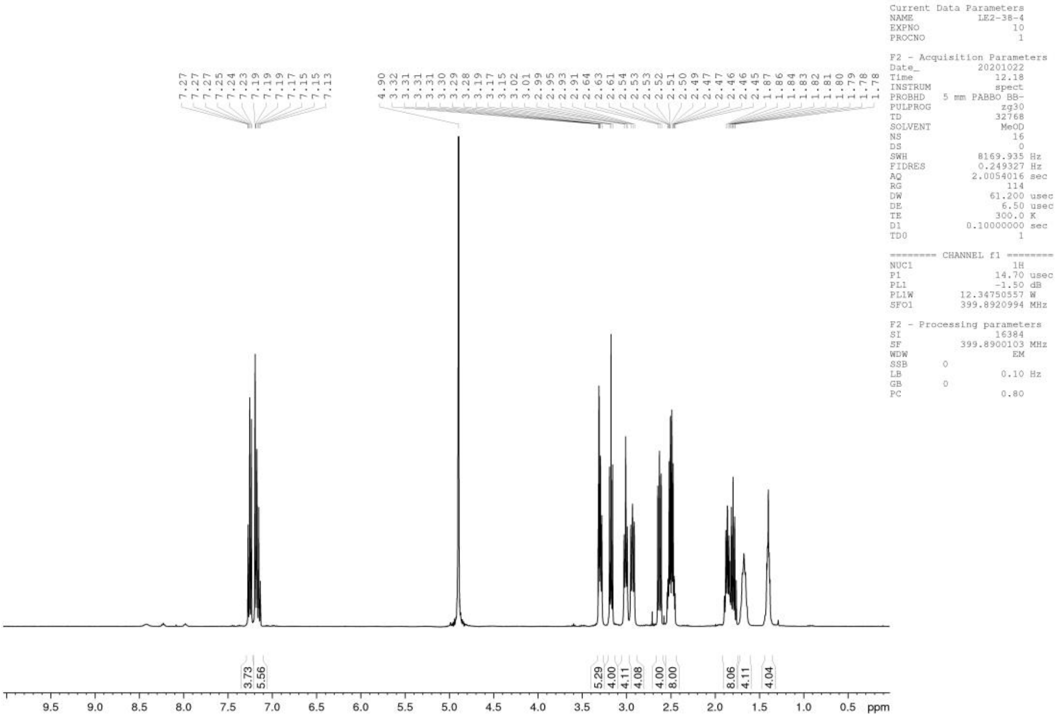

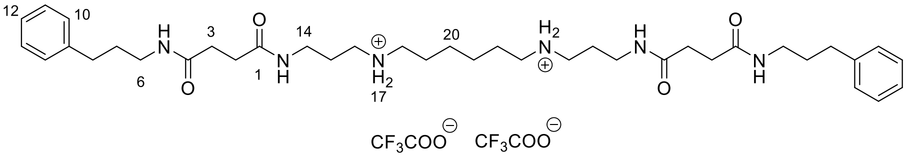

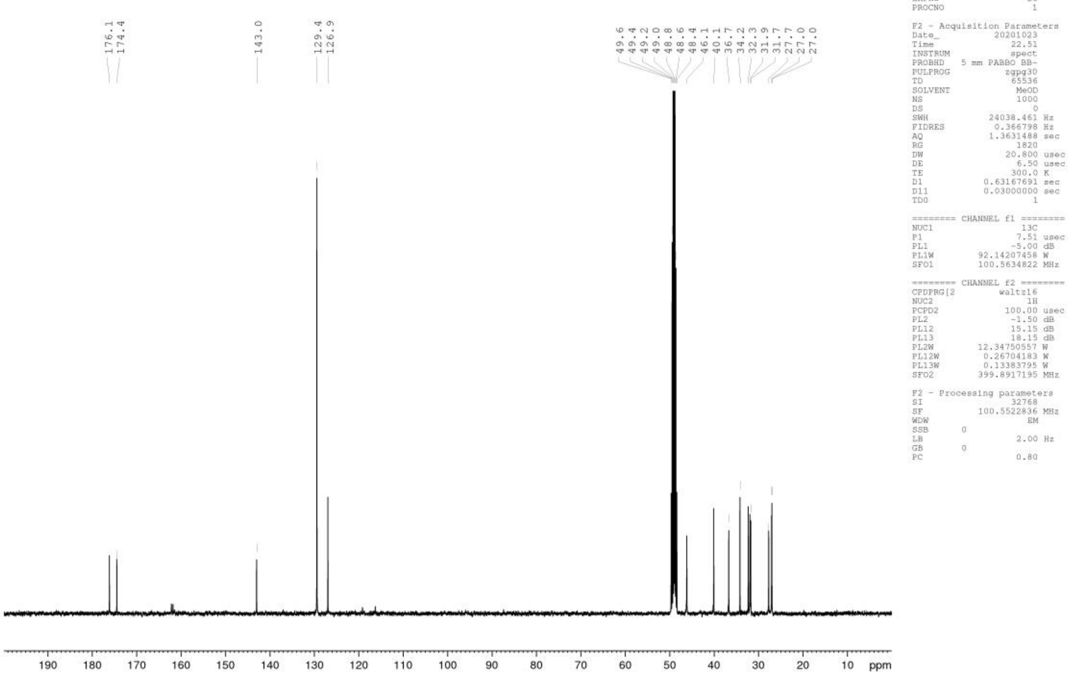

Figure **S16** ^{1}H NMR (CD_3OD, 400 MHz) and ^{13}C NMR (CD_3OD, 100 MHz) spectra for **14b**.

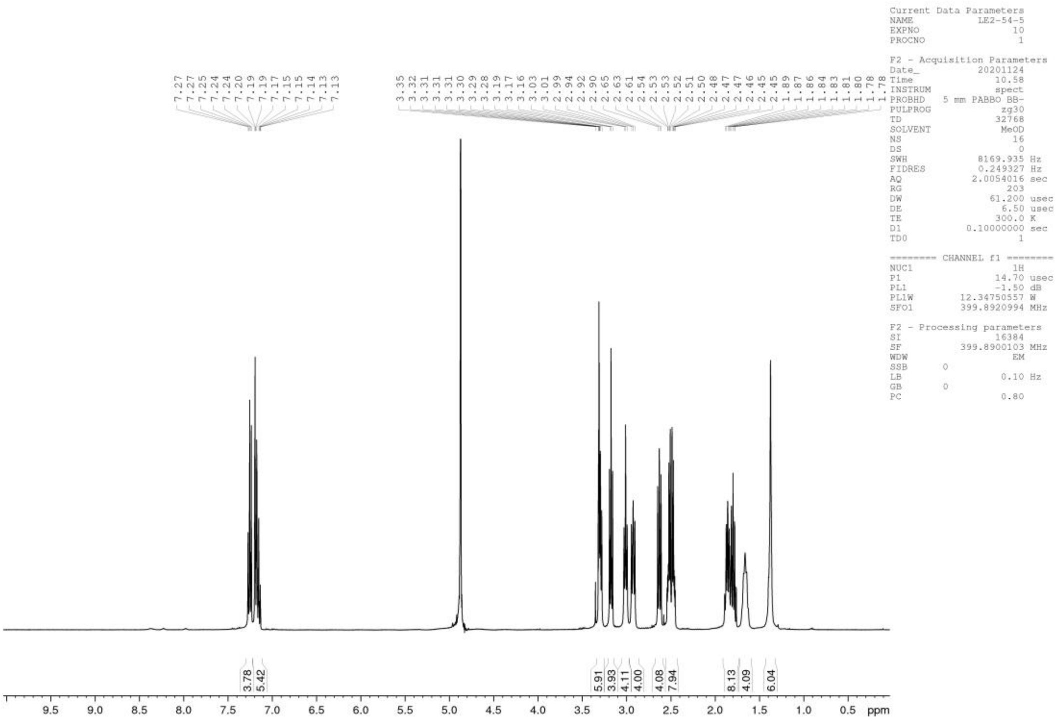

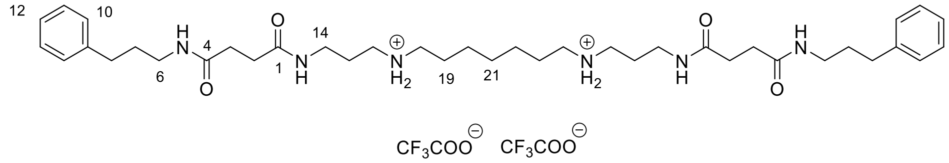

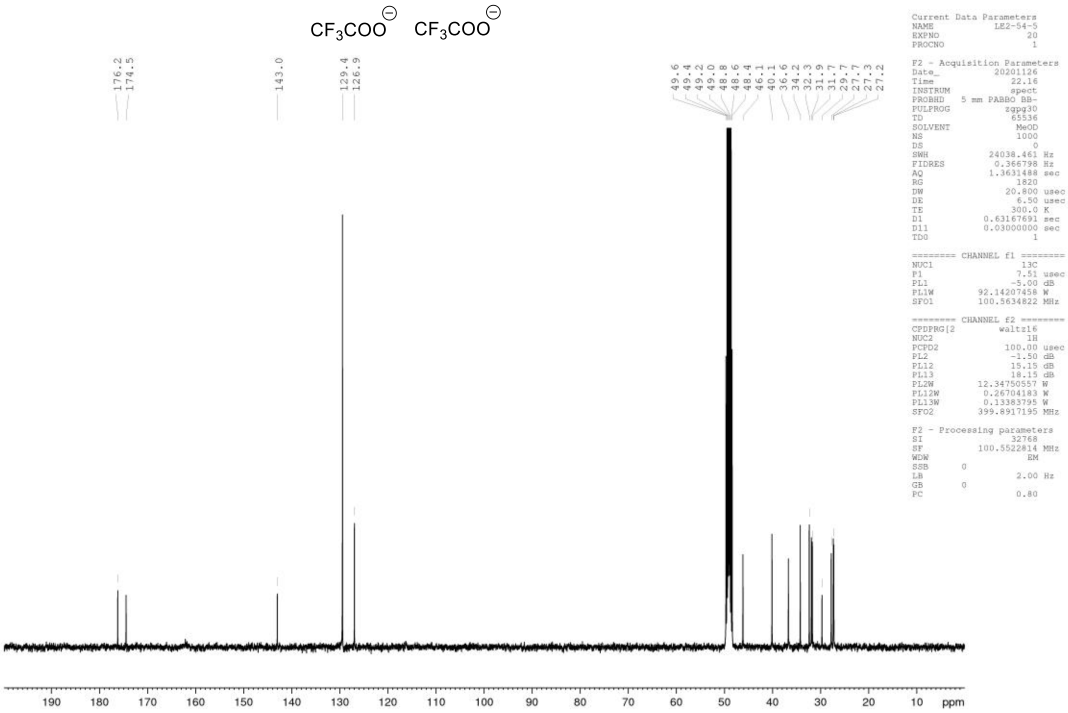

Figure **S17** ^{1}H NMR (CD_3OD, 400 MHz) and ^{13}C NMR (CD_3OD, 100 MHz) spectra for **14c**.

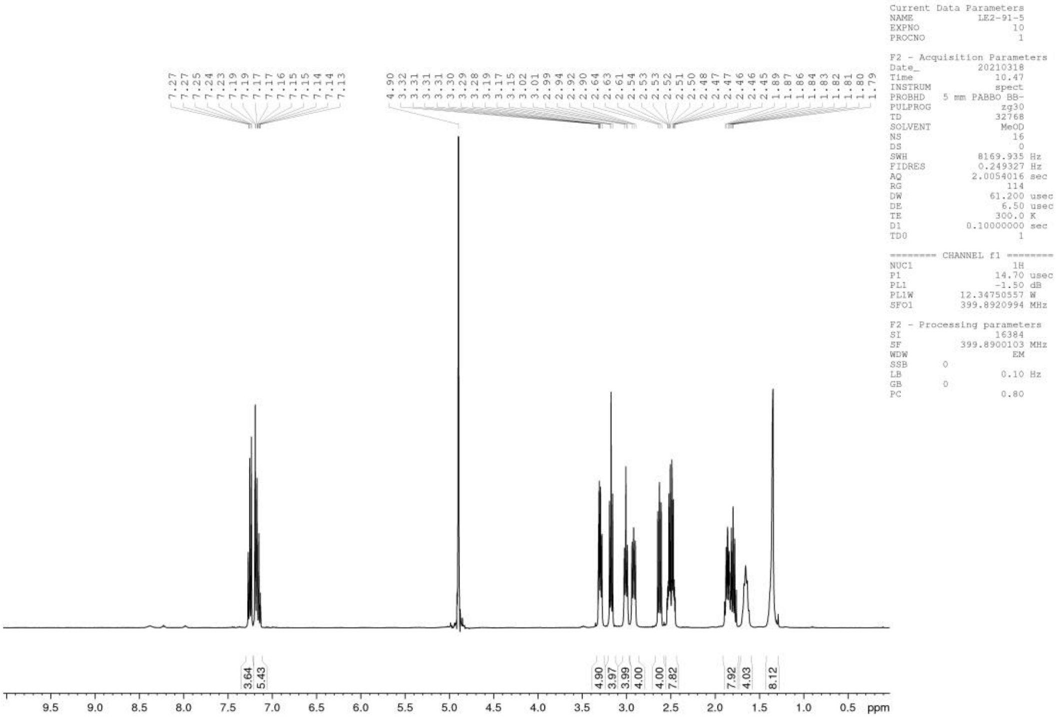

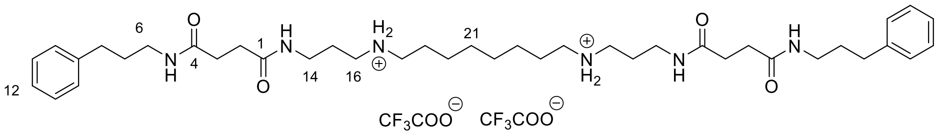

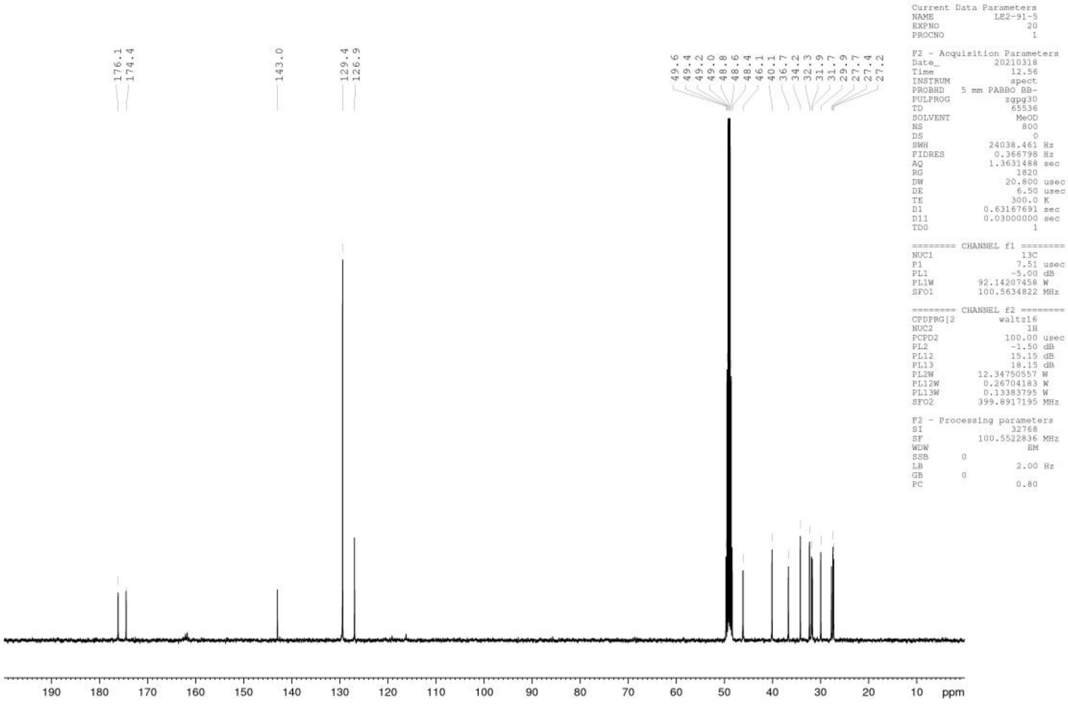

Figure **S18** ^{1}H NMR (CD_3OD, 400 MHz) and ^{13}C NMR (CD_3OD, 100 MHz) spectra for **14d**.

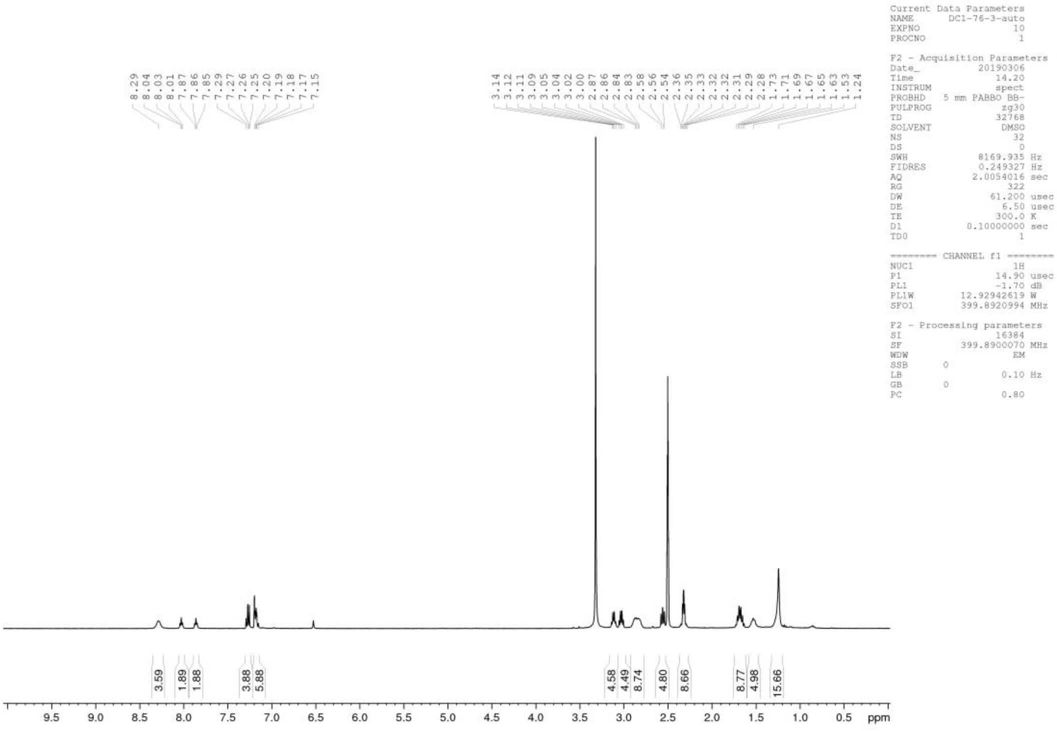

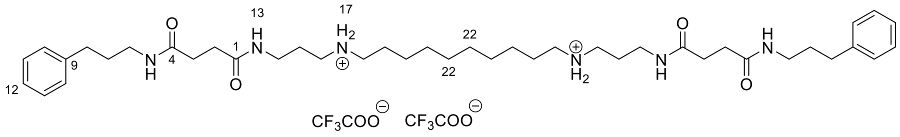

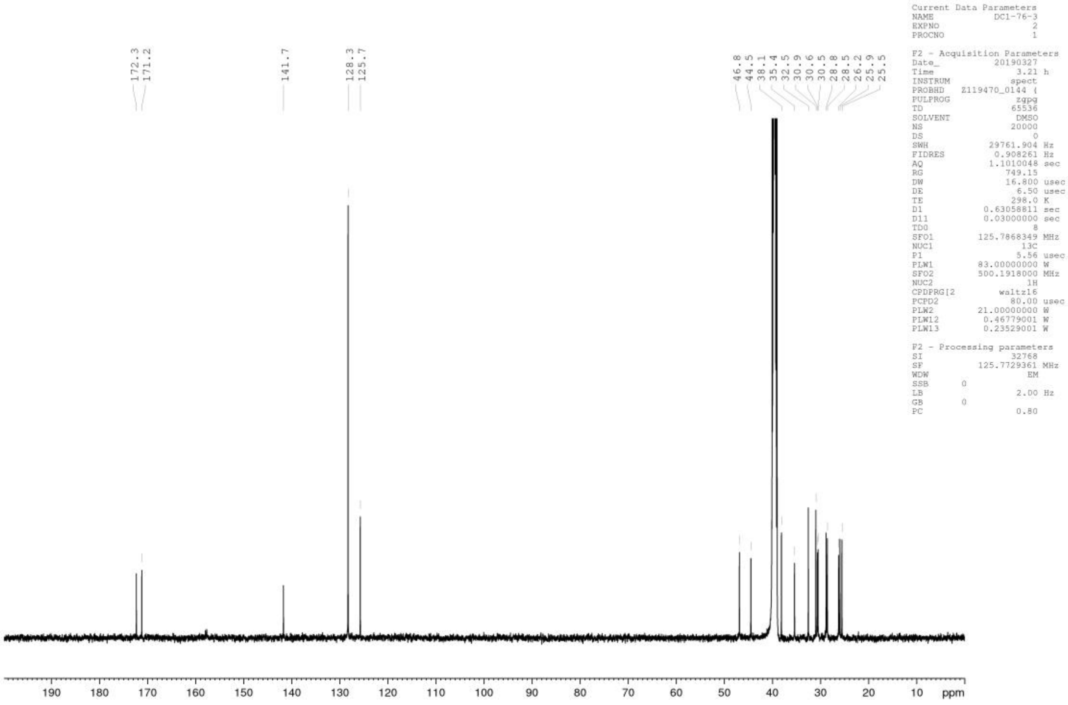

Figure **S19** ^{1}H NMR (DMSO-d_6, 400 MHz) and ^{13}C NMR (DMSO-d_6, 100 MHz) spectra for **14e**.

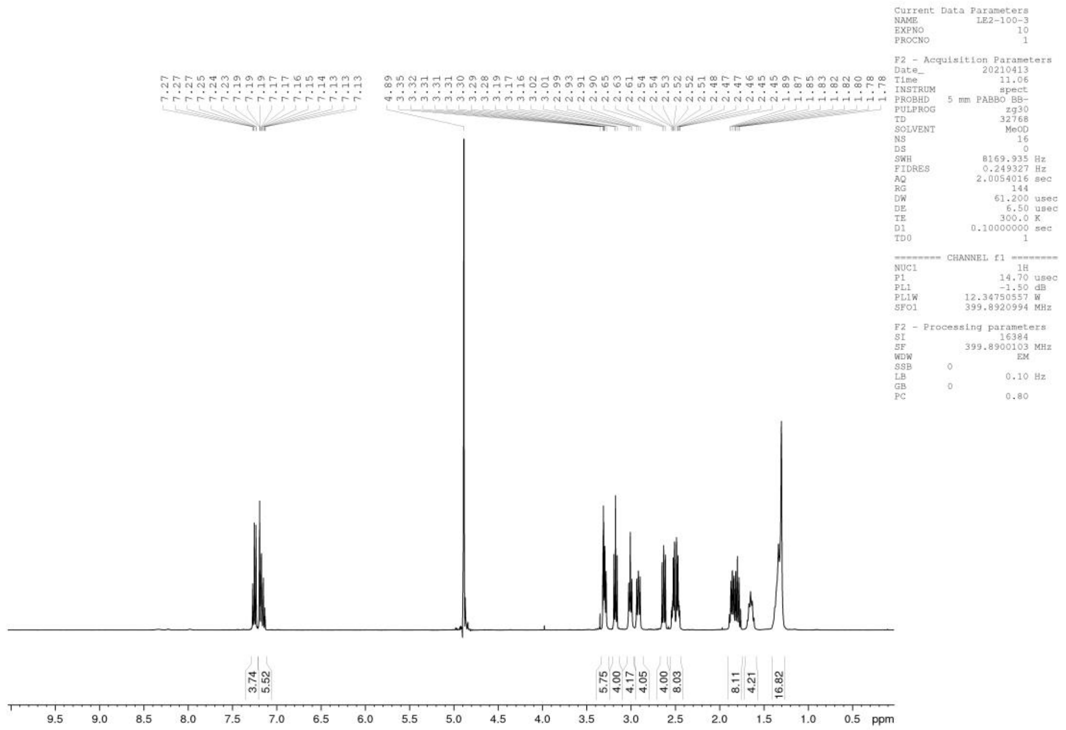

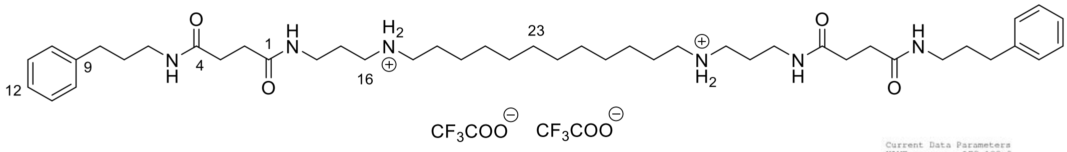

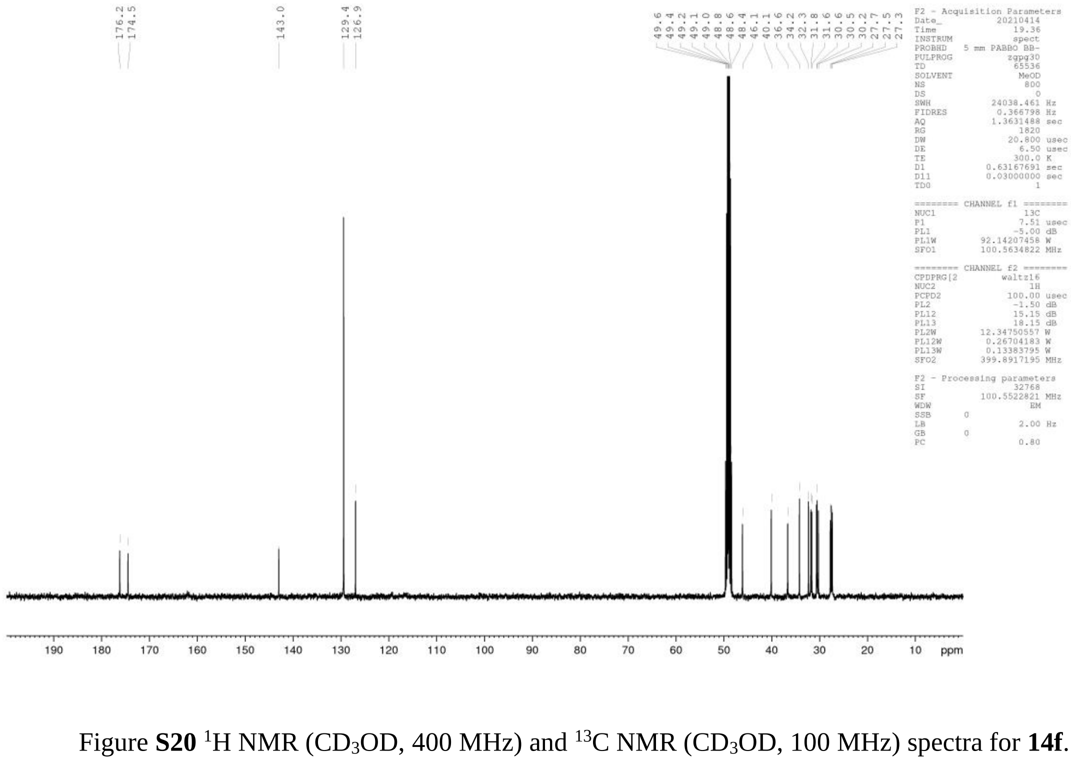

Figure **S20** ^{1}H NMR (CD_3OD, 400 MHz) and ^{13}C NMR (CD_3OD, 100 MHz) spectra for **14f**.

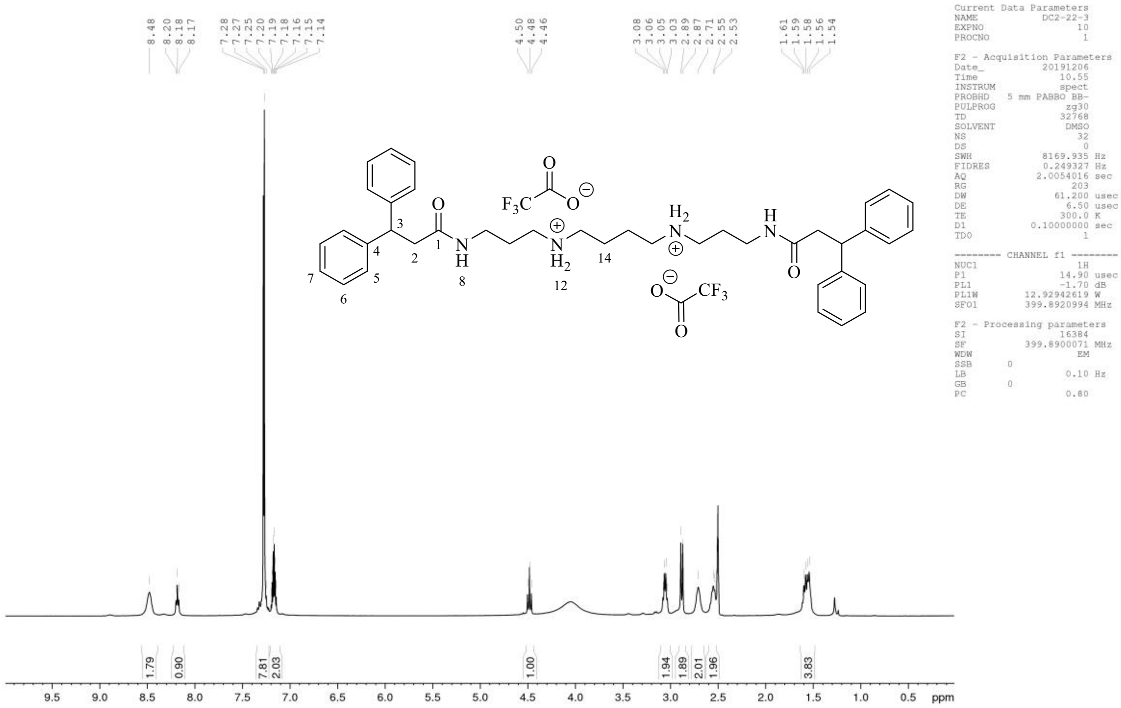

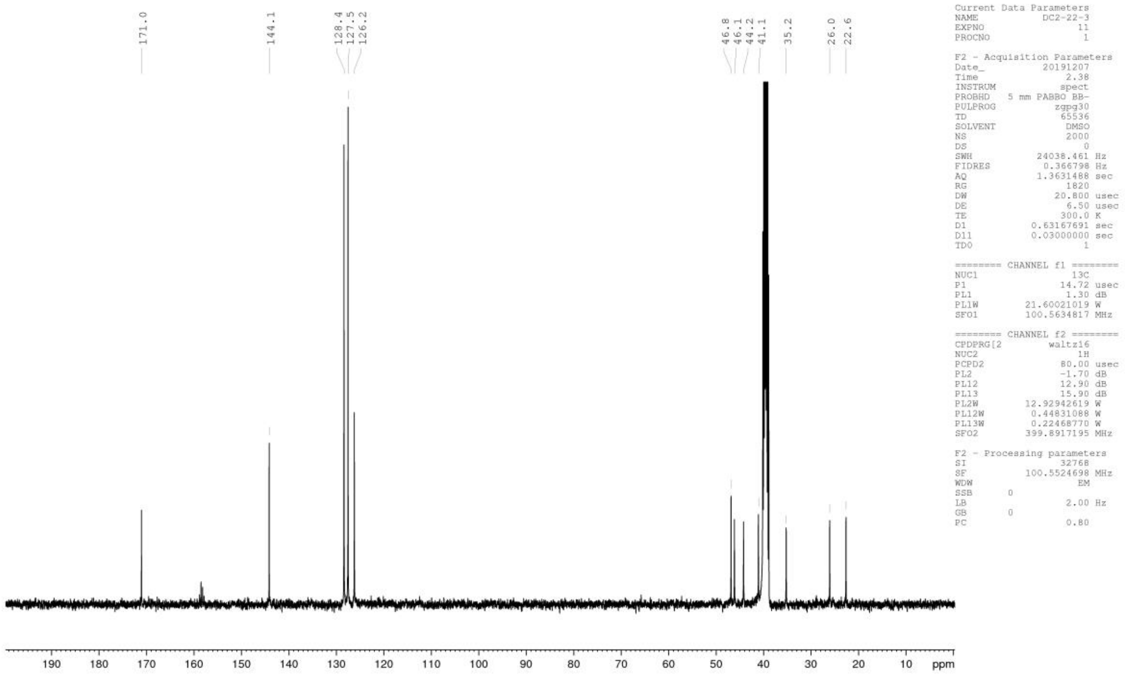

Figure **S21** ^{1}H NMR (DMSO-d_6, 400 MHz) and ^{13}C NMR (DMSO-d_6, 100 MHz) spectra for **15a**.

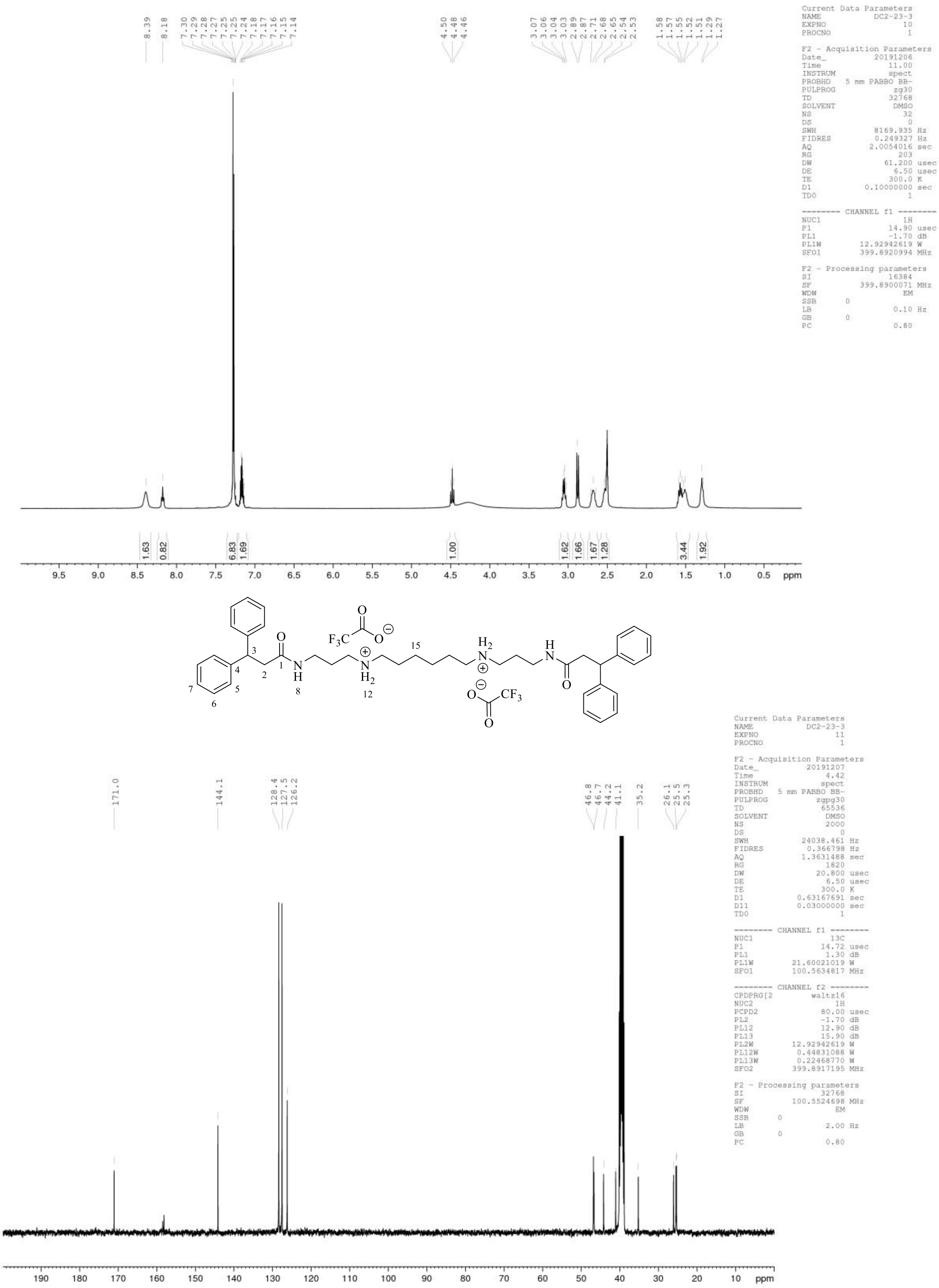

Figure **S22** ^{1}H NMR (DMSO-d_6, 400 MHz) and ^{13}C NMR (DMSO-d_6, 100 MHz) spectra for **15b**.

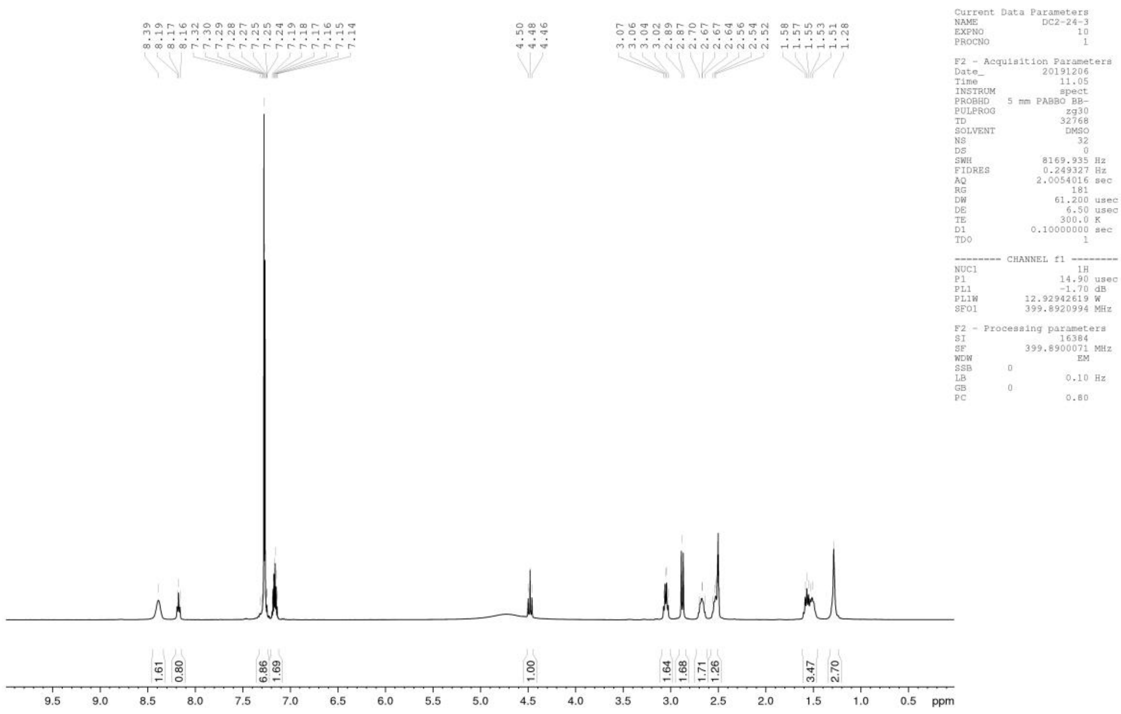

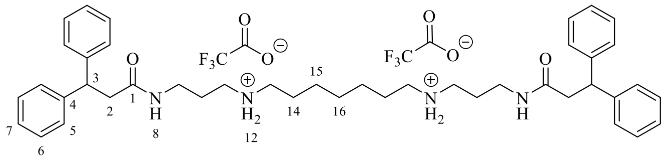

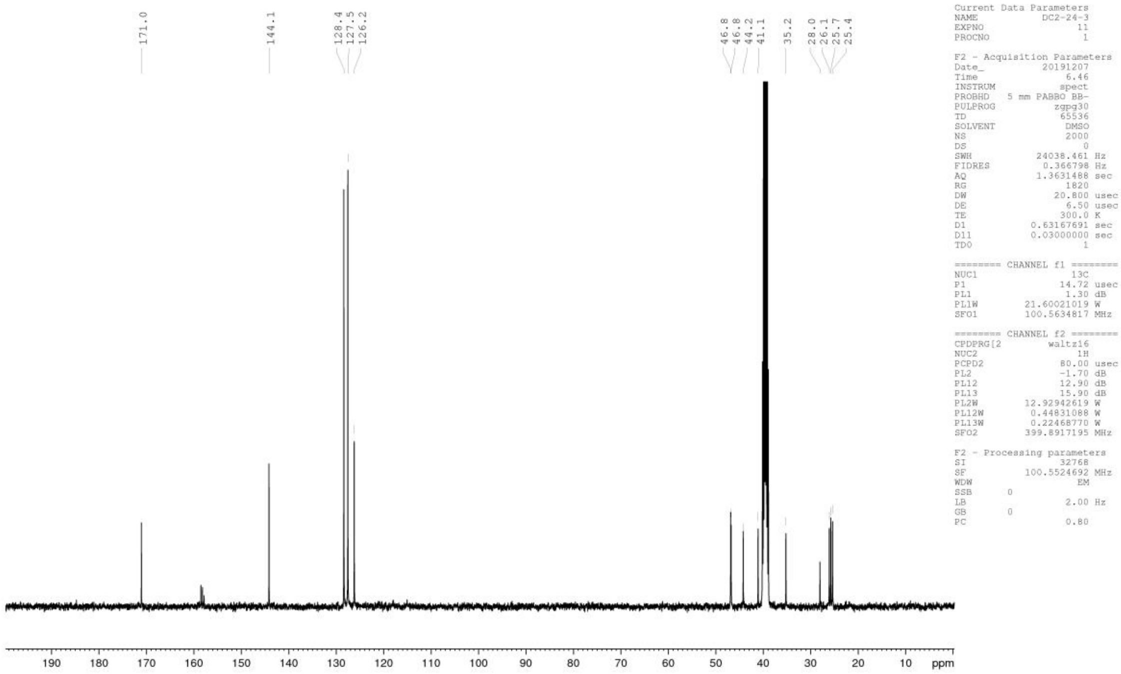

Figure **S23** ^{1}H NMR (DMSO-d_6, 400 MHz) and ^{13}C NMR (DMSO-d_6, 100 MHz) spectra for **15c**.

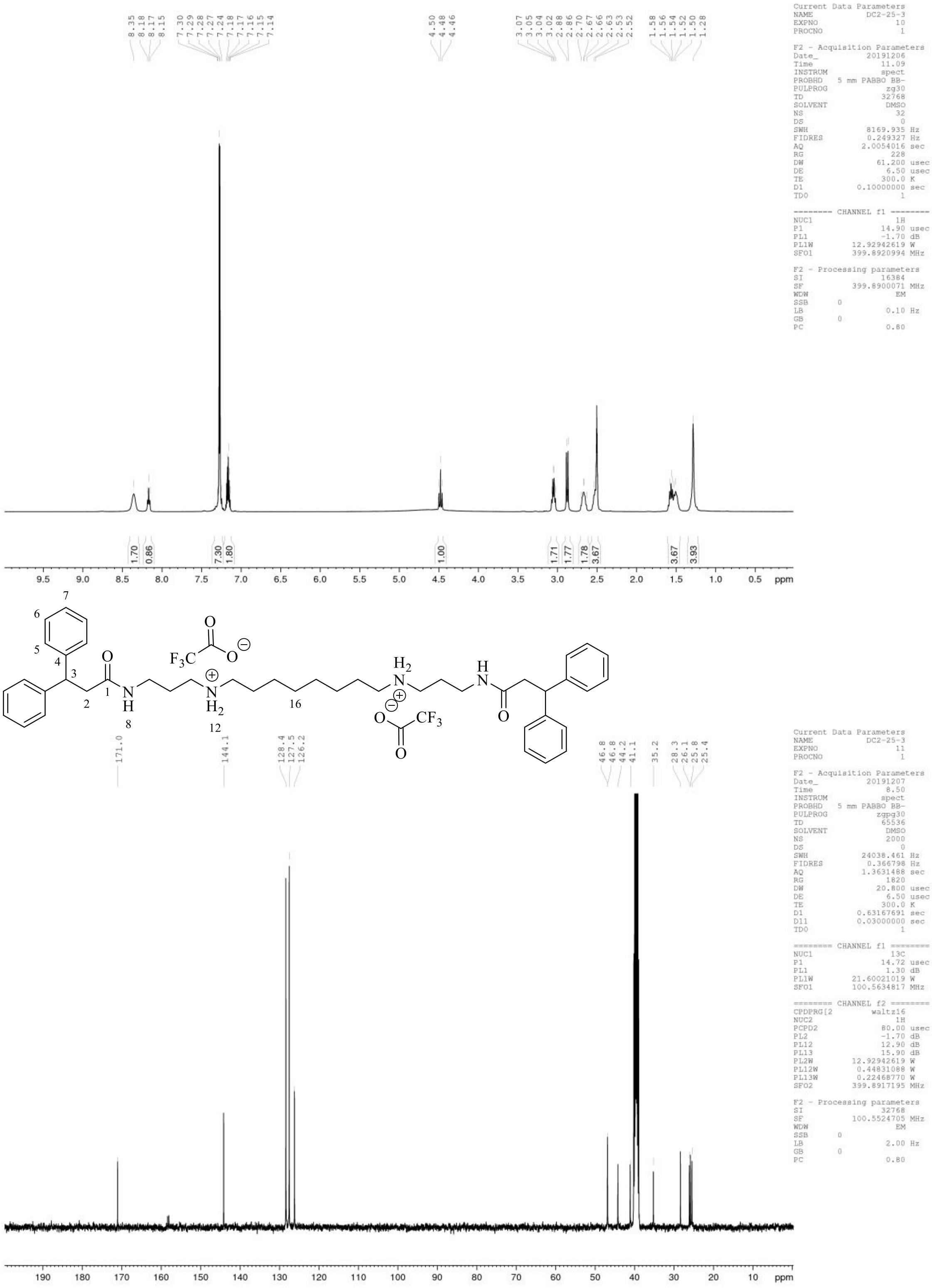

Figure **S24** ^{1}H NMR (DMSO-d_6, 400 MHz) and ^{13}C NMR (DMSO-d_6, 100 MHz) spectra for **15d**.

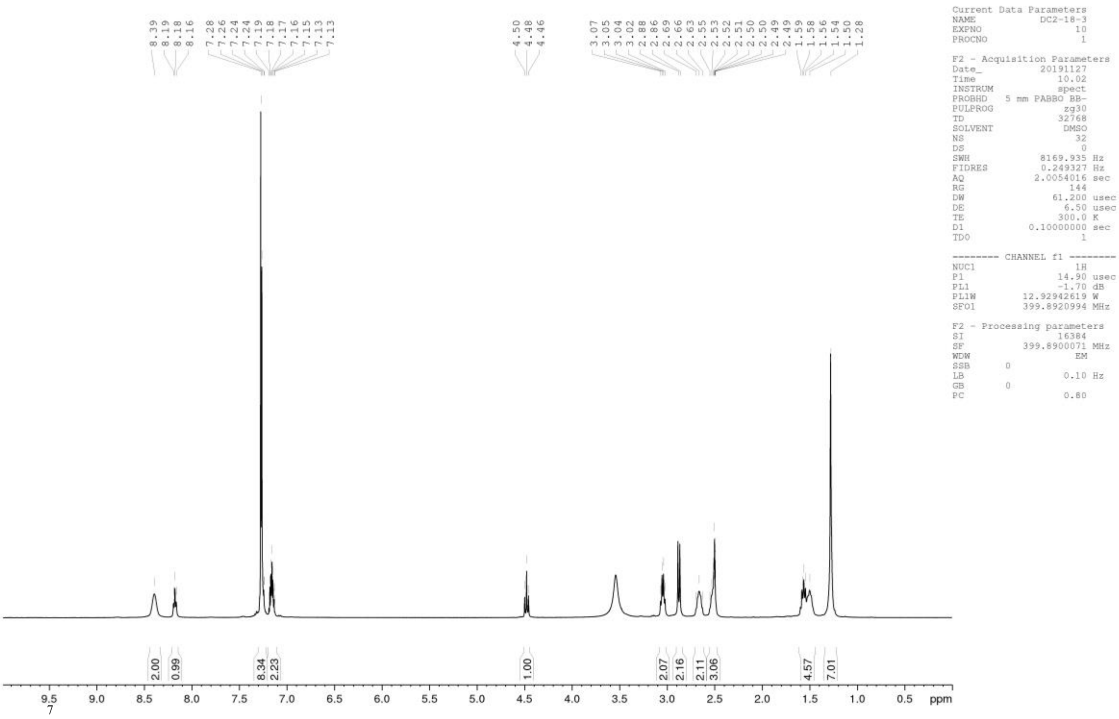

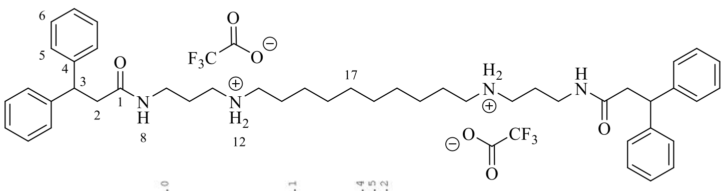

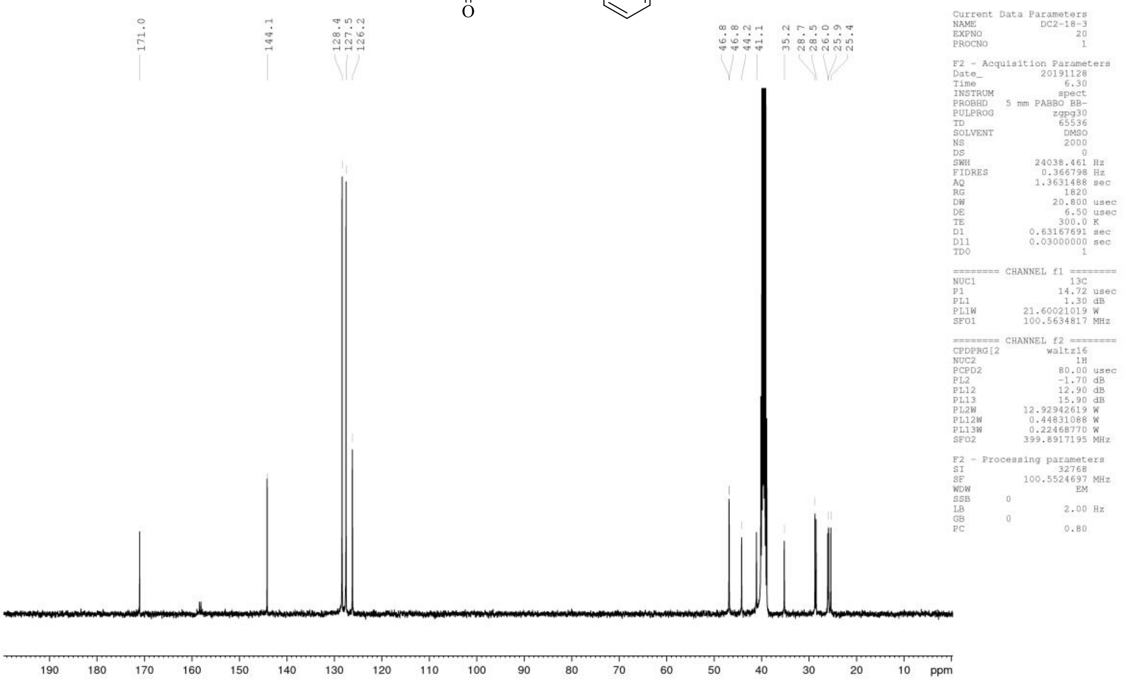

Figure **S25** ^{1}H NMR (DMSO-d_6, 400 MHz) and ^{13}C NMR (DMSO-d_6, 100 MHz) spectra for **15e**.

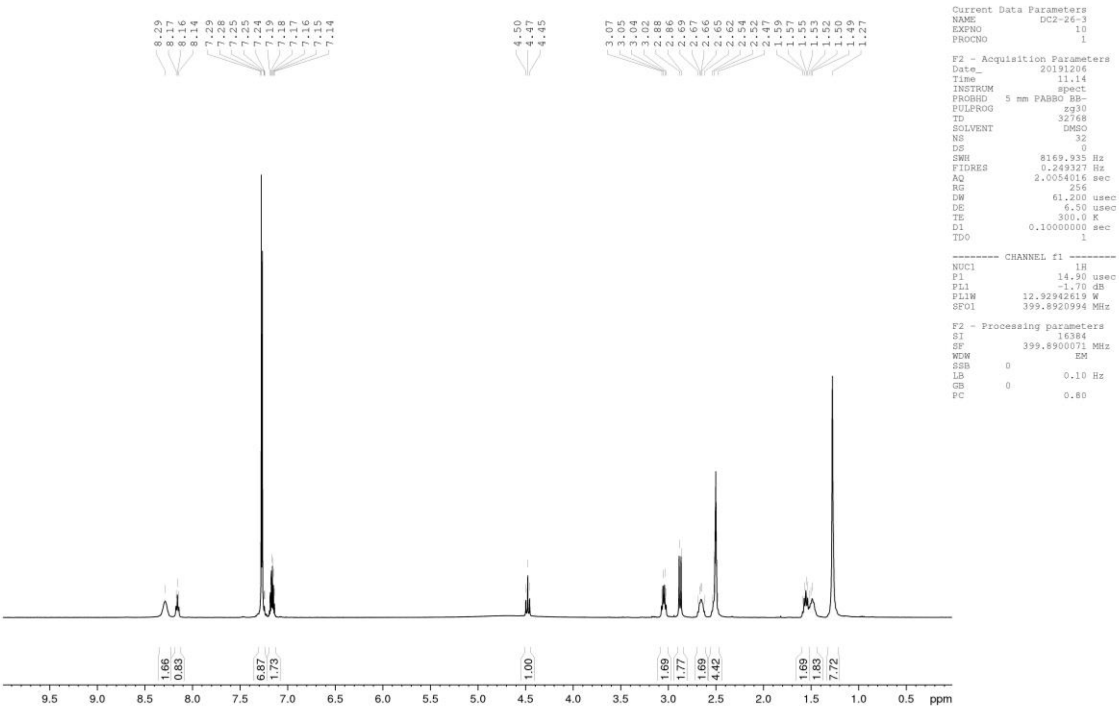

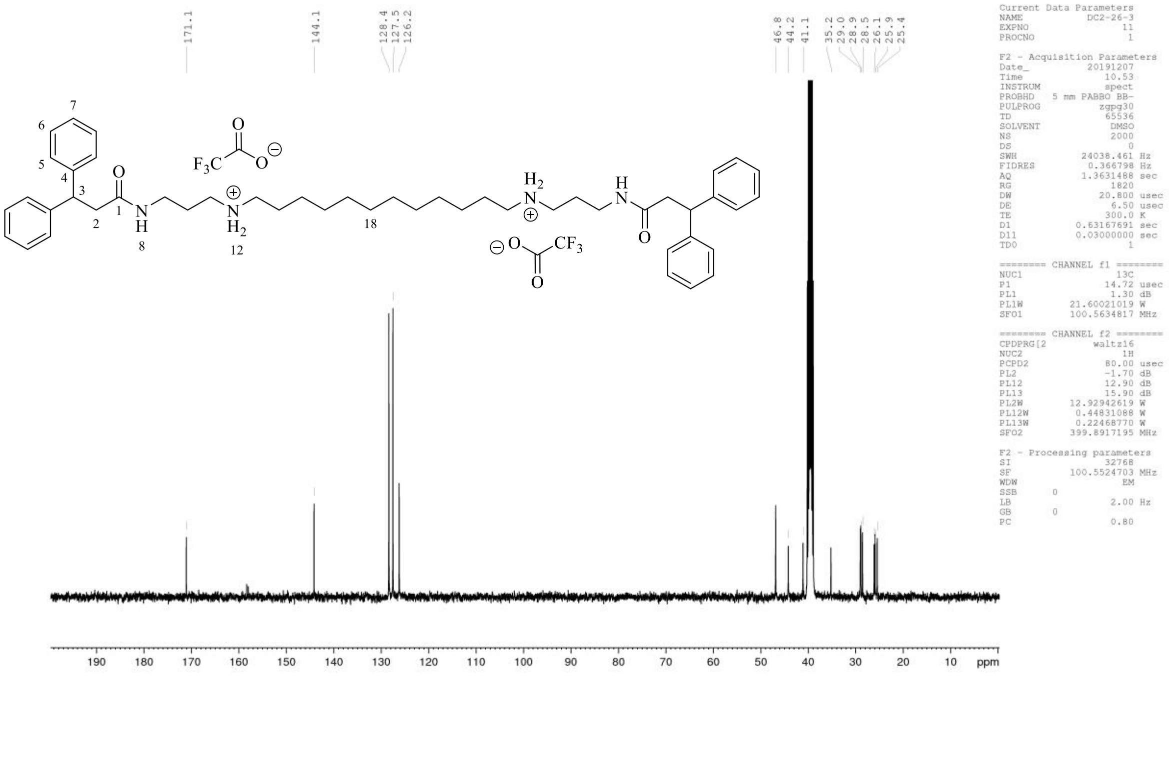

Figure **S26** ^{1}H NMR (DMSO-d_6, 400 MHz and ^{13}C NMR (DMSO-d_6, 100 MHz) spectra for **15f**.

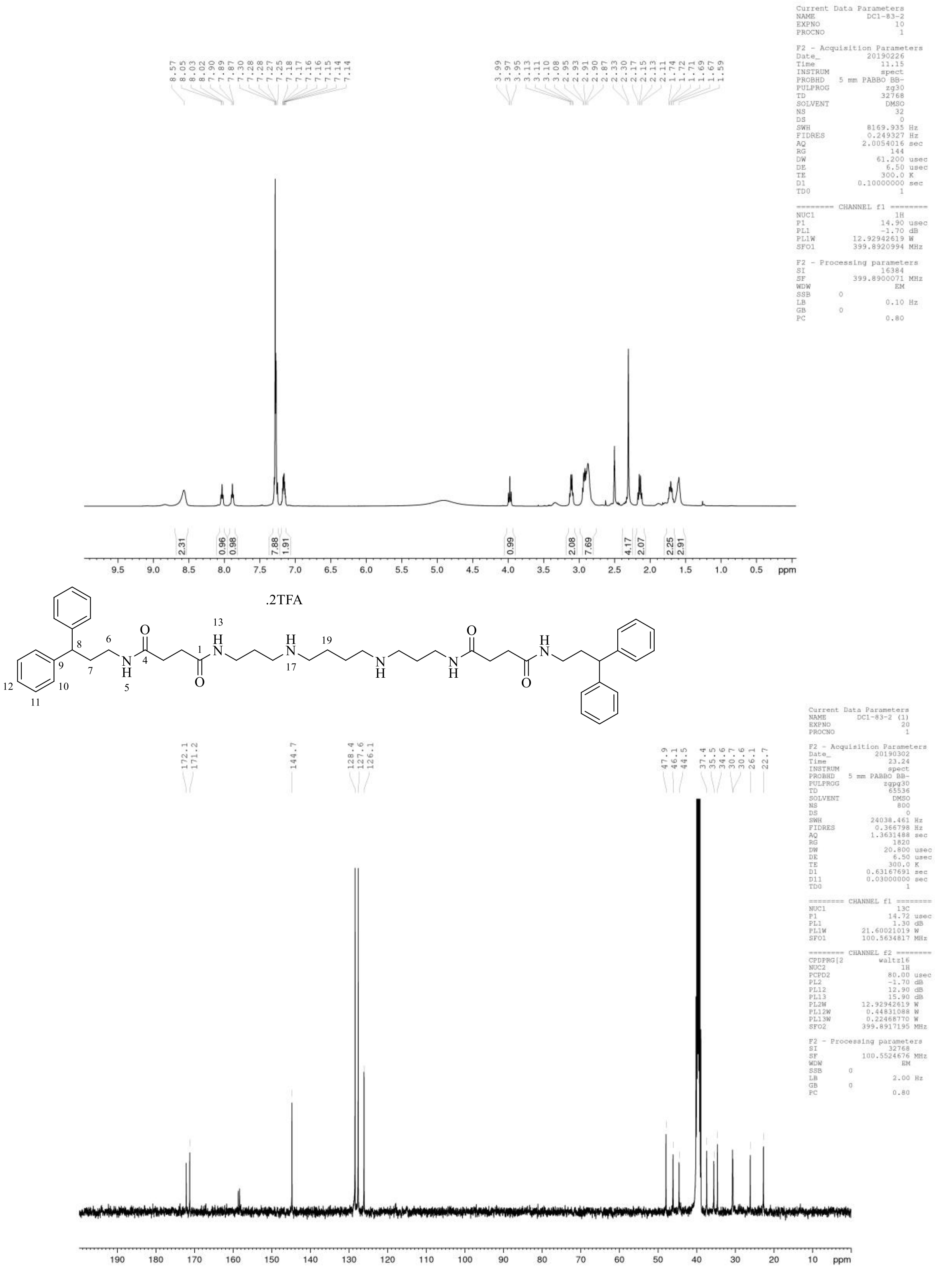

Figure **S27** ^{1}H NMR (DMSO-d_6, 400 MHz) and ^{13}C NMR (DMSO-d_6, 100 MHz) spectra for **16a**.

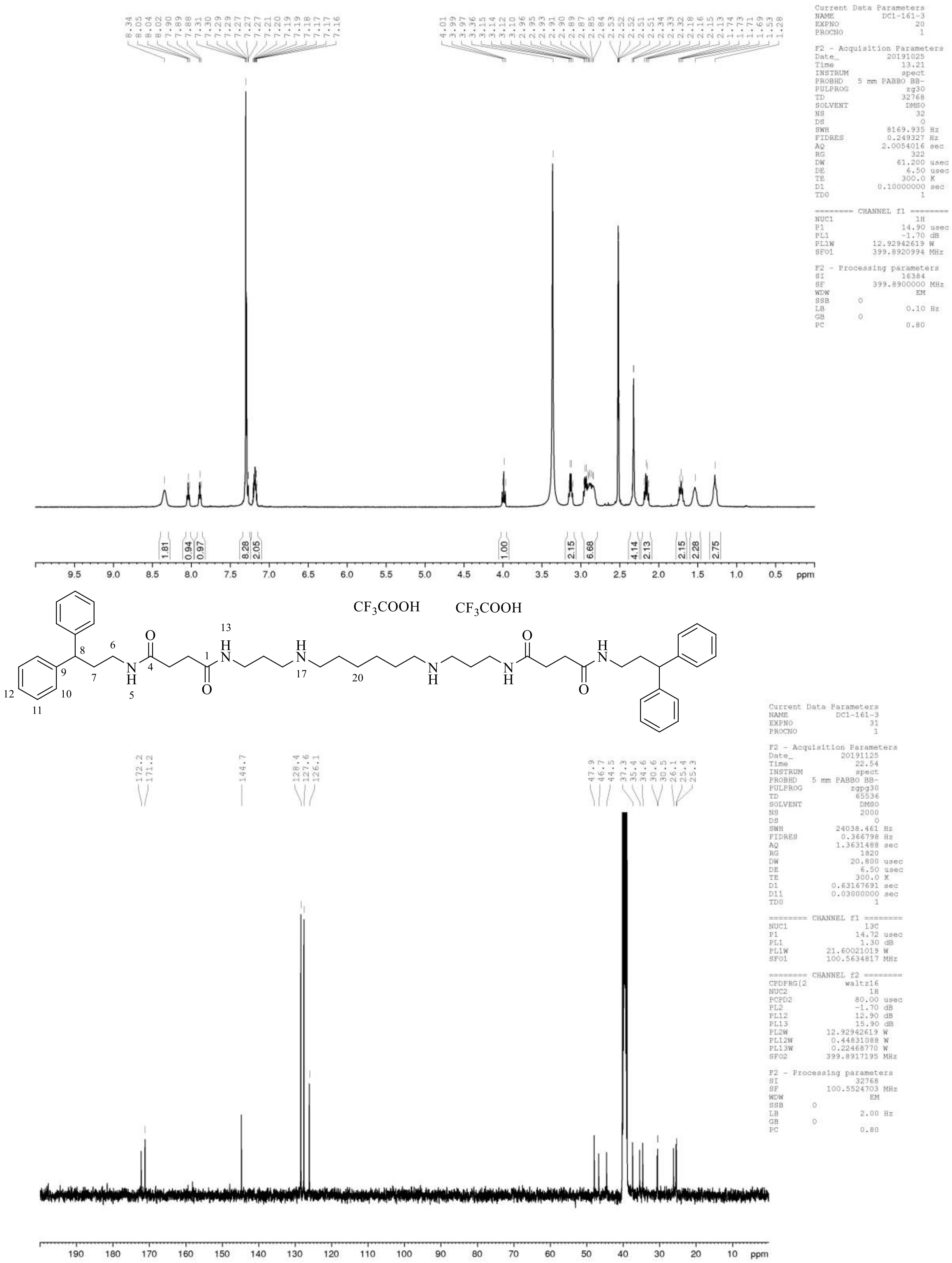

Figure **S28** ^{1}H NMR (DMSO-d_6, 400 MHz) and ^{13}C NMR (DMSO-d_6, 100 MHz) spectra for **16b**.

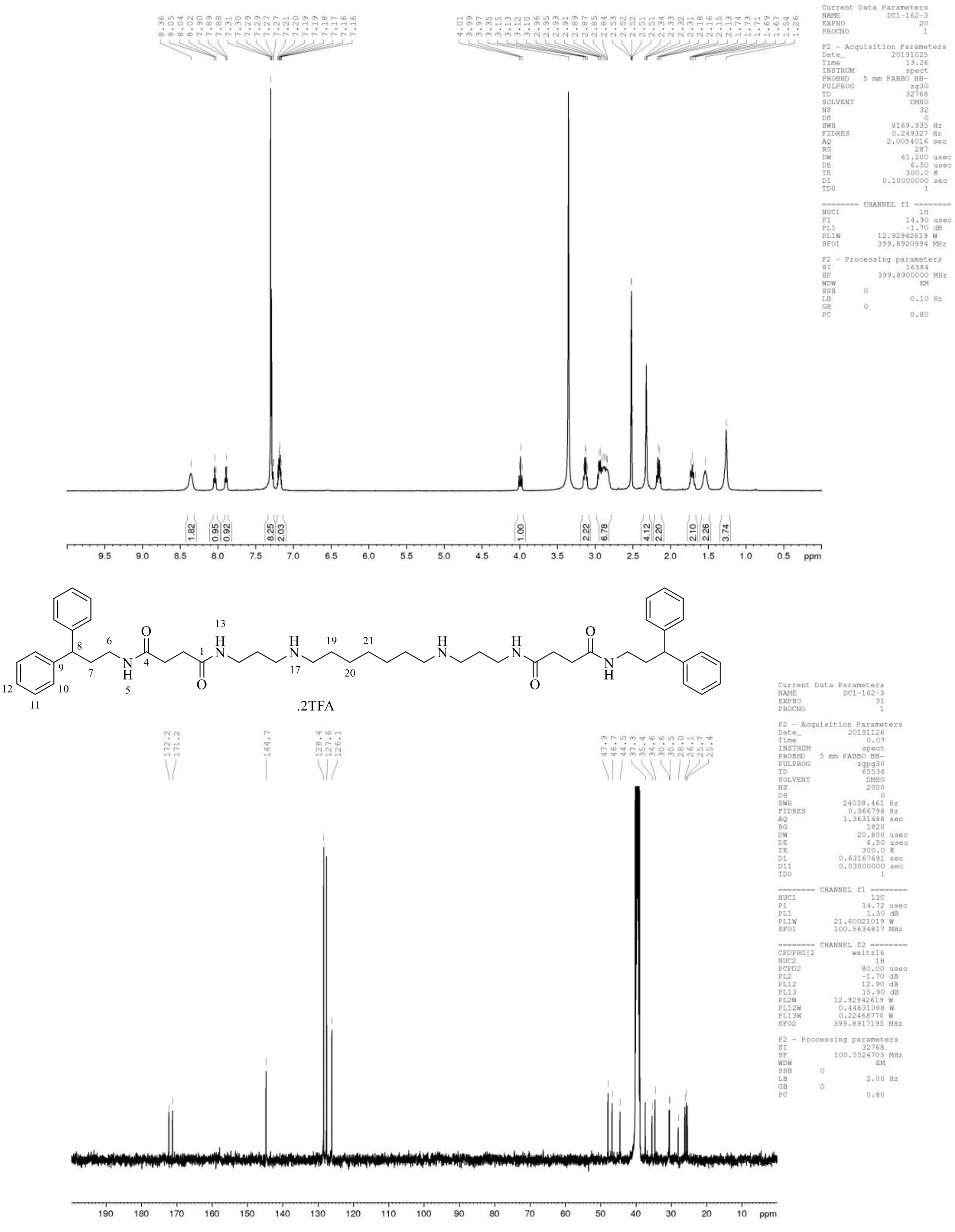

Figure **S29** ^{1}H NMR (DMSO-d_6, 400 MHz) and ^{13}C NMR (DMSO-d_6, 100 MHz) spectra for **16c**.

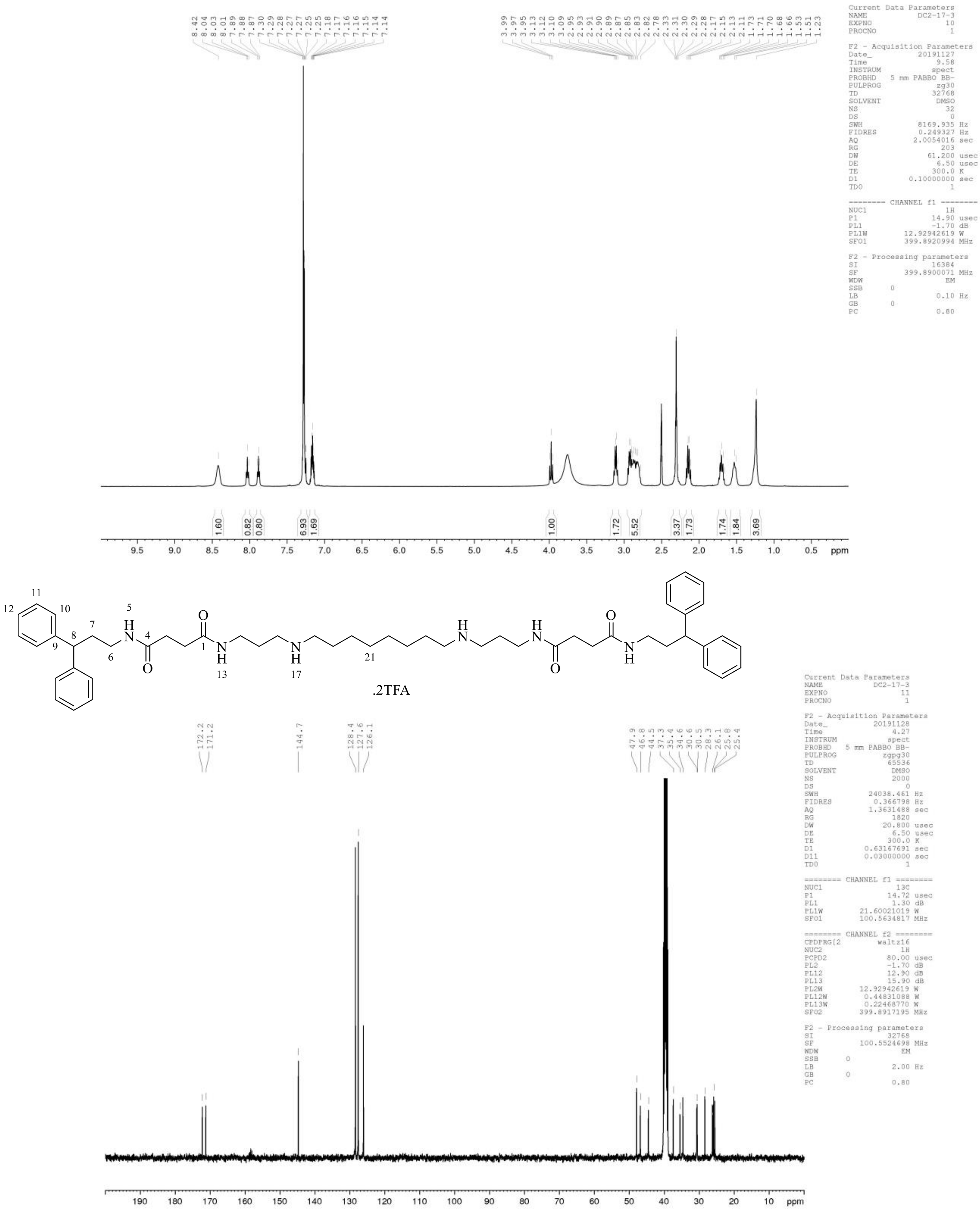

Figure **S30** ^{1}H NMR (DMSO-d_6, 400 MHz) and ^{13}C NMR (DMSO-d_6, 100 MHz) spectra for **16d**.

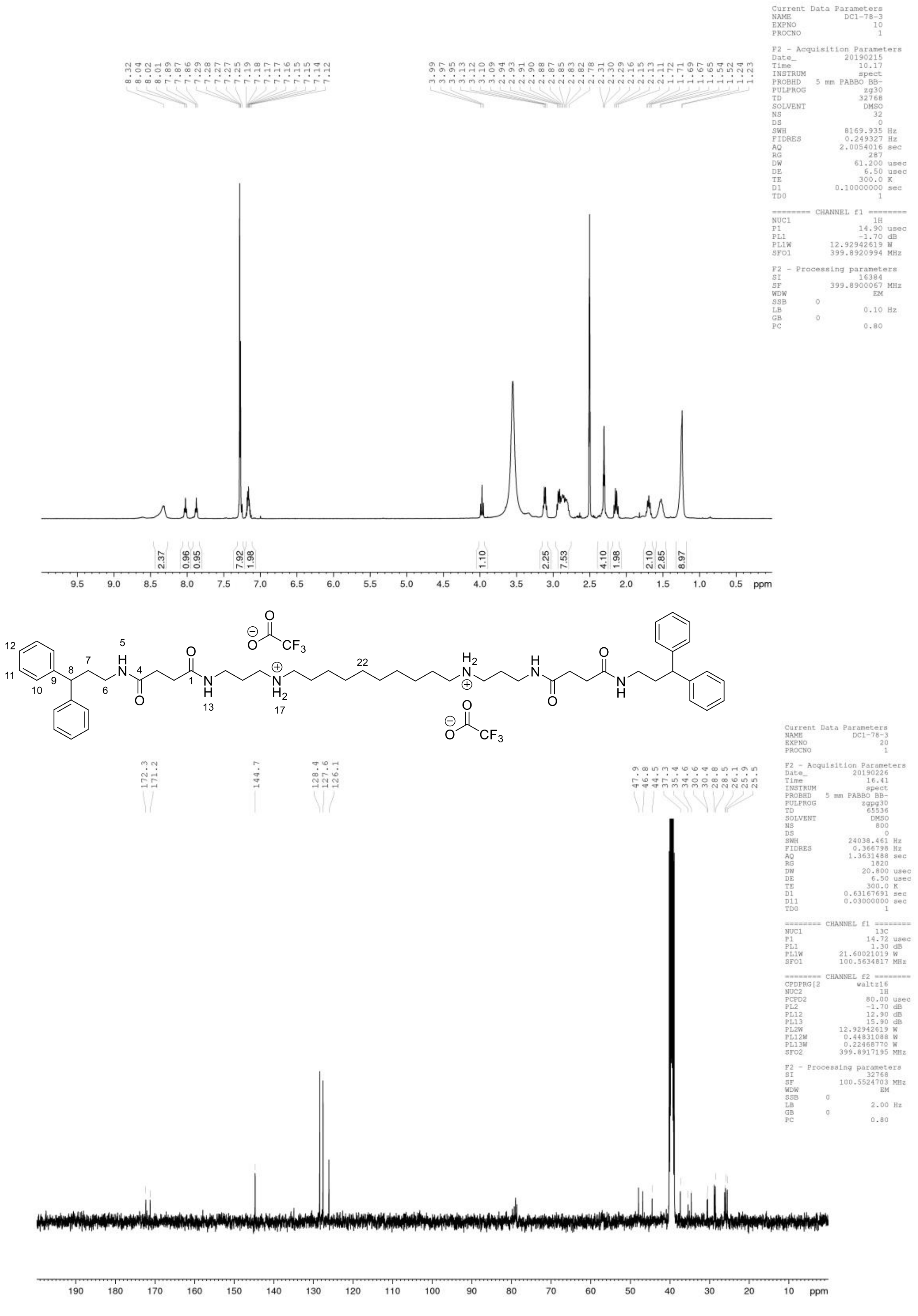

Figure **S31** ^{1}H NMR (DMSO-d_6, 400 MHz) and ^{13}C NMR (DMSO-d_6, 100 MHz) spectra for **16e**.

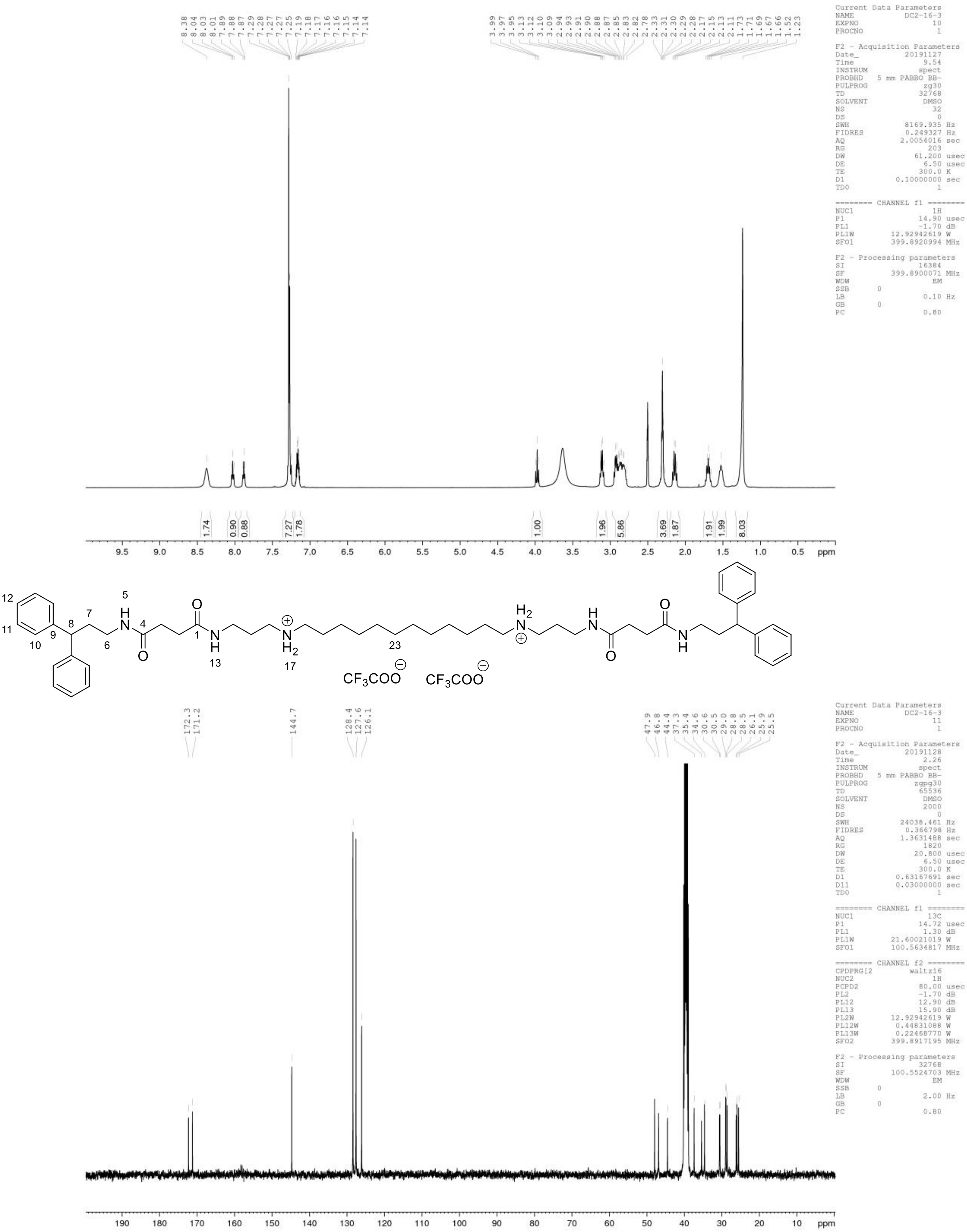

Figure **S32** ^{1}H NMR (DMSO-d_6, 400 MHz) and ^{13}C NMR (DMSO-d_6, 100 MHz) spectra for **16f**.

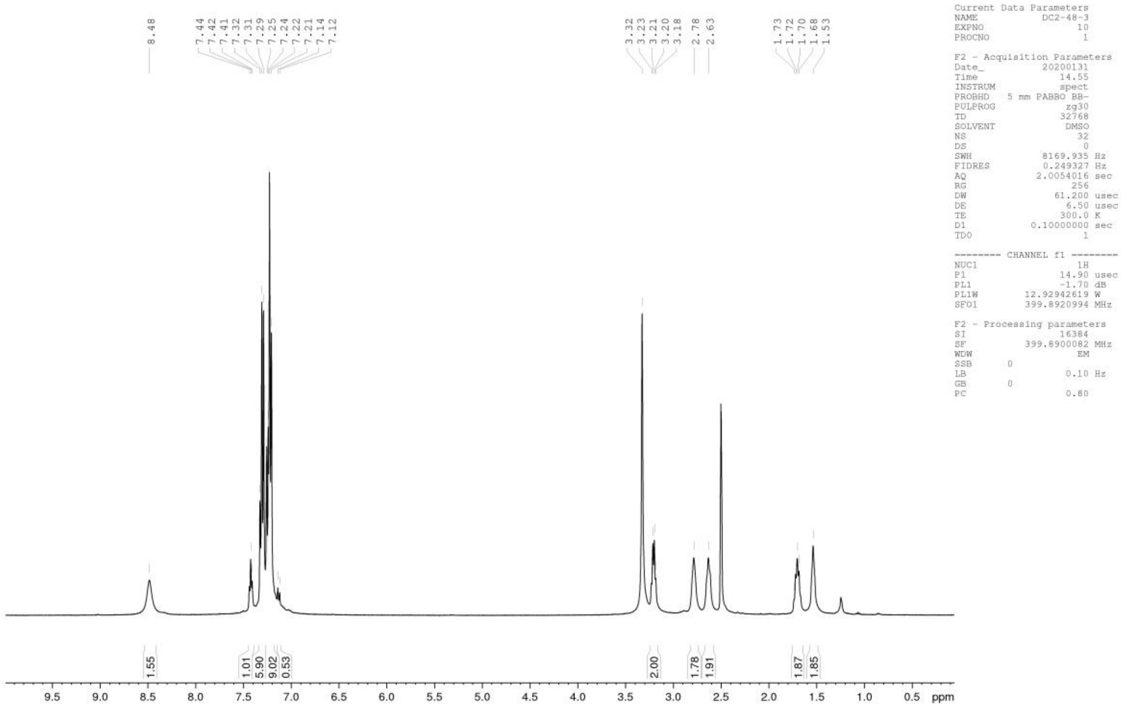

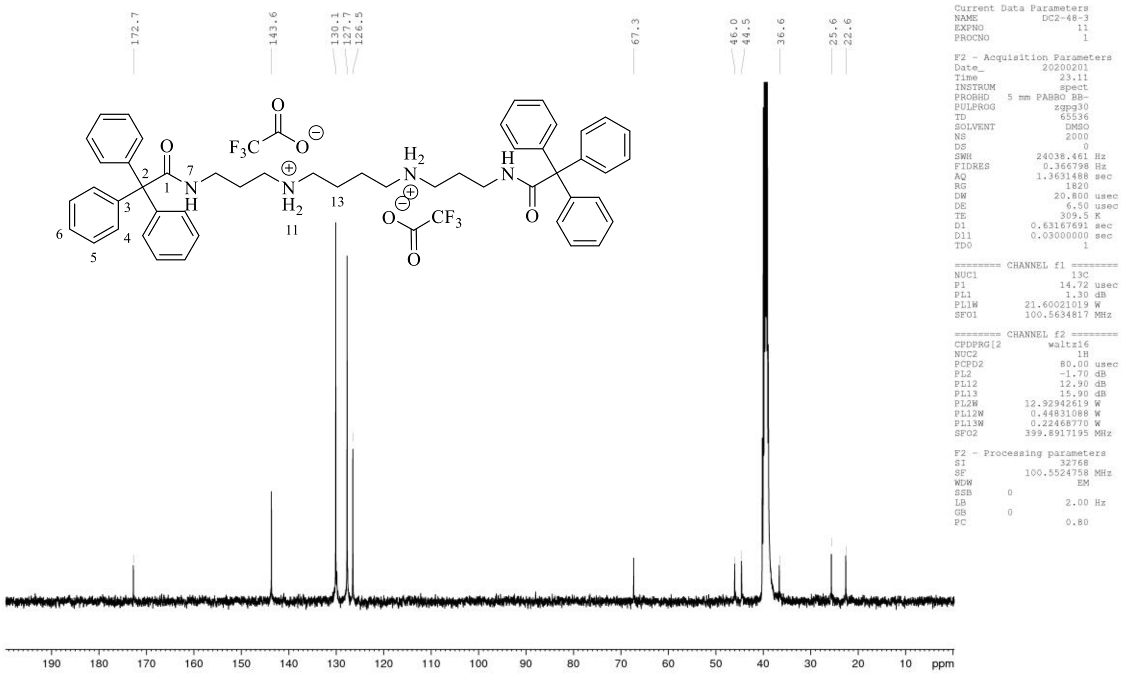

Figure **S33** ^{1}H NMR (DMSO-d_6, 400 MHz) and ^{13}C NMR (DMSO-d_6, 100 MHz) spectra for **17a**.

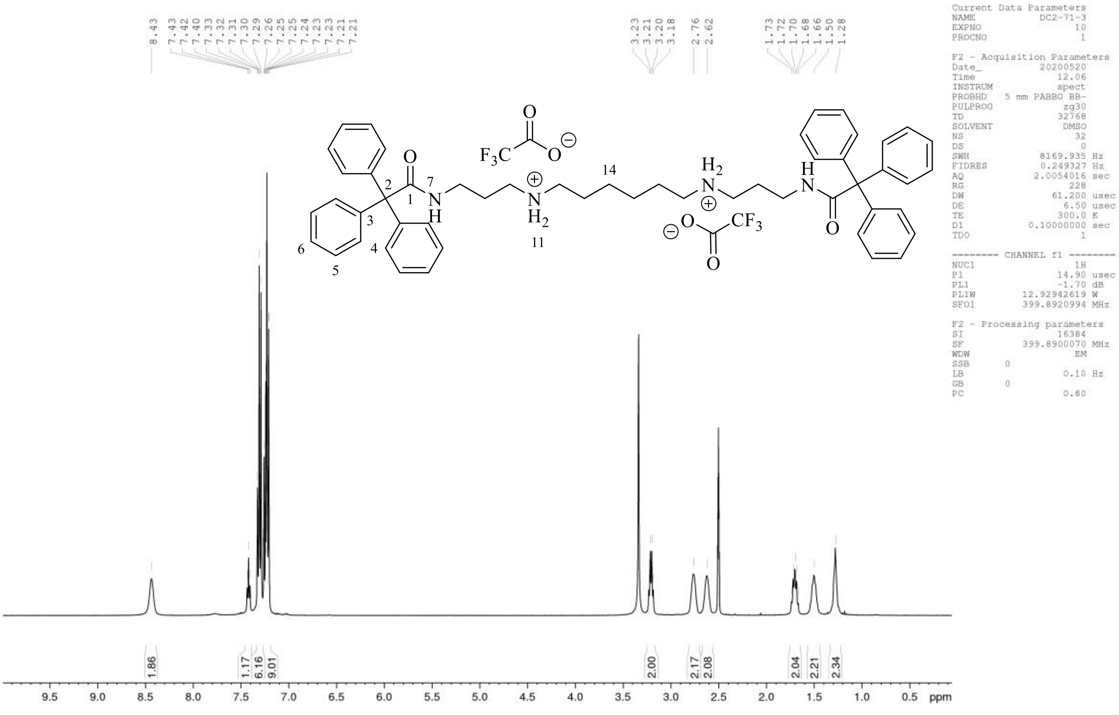

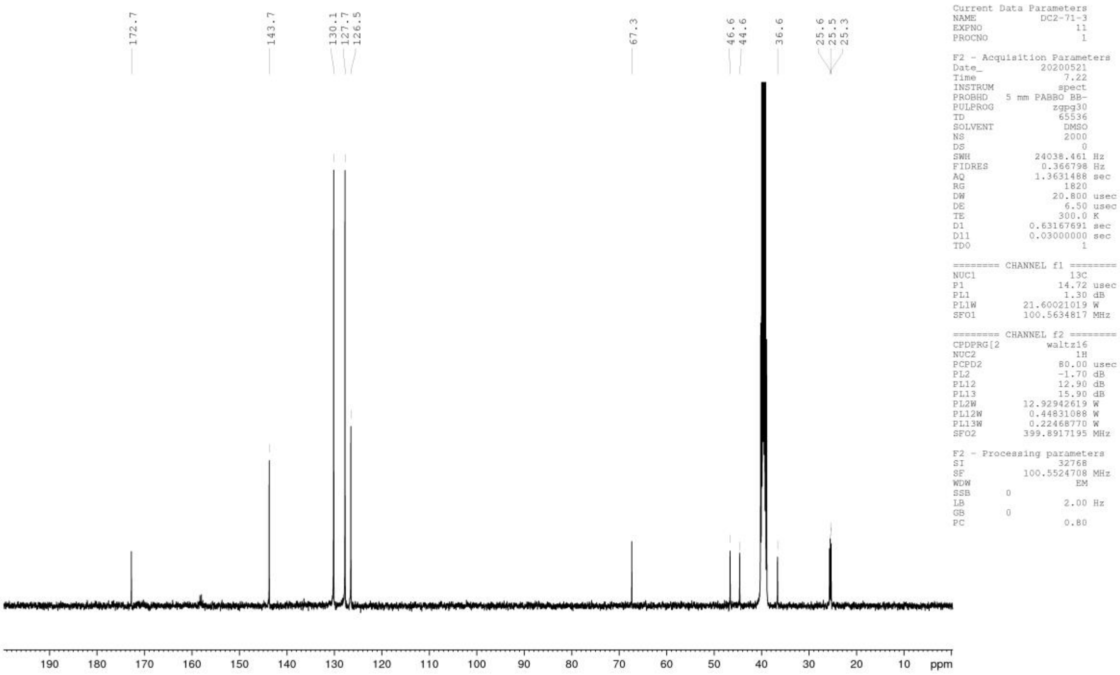

Figure **S34** ^{1}H NMR (DMSO-d_6, 400 MHz) and ^{13}C NMR (DMSO-d_6, 100 MHz) spectra for **17b**.

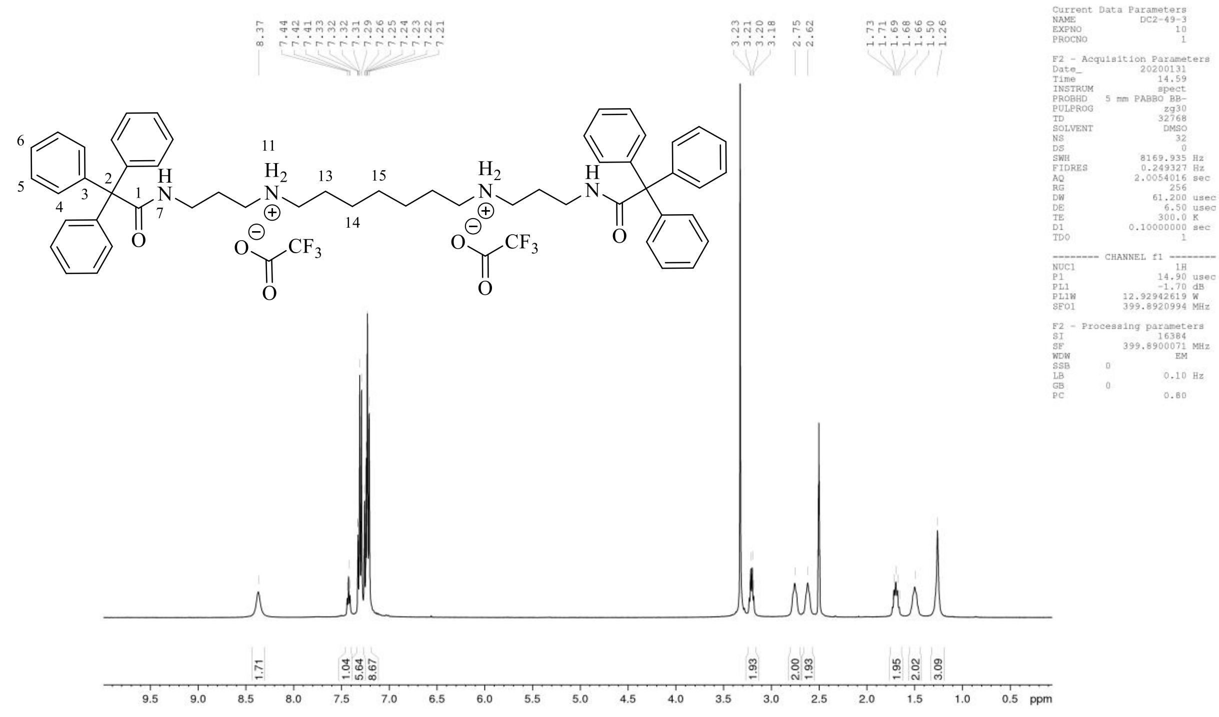

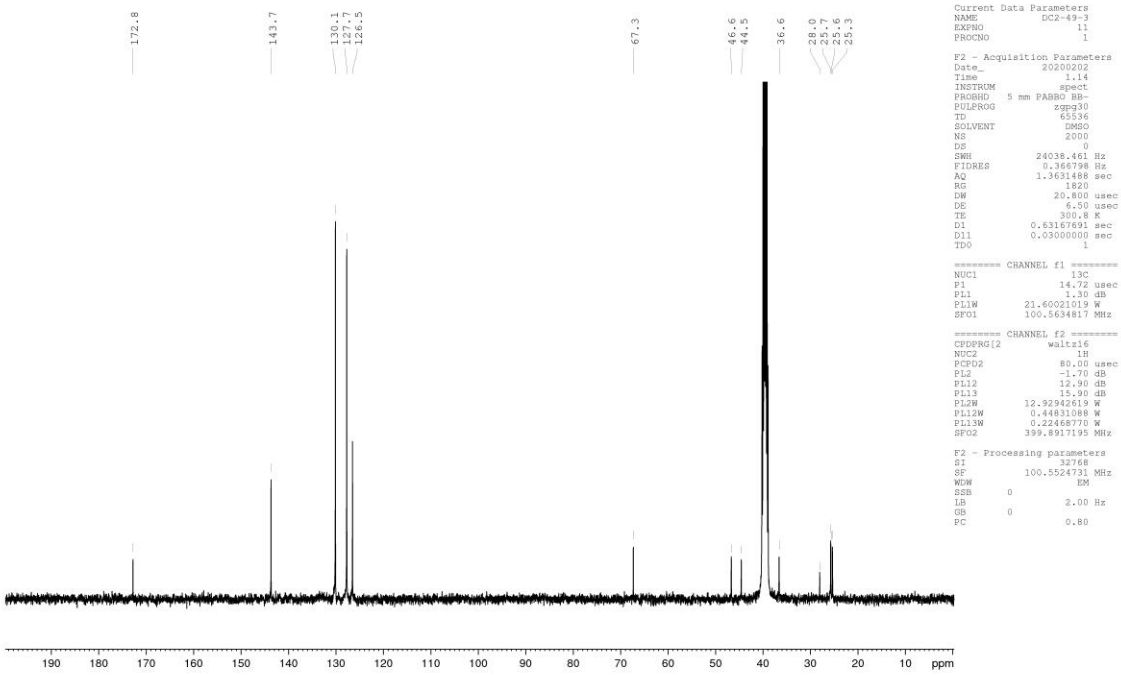

Figure **S35** ^{1}H NMR (DMSO-d_6, 400 MHz) and ^{13}C NMR (DMSO-d_6, 100 MHz) spectra for **17c**.

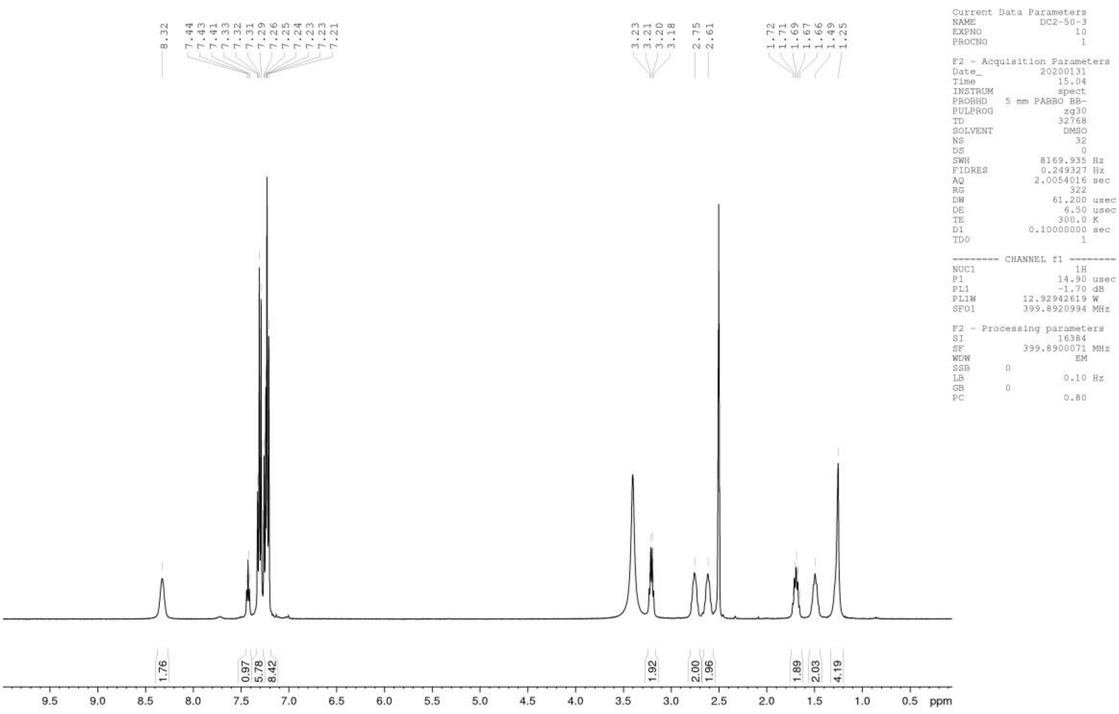

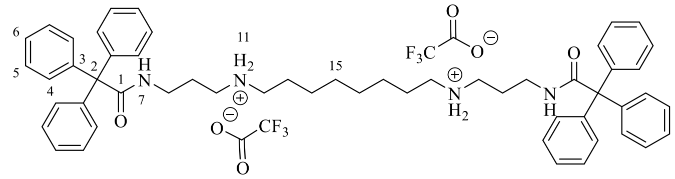

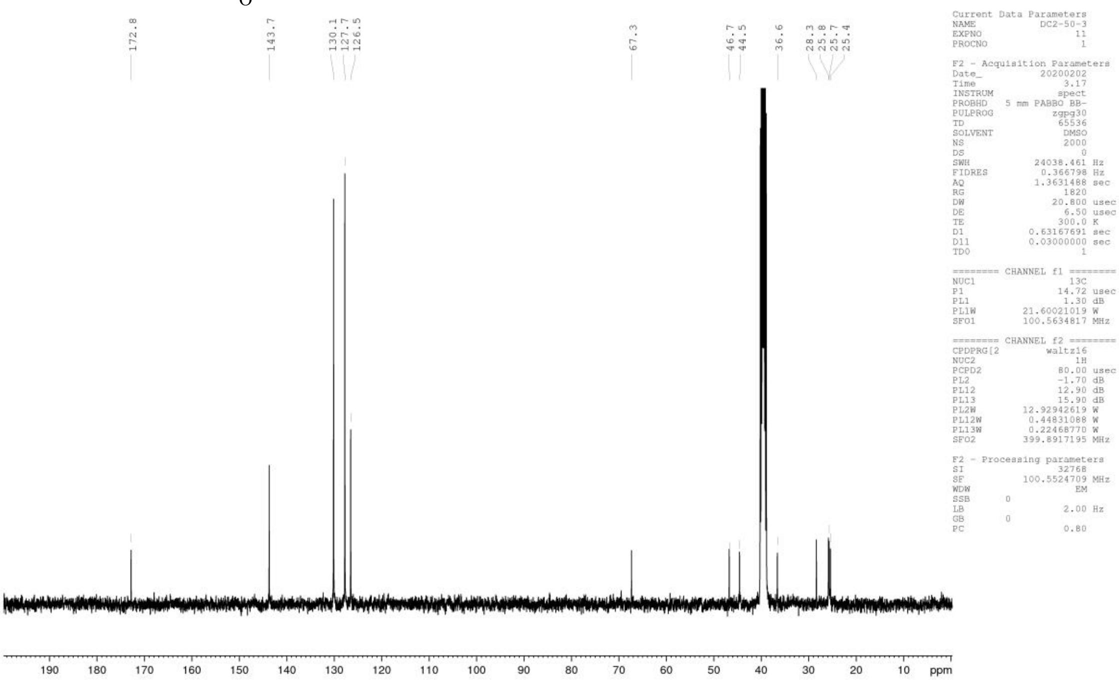

Figure **S36** ^{1}H NMR (DMSO-d_6, 400 MHz) and ^{13}C NMR (DMSO-d_6, 100 MHz) spectra for **17d**.

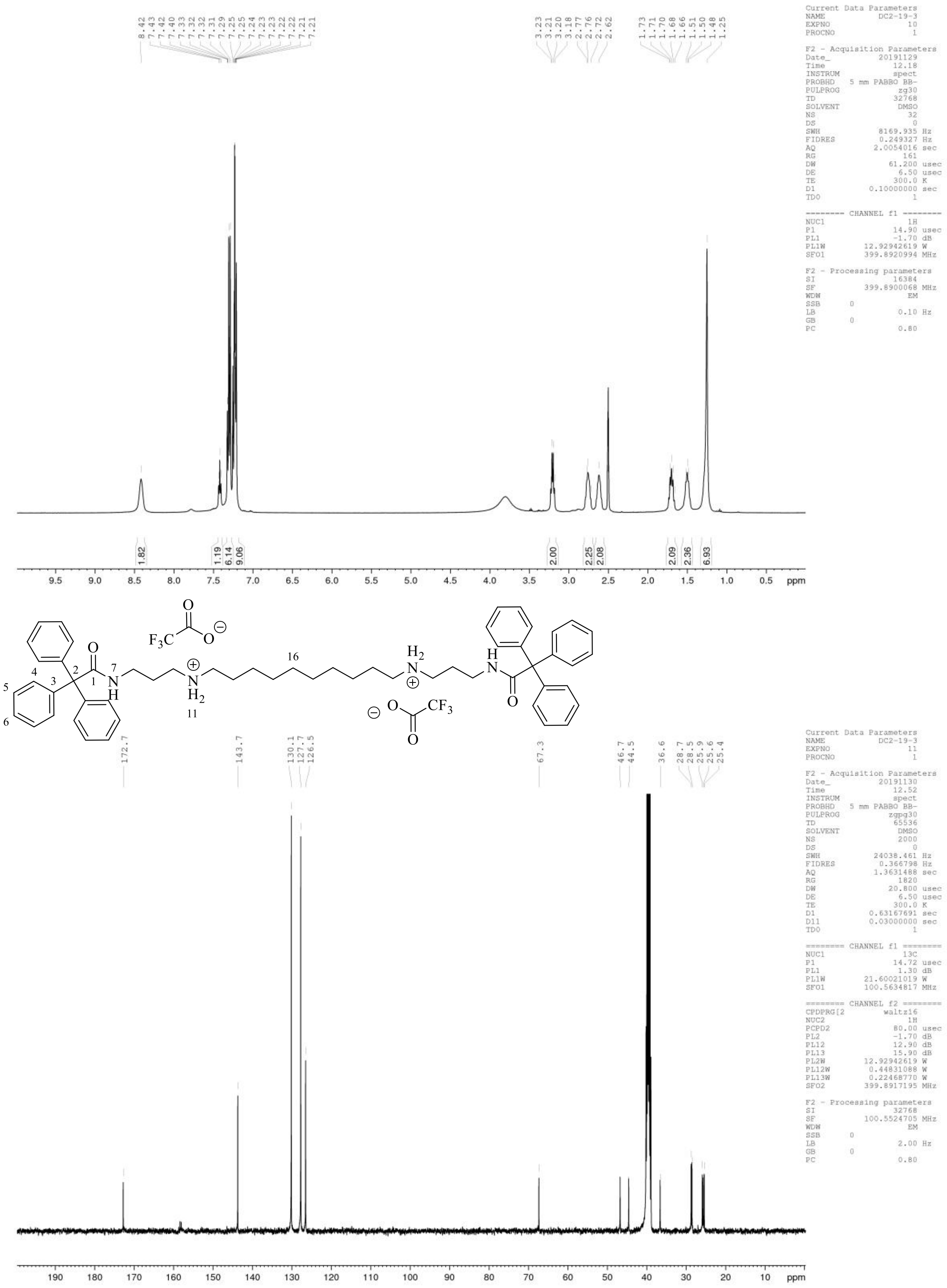

Figure **S37** ^{1}H NMR (DMSO-d_6, 400 MHz) and ^{13}C NMR (DMSO-d_6, 100 MHz) spectra for **17e**.

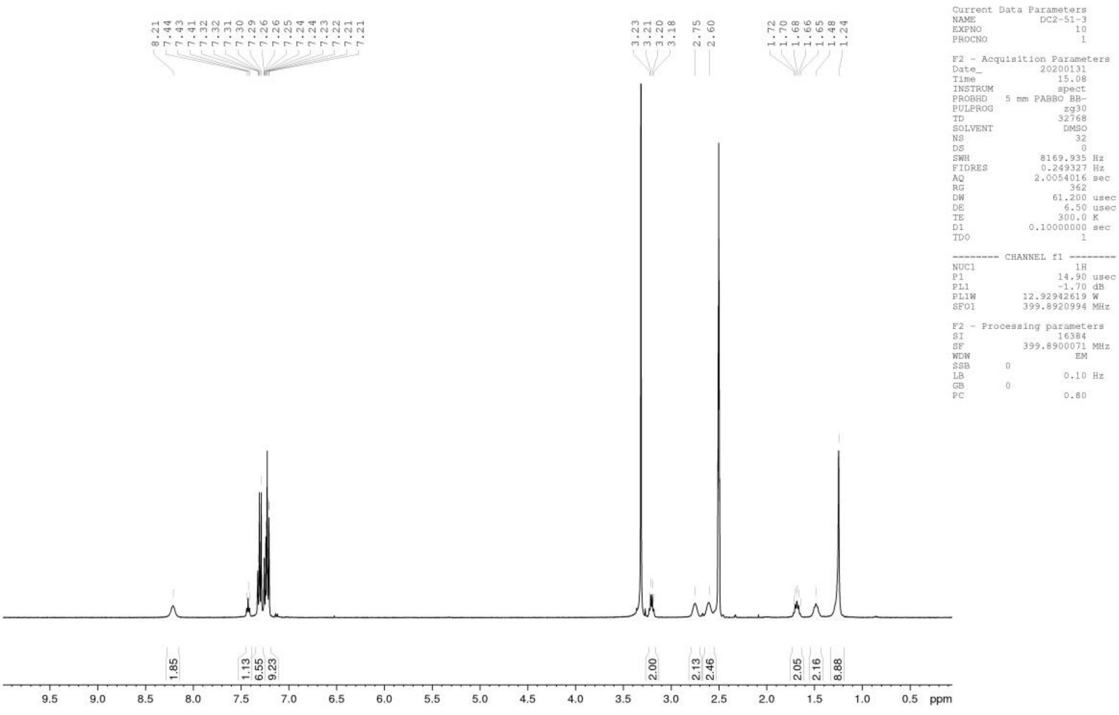

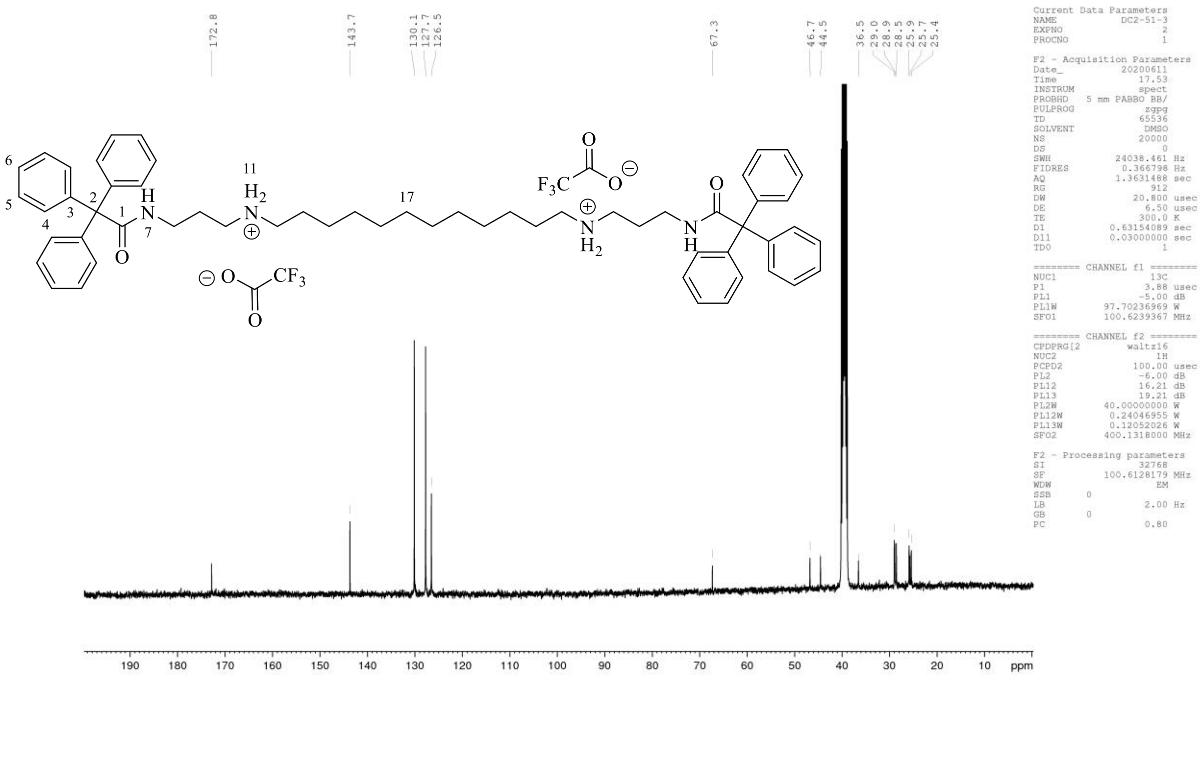

Figure **S38** ^{1}H NMR (DMSO-d_6, 400 MHz) and ^{13}C NMR (DMSO-d_6, 100 MHz) spectra for **17f**.

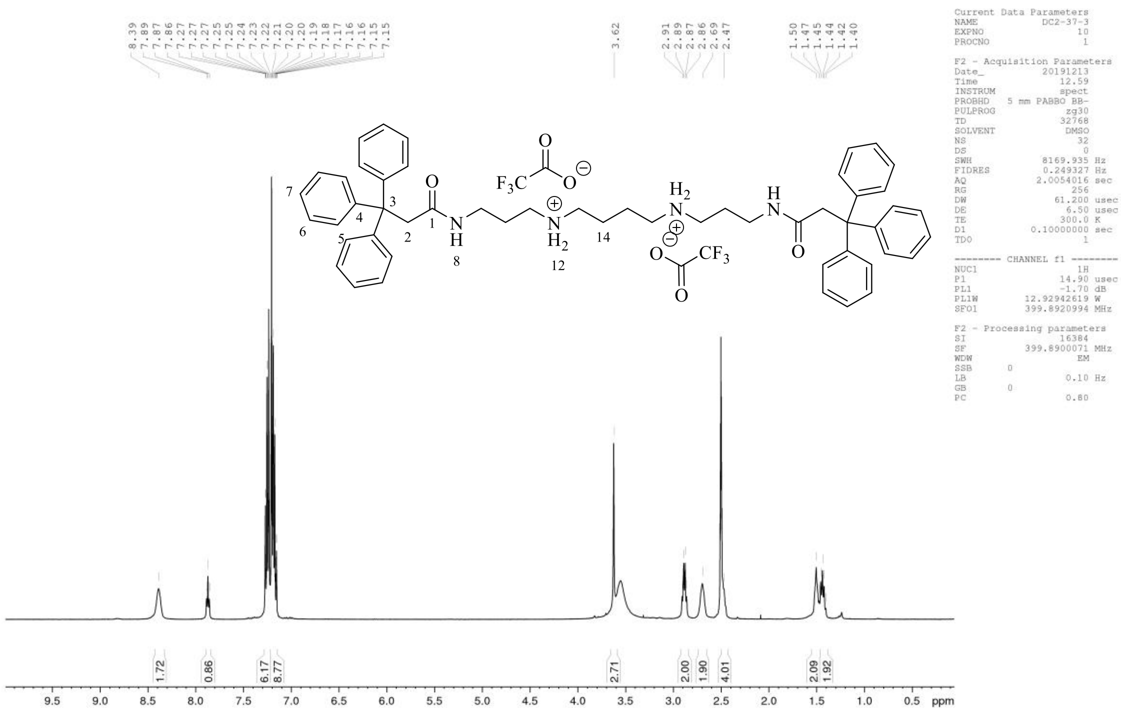

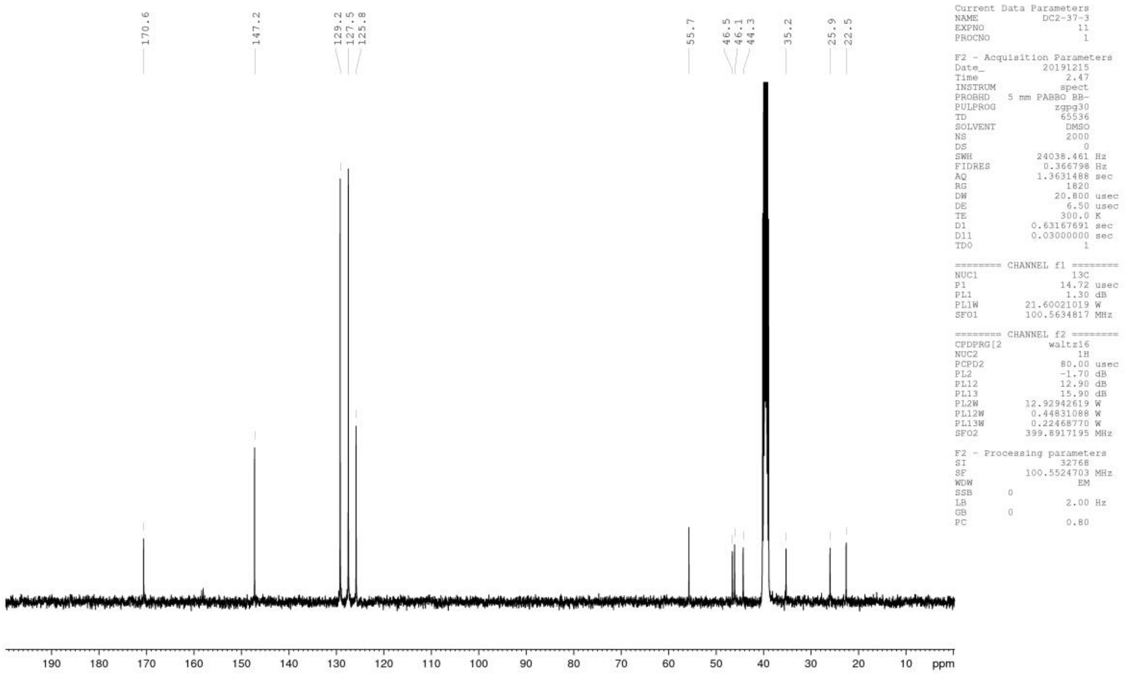

Figure **S39** ^{1}H NMR (DMSO-d_6, 400 MHz) and ^{13}C NMR (DMSO-d_6, 100 MHz) spectra for **18a**.

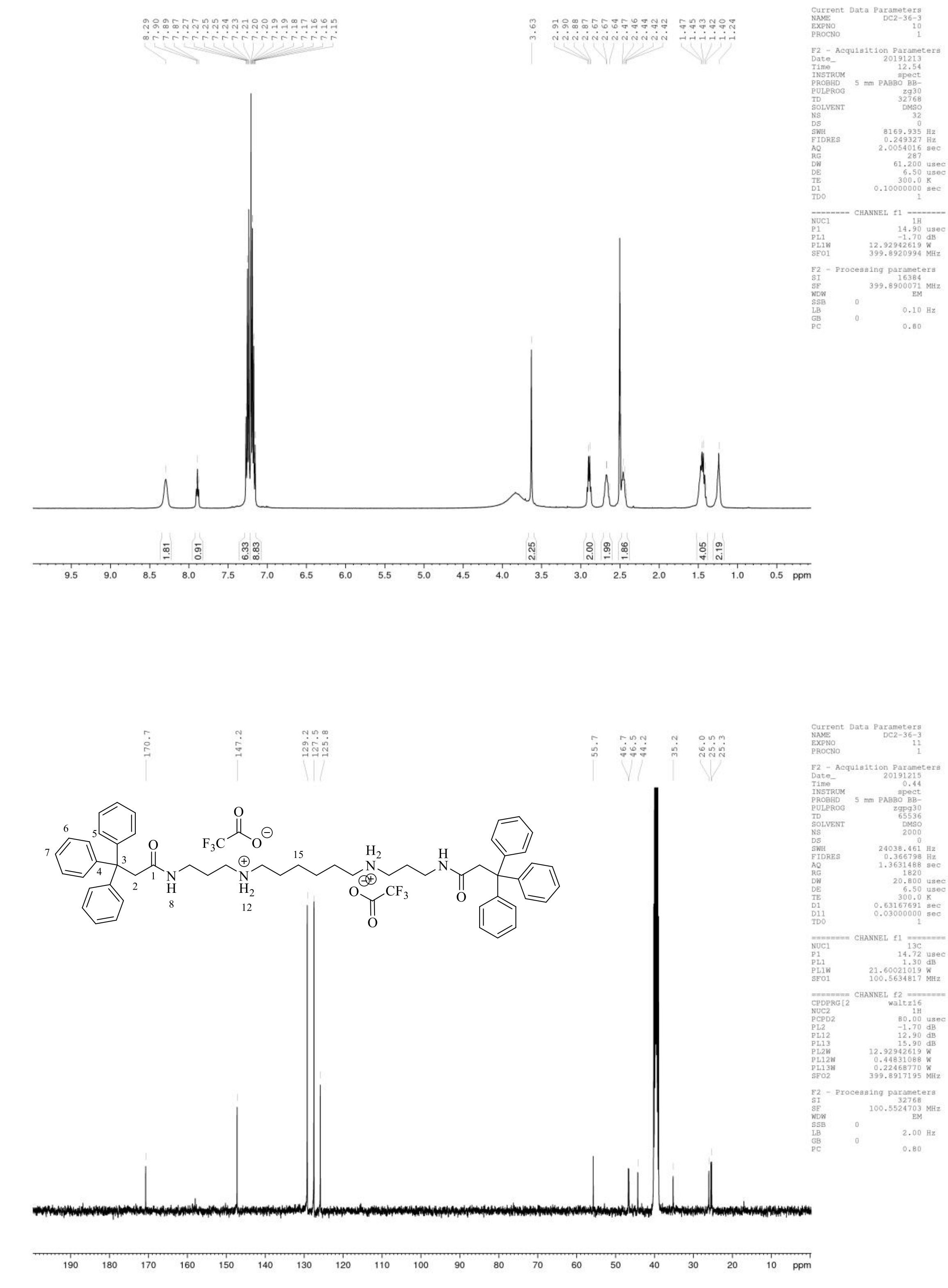

Figure **S40** ^{1}H NMR (DMSO-d_6, 400 MHz) and ^{13}C NMR (DMSO-d_6, 100 MHz) spectra for **18b**.

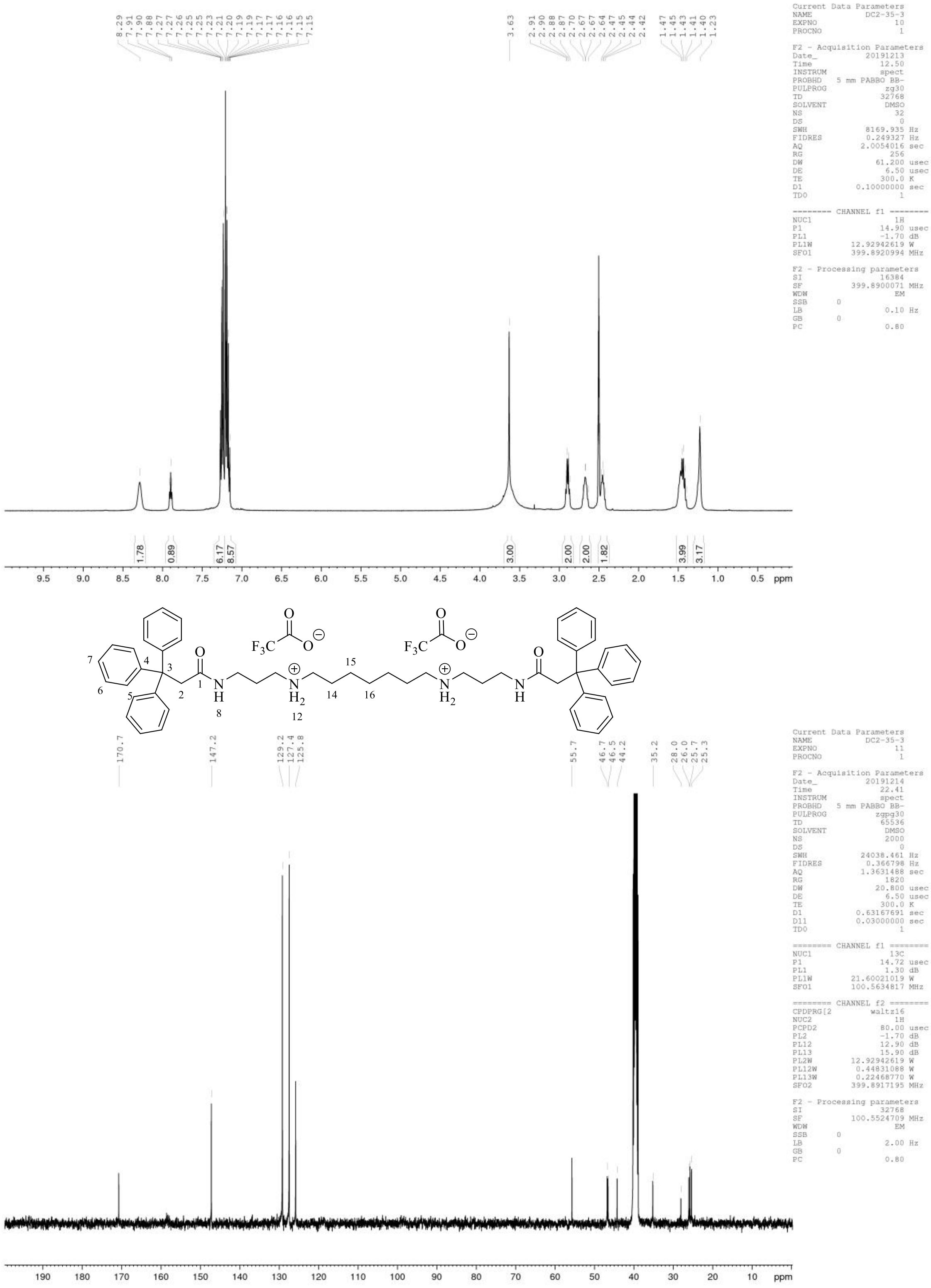

Figure **S41** ^{1}H NMR (DMSO-d_6, 400 MHz) and ^{13}C NMR (DMSO-d_6, 100 MHz) spectra for **18c**.

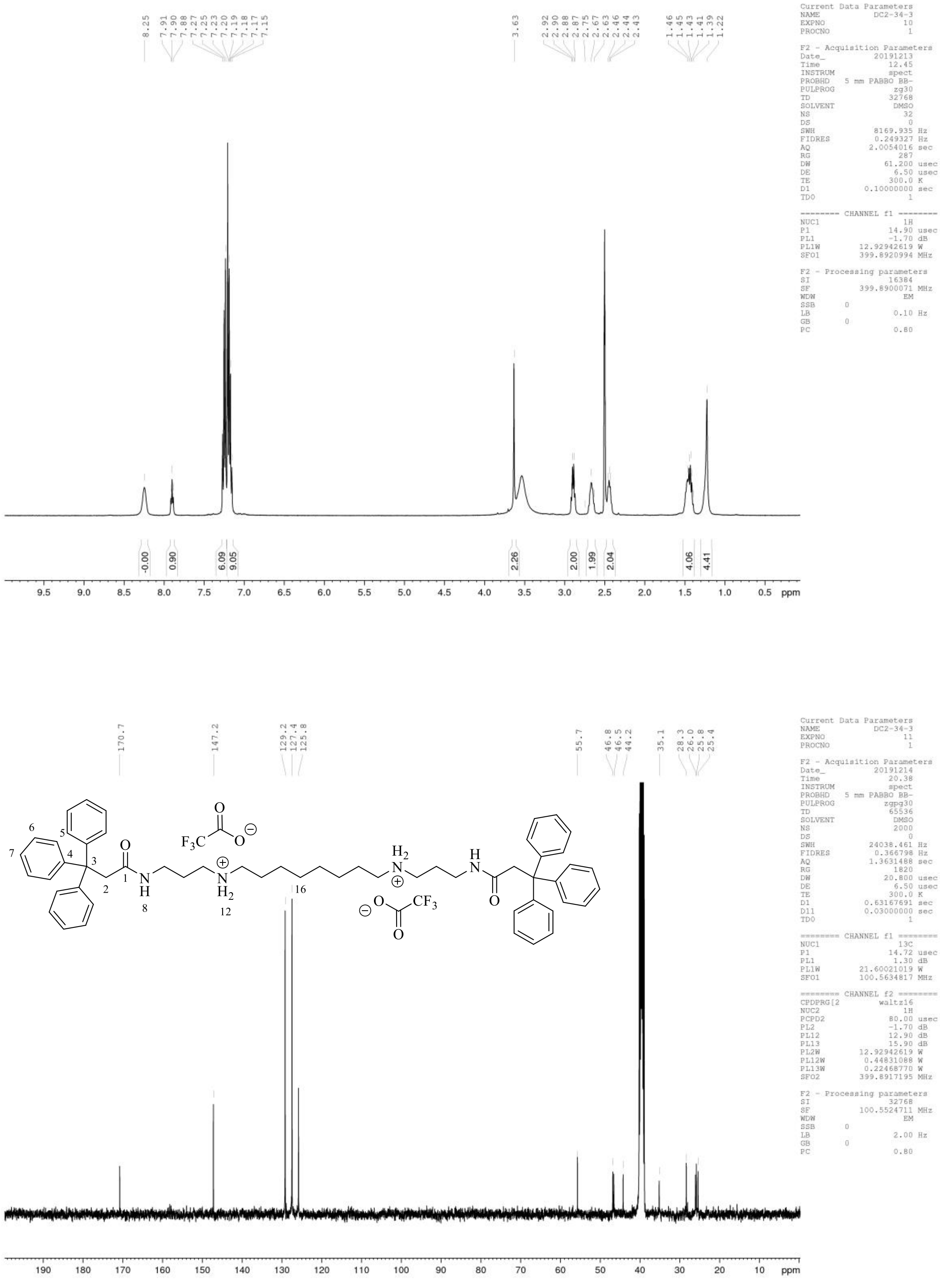

Figure **S42** ^{1}H NMR (DMSO-d_6, 400 MHz) and ^{13}C NMR (DMSO-d_6, 100 MHz) spectra for **18d**.

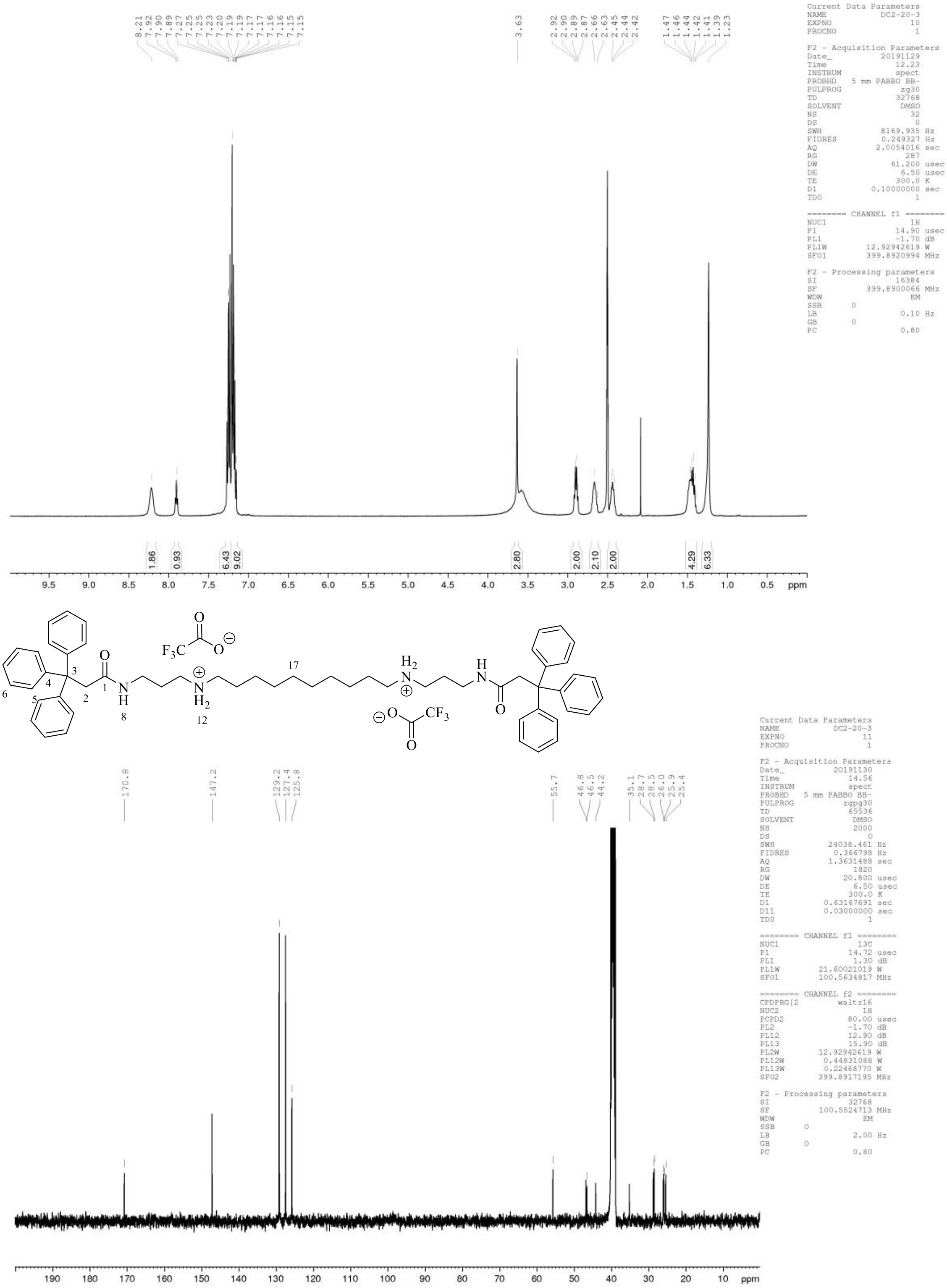

Figure **S43** ^{1}H NMR (DMSO-d_6, 400 MHz) and ^{13}C NMR (DMSO-d_6, 100 MHz) spectra for **18e**.

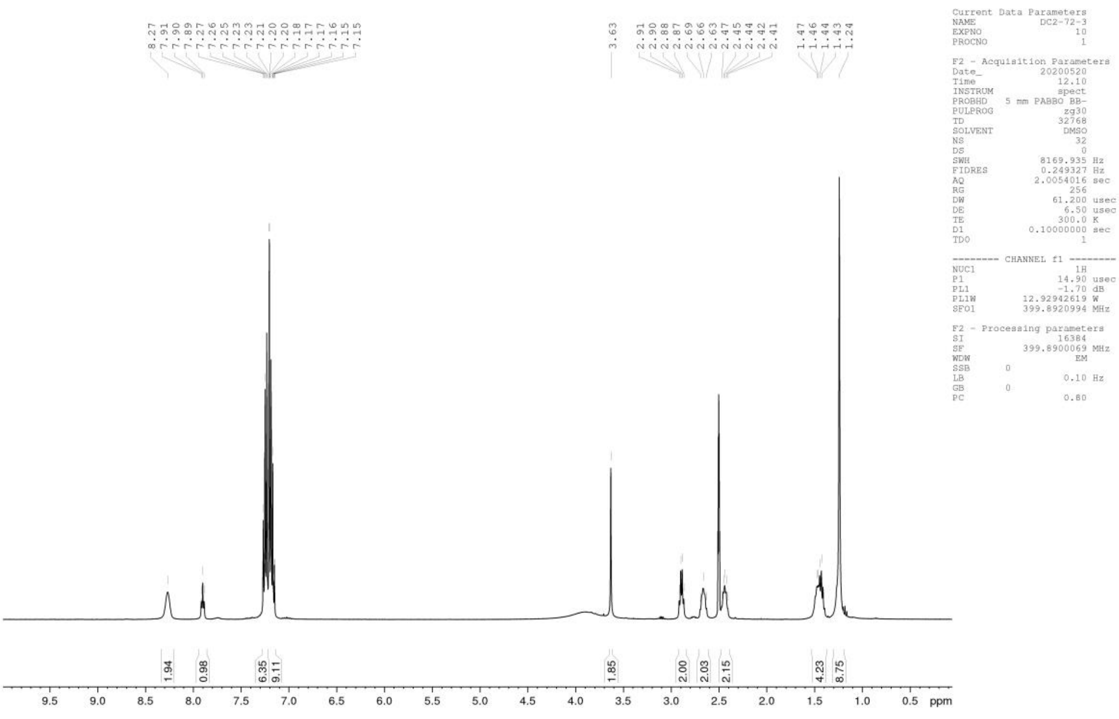

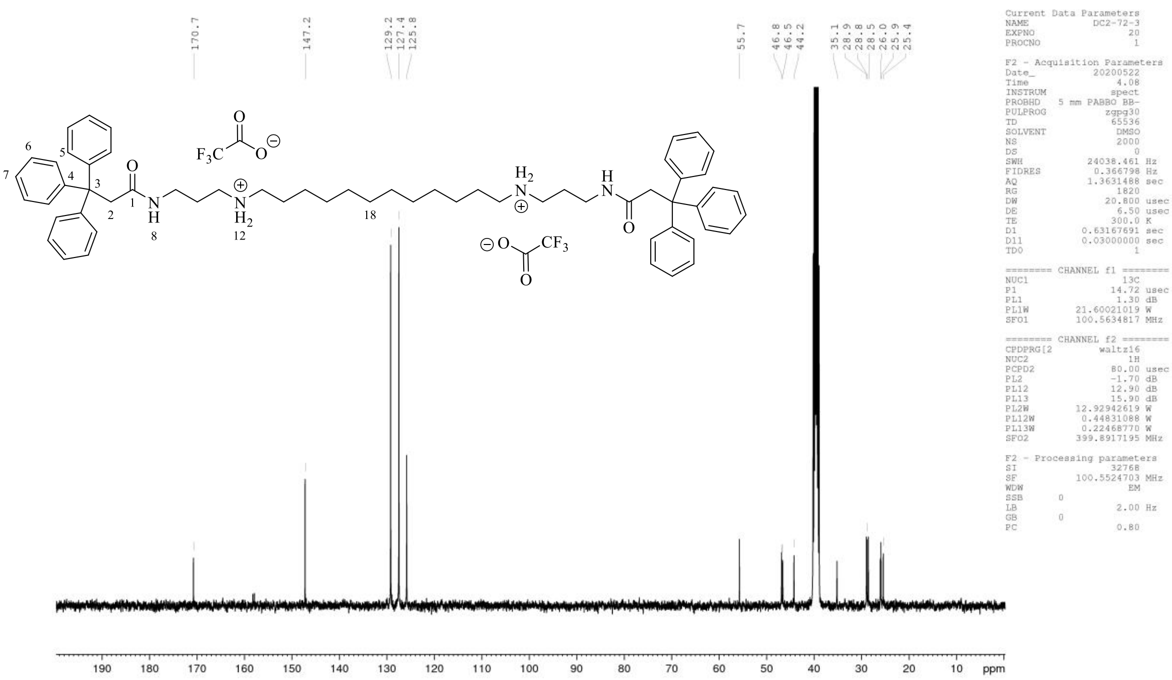

Figure **S44** ^{1}H NMR (DMSO-d_6, 400 MHz) and ^{13}C NMR (DMSO-d_6, 100 MHz) spectra for **18f**.
